# Dietary biochar enhances growth performance and feed efficiency in growing pigs evidence from integrated transcriptomic and proteomic analyses

**DOI:** 10.3389/fvets.2026.1863140

**Published:** 2026-07-02

**Authors:** Xiaoyu Zhang, Xiaohu Wu, Xiaoxiao He, Qing Wang, Haiyun Ma, Yongjie Duan, Xinghua Su, Shijun Bao

**Affiliations:** 1 College of Veterinary Medicine, Gansu Agricultural University, Lanzhou, Gansu, China; 2 Lanzhou Institute of Husbandry and Pharmaceutical Sciences, Chinese Academy of Agricultural Sciences (CAAS), Lanzhou, Gansu, China; 3 Shandong Lianxiang Zhirong Technology Co. Ltd., Yantai, Shandong, China; 4 Biochar (Beierka Qingdao) Intelligent Equipment Co. Ltd., Qingdao, Shandong, China

**Keywords:** biochar, growth performance, immunity, LC-MS, RNA-Seq

## Abstract

**Introduction:**

This study aimed to evaluate the effects of biochar as a feed supplement for growing pigs. Biochar, a carbon-rich material produced by pyrolysis of biomass under low- or no-oxygen conditions, is widely used in agriculture due to its porous structure and high adsorption capacity. Recently, it has gained attention as a potential feed additive for livestock because of its ability to bind toxins and gut microbiota modulation. Although several farm trials have reported improved animal productivity with biochar supplementation, the underlying biological function remains largely unclear.

**Methods:**

To identify candidate pathways associated with biochar supplementation, we performed transcriptomic (RNA-Seq) and proteomic (LC-MS) analyses on blood samples collected from pigs in 3% biochar-supplemented group and control group.

**Results:**

In this study, supplementing the basal diet with 3% biochar significantly reduced mortality (*P* < 0. 01) and improved the average daily gain (ADG) (*P* < 0. 01), leading to better overall growth performance in growing pigs. The results of transcriptomic (RNA-Seq) and proteomic (LC-MS) analyses demonstrated that biochar supplementation enhanced immune function. RNA-Seq analysis identified 524 differentially expressed genes (413 down-regulated and 111 up-regulated). Up-regulated genes were significantly enriched in the IL-17 signaling pathway and the complement and coagulation cascades, whereas the most prominently down-regulated pathway was related to the cytoskeleton in muscle cells. Proteomic analysis identified a total of 892 proteins, of which 192 were up-regulated and 186 were down-regulated in the biochar group. Pathways associated with cytokine-cytokine receptor interaction and complement and coagulation cascades were up-regulated in proteomic analysis. In contrast, down-regulated proteins were primarily enriched in cytoskeleton-related processes in muscle cells, Salmonella infection, and IgSF CAM signaling pathways.

**Discussion:**

These findings indicate that dietary biochar supplementation improves the health and performance of growing pigs, at least in part, by modulating immune-related pathways. The combined transcriptomic and proteomic results provide new molecular insights into how biochar exerts its beneficial effects as a feed additive.

## Introduction

With the continuous growth of the global population and rising living standards, the demand for both the quantity and quality of pork is steadily increasing. At the same time, the livestock industry is confronted with more complex infectious pathogens, widespread environmental toxins such as mycotoxins and pesticide residues, and the escalating global challenge of antibiotic resistance. In this context, nutritional strategies that can support natural disease resistance and enhance their overall resilience have attracted considerable research interest. Biochar, known for its strong toxin-adsorption capacity and ability to harbor beneficial microbes, shows promising potential to improve livestock health and help address the growing tension between rising pork demand and these production challenges.

Biochar is a carbon-rich material with a highly aromatic structure, produced by pyrolyzing biomass at high temperatures under low- or oxygen-limited conditions. Its surface is rich in oxygen-containing functional groups, which play a critical role in its physicochemical properties and biological activity (1). When used as a feed additive, the elemental composition, surface area, and porosity of biochar-properties largely determined by the type of biomass feedstock, pyrolysis temperature, and residence time are of primary importance (2). Strong links exist between these physicochemical characteristics and key pyrolysis parameters, particularly temperature and retention time. For feed-grade biochar, heating rates typically range from 7°C/min to 40°C/min, with residence time varying from 3 min to 12 h and pyrolysis temperatures between 350°C and 1,100°C (3). Higher pyrolysis temperatures promote the formation of more aromatic structures, with carbon yield reaching its peak at approximately 950°C (4). As temperature increases, volatile matter is progressively released from the biomass until around 700°C. This loss of organic volatiles leads to a corresponding increase in the relative proportion of ash (5). At temperatures below 350°C (the low-temperature pyrolysis zone), biochar retains a high content of oxygen-containing functional groups such as carboxyl and hydroxyl groups (6). During pyrolysis, these unstable groups undergo dehydration, decarboxylation, and acid-forming reactions involving cellulose and hemicellulose, contributing to the production of wood vinegar (7, 8). As these volatile compounds are released, the pH of the biochar gradually shifts from acidic to alkaline. At approximately 700°C, the crystalline regions transform into a more stable turbostratic graphene-like structure (9). In summary, heating rate, residence time, and pyrolysis temperature are essential factors to determine the characteristics of biochar which drive its biological function in feeds supplementation.

Due to its abundant surface functional groups and highly porous structure, biochar exhibits multiple beneficial properties in agricultural applications, including nutrient retention, probiotic delivery, soil remediation, and wastewater purification. In recent years, its application as a feed supplement in livestock production has shown considerable promise for improving both productivity and health, particularly in pigs. As industrialization of pig farming has been intensified, the negative impacts of dietary toxins such as heavy metals, mycotoxins, and pesticide residues have become increasingly significant (10). Heavy metals can disrupt normal biological functions by competing with essential metals and binding to native protein sites (11). As an effective toxin adsorbent, biochar mitigates these risks through ion exchange and surface adsorption mechanisms (12, 13). Its high adsorption capacity enables it to bind various contaminants in feed, including dioxins, mycotoxins, and certain pesticides, thereby helping to prevent abnormal digestion and metabolic disorders in farm animals (14, 15). In addition to toxin adsorption, biochar can modulate gut microbiota by promoting beneficial microbes and suppressing pathogenic bacteria. This modulation improves nutrient utilization, reduces methane emissions, and supports overall gut health which is a critical factor influencing the general wellbeing of livestock (16).

In livestock production field, biochar has been investigated as additives for enhancing the health status of farm animals because of its potential function in improving growth performance and resisting disease in various farm animal species. For poultry production, feeds with 3% biochar to Ross 308 broilers can enhance daily weight gain (DWG, *P* < 0.001) and improve feed conversion ratio (FCR, *P* < 0.001) during the grower-finisher period, minor microbiota shifts were observed at OTU (Operational Taxonomic Unit) level may explain the better growth performance (17). Another biochar supplement evaluative study on broilers similarly demonstrated that dietary biochar supplementation can improve growth performance and intestinal health in poultry, In addition, biochar supplementation enhanced antioxidant capacity and digestive enzyme activities, particularly amylase activity. Beneficial effects on gut microbiota were also observed, including increased populations of beneficial bacteria and reduced harmful bacterial abundance (18). Dietary inclusion of 2.5%, 5.0%, or 10% oak wood biochar for 21 days improved growth performance and feed efficiency, with the 5% level yielding the greatest increases in body weight gain (23%), feed intake (17%), and feed conversion ratio (5%) (19). The evaluative study on biochar supplementation in weaned piglets showed that 1% chestnut biochar supplementation could reduce fecal E. coli counts, decrease diarrhea incidence, improve protein digestibility, and alleviate oxidative stress markers which means that biochar could enhance intestinal health and physiological status (20). Additional research indicates that biochar supplementation can also promote intestinal villus development and reduce stress hormone levels (21). Metabolomic analysis (QTOF HPLC-MS/MS) identified hydroxybenzoic and succinic acids in the biochar water extract, similarly, Functional assays demonstrated that chestnut biochar inhibited the growth of pathogenic E. coli strains F18^+^ and F4^+^, with inhibition rates of 15.8% and 28.6%, respectively, and downregulated genes associated with quorum sensing and biofilm formation. Importantly, it did not negatively affect beneficial probiotic bacteria such as *Lactiplantibacillus plantarum* and *Limosilactobacillus reuteri*. The conclusion from this study indicated that chestnut biochar has potential as a functional feed additive that can suppress pathogenic bacteria while maintaining beneficial microbiota (22).

Although previous studies have demonstrated that dietary biochar can improve intestinal health, alleviate diarrhea, and enhance growth performance of farm animal, limited attention has been paid to the systemic biological responses induced by biochar supplementation which lead to the underlying systemic molecular changes in blood remain poorly understood. Blood proteomics and transcriptomics as powerful approaches for comprehensively evaluating physiological and molecular responses have been widely used for health evaluation. Transcriptomic analysis can reveal changes in gene expression related to immune regulation and inflammation (23), while proteomics directly reflects alterations in functional proteins associated with animal health status (24, 25). To date, there is still a lack of studies applying integrated blood multi-omics to investigate the effects of dietary biochar in pigs, leaving a critical knowledge gap in understanding its systemic mode of action. Therefore, this study aims to address this gap by applying integrated blood transcriptomic and proteomic analyses to elucidate the immune and health related response induced by biochar supplementation in pigs.

## Material and methods

### Experimental materials

The experimental protocol conducted on piglets was approved by the Experimental Animal Ethics Committee of Gansu Agricultural University (GSAU-Eth-VMC-2026-035). The piglets and biochar were provided by Xihua Muyuan Animal Husbandry Co., Ltd. and Biochar (Beierka Qingdao) Intelligent Equipment Co., Ltd., respectively, and the trial was carried out at the experimental farm of Shandong Lianxiang Zhirong Technology Co., Ltd which located in Yantai city, Shandong province, China. All piglets were from the same batch, with the same age and management protocol and were reared in a building equipped with a mechanical ventilation system and a cooling pad system with the same rearing density. The initial rearing temperature for weaned piglets was 28°C, which was decreased by 1°C every two weeks until stabilizing at 18–24°C.

### Physicochemical properties of biochar

The biochar used in this experiment was made from apple tree wood with 20°C/min of heating rate, 650°Cof final pyrolysis temperature, 10 min of residence time. The physicochemical properties of the biochar used in this study were as follows. It had a pH of 8.61, indicating weak alkalinity. The electrical conductivity (EC) was 336 μS/cm. The ash content was 3.08%, indicating low impurity levels. The total carbon (C) content was 76.10%. The specific surface area was 108.6 m^2^·g^−1^, indicating high adsorption capacity and microbial attachment potential. The hydrogen (H) and nitrogen (N) contents were 4.18% and 0.28%, respectively. The H/C ratio was 0.66, reflecting a relatively high degree of aromaticity and structural stability (10). The physicochemical properties of the biochar were listed in Table 1.

**Table 1:** The physicochemical properties of the biochar.

Essential property	Data	Essential property	Data
pH	8.61	Specific surface area	108.6 m^2^·g^−1^
Electrical conductivity(EC)	336 μS/cm	Hydrogen (H) content	4.18%
Ash content	3.08%	Nitrogen (N) contents	0.28%
Total carbon (C) content	76.10%	H/C ratio	0.66

### Experimental animals and design

A total of 2,000 experimental crossbred weaning piglets (Duroc × Landrace × Yorkshire), aged approximately 26 days, were divided into Group A and Group B, with 1,000 piglets in each group. The initial weight was weighted by pen and the daily feeds consumption was recorded. Group A is experimental group (EG) in which the pigs were fed basal feeds supplemented with 3% biochar, while group B is control group (CG) in which the pigs were fed basal feeds. The feed formulation are shown in Table 2. The 1,000 piglets in each group were reared in 40 pens (25 piglets per pen), and each pen was numbered. Each group was further divided into five subgroups, with each subgroup consisting of 8 pens (*n* = 5), 200 piglets in one replicate. Four male piglets with similar body weights were selected from four randomly chosen pens in each group to ensure baseline homogeneity and minimize initial variation among experimental animals (EG: 6.47 ± 0.11 kg; CG: 6.51 ± 0.12 kg; *P* > 0.05). Subsequently, the four piglets in each group were individually housed in separate pens. Daily feed intake was recorded throughout the experimental period (43 days of age to 145 days of age). From the 43 days of age to 145 days of age, cough pigs and diarrhea pigs were recorded in each subgroup. Coughing was assessed daily at 14:00, pigs were gently driven to stand and move within each subgroup (8 pens) to stimulate potential respiratory responses, and coughing events were recorded by visual observation for 1 min per subgroup. Pigs were marked individually, and those exhibiting coughing for two consecutive days were classified as coughing pigs,the data were organized in Excel for subsequent statistical analysis (26). Piglets were provided *ad libitum* access to feed and water throughout the experiment. Diarrhea was evaluated daily at 9:00 a.m. based on fecal consistency using a 4 point scoring system: 0 = normal feces, 1 = soft feces, 2 = liquid feces, and 3 = watery feces. Piglets with fecal scores of 2 or 3 were considered diarrheic. The diarrhea rate of each stage (43–95 days of age and 96–145 days of age) was calculated on a subgroup basis as follows: diarrhea rate (%) = (number of diarrheic piglets / total number of observed piglets) × 100. The data were organized in Microsoft Excel for subsequent statistical analysis (27). Mortality was recorded daily throughout the experimental period for each group. The number of dead animals was documented, and the mortality rate was calculated as the percentage of dead pigs relative to the initial number of pigs in each subgroup. At the age of 172, the marketing weight was weighted by pen (*n* = 32).

**Table 2:** Feed formulation for phase-specific diet.

Ingredient	Starter (%)	Grower (%)	Finisher (%)
Corn (Grade 2)	54.3	55.62	54
Wheat flour	15	15	15
Wheat bran		3.1	12.2
Wheat germ	5	5	5
46% soybean meal	17.29	12.85	5.78
Corn DDGS		5	5
Extruded soybean	4		
Soybean oil	0.75		
37% limestone powder	0.72	1.04	1.22
Dicalcium phosphate	1.13	0.51	
Salt	0.4	0.4	0.4
70% L-Lysine	0.79	0.89	0.88
99% DL-methionine	0.11	0.08	0.07
98.5% L-threonine	0.17	0.16	0.1
98% L-tryptophan	0.02	0.03	0.03
Compound enzyme	0.02	0.02	0.02
Starter premix	0.3		
Grower-finisher premix		0.3	0.3

The vitamin-trace mineral premix contained fat-soluble vitamins (vitamin A, vitamin D_3_, vitamin E, and vitamin K_3_) and B-group vitamins (vitamin B_1_, vitamin B_2_, vitamin B_6_, vitamin B_12_, niacin, D-calcium pantothenate, folic acid, and biotin), as well as inorganic trace minerals including Fe, Cu, Zn, Mn, Se, I, and Co. The premix formulations were adjusted according to the phase-specific nutrient requirements recommended by NRC (57), resulting in higher inclusion levels of vitamin E, biotin, and zinc in the starter phase compared with the grower-finisher phase. The enzyme additive consisted mainly of phytase and xylanase, which are commonly used feed-grade enzymes in China.

Average daily gain (ADG) was calculated on a pen basis from 26 to 172 days of age (*n* = 32), excluding the 8 pens from which pigs were randomly selected for omics analysis. Feed conversion ratio (FCR) was calculated at the group level over the same period. The coughing incidence and diarrhea rate were calculated during the period from 43 to 145 days of age. In addition, the individual ADG and FCR of the eight pigs selected for omics blood sampling were calculated from 43 to 145 days of age. All data were analyzed using SPSS software (26.0 version), and statistical differences between groups were determined based on *P* values, with *P* < 0.05 considered statistically significant and *P* < 0.01 considered highly significant.

### Blood collection and sample pre-treatment

Blood collection was conducted starting at 145 days of age. The collection start at 7:00 a.m. under fasting conditions from eight pigs in the biochar-treated group and eight pigs in the control group via the anterior vena cava using a vacuum blood collection tube. For each pig, two 5 mL blood samples were collected: one in an anticoagulant-free tube for proteomic analysis and one in an anticoagulant-containing tube for transcriptomic analysis.After collection, the anticoagulant-free tubes were placed obliquely in a 4°C constant-temperature refrigerator for 30–60 min to allow serum separation, after which the serum was carefully transferred into cryogenic storage tubes. For samples collected in anticoagulant-containing tubes, the tubes were gently inverted several times immediately after collection to ensure proper mixing. Subsequently, 2 mL of whole blood was transferred into a pre-prepared cryogenic tube containing 6 mL of TRIzol reagent in a laminar flow hood and mixed thoroughly as soon as possible. All serum samples and TRIzol-preserved blood samples were transported to the laboratory under 4°C conditions and subsequently stored at −80°C until further proteomic and transcriptomic analyses.

### Transcriptomics analysis

Total RNA was extracted from blood samples stored at −80°C using TRIzol™ Reagent (Invitrogen, Cat. No. 15596026), and RNA concentration and purity were assessed using a NanoDrop 2000. Samples with RNA concentration > 35 ng/μL, OD260/OD28 ≥ 1.8, OD260/OD230 ≥ 1.0, and total RNA ≥ 1 μg were selected. RNA integrity was evaluated using agarose gel electrophoresis and an Agilent 2100 Bioanalyzer. mRNA was enriched using oligo- (dT)-conjugated magnetic beads, followed by fragmentation (~300 bp), first-and second-strand cDNA synthesis, end repair, A-tailing, adapter ligation, and PCR amplification (15 cycles) to construct the cDNA library (28). Library quality was verified by agarose gel electrophoresis, quantified using the PicoGreen method, and pooled for sequencing. Sequencing was performed on the Illumina NovaSeq 6000 platform using a paired-end 2 × 150 bp strategy after cluster generation by cBot. Raw reads were processed for transcriptomic analysis, including read counting using featureCounts (29) and normalization using FPKM. Sample correlation (Pearson R^2^) and principal component analysis (PCA) were used to assess reproducibility. Differentially expressed genes (DEGs) were identified using edgeR/DESeq2 (30) with padj <0.05, followed by GO and KEGG enrichment (31) analysis using clusterProfiler and gene set enrichment analysis (GSEA). All of the results were visualized by using R. Alternative splicing events were analyzed by using rMATS (FDR <0.05, |IncLevelDifference| > 0.02), with the top significant events visualized using rmats2sashimiplot.

### Proteomic analyses

Serum samples were thawed on ice and centrifuged at 15,000 × g for 10 min at 4°C, after which the supernatant was collected and high-abundance proteins were depleted using Pierce™ Top 14 Abundant Protein Depletion Spin Columns. Protein concentration was determined using the BCA assay. Equal amounts of protein from each sample were subjected to TCA precipitation, acetone washing, and resuspension in 100 mM TEAB, followed by sonication. Proteins were reduced with 5 mM DTT (56°C, 30 min) and alkylated with 15 mM iodoacetamide (room temperature, dark, 15 min), then digested overnight with trypsin (2%, 37°C). The resulting peptides were acidified (10% TFA), desalted using Strata X cartridges, and quantified using the Pierce™ Quantitative Peptide Assay before vacuum freeze-drying.

Peptide samples were analyzed using a nano-flow Vanquish Neo UHPLC system coupled with an Astral high-resolution mass spectrometer (Thermo Fisher Scientific) operating in data-independent acquisition (DIA) mode (32). MS1 scans were acquired over 380–980 m/z at 240,000 resolution (m/z 200), with a normalized AGC target of 500% and a maximum injection time of 5 ms. DIA MS/MS was performed using 299 isolation windows of 2 m/z, with HCD energy set to 25 eV, AGC target at 500%, and maximum injection time of 3 ms.

For bioinformatics analysis, identified proteins were functionally annotated using eggNOG-mapper (v2.0) (33) with the EggNOG 5.0 database for Gene Ontology classification, PfamScan for protein domain annotation, BLASTP against the KEGG (34) database for pathway analysis (E-value ≤ 1e−4), and WoLF PSORT (35) for subcellular localization prediction, with additional COG annotation. Functional enrichment analyses of GO, KEGG, and protein domains were performed using Fisher's exact test (*P* < 0.05), followed by log10 transformation, Z-score normalization, hierarchical clustering, and heatmap visualization using the pheatmap package in R.

### Integrated transcriptomic and proteomic analysis

After transcriptomic and proteomic data being acquired, an integrated multi-omics analysis was performed to assess transcriptional and translational consistency. DEGs and DEPs were identified separately and mapped using unified gene symbols. Overlap analysis was conducted to identify shared and unique molecules, which were visualized using a Venn diagram to evaluate transcriptome-proteome concordance.

To evaluate the relationship between mRNA and protein expression, an integrated RNA-protein correlation analysis was performed based on matched DEGs and DEPs using log2 fold change (log2FC) values. Pearson correlation analysis was used to assess overall consistency between transcriptomic and proteomic profiles. A quadrant-based model was further applied to classify genes into concordant (up/up, down/down) and discordant (up/down, down/up) regulation patterns, and results were visualized using a quadrant scatter plot.

In addition, Kyoto Encyclopedia of Genes and Genomes (KEGG) pathway enrichment analysis was performed separately for transcriptomic and proteomic datasets. Overlapping significantly enriched pathways between the two omics layers were identified and used to construct a shared pathway set. The integrated KEGG pathway profiles were visualized using hierarchical clustering to generate a heatmap, illustrating the common functional pathways between the transcriptome and proteome.

### Immune–growth trait correlation

A correlation analysis was performed to explore the relationships between immune-related signatures, serum protein abundance, and growth performance in pigs. Two immune-related composite scores, including the Pig IL17 score and Pig Defense Response Score, together with six serum proteins (*SAA2, CRP, MYH1, S100A12, SLPI, and TIMP3*), were selected for association analysis.

Average daily gain (ADG) and feed conversion ratio (FCR) were used as phenotypic indicators of growth performance. All immune scores, protein abundance values, and performance traits were standardized prior to analysis. Pearson correlation coefficients were calculated to evaluate the relationships between immune-related variables and growth traits (ADG and FCR). Correlation analysis was performed using R software, and results were visualized as correlation heatmaps and/or scatter plots to identify potential immune-growth regulatory associations.

### qRT-PCR validation for transcriptomic data

Total RNA was extracted from whole blood samples using TRIzol reagent. After synthesizing the first-strand cDNA, qRT-PCR was performed to detect the OAS1 and RAG1 genes, with GAPDH as the internal reference. The expression levels were calculated using the ΔΔCT method, and all experiments were performed in triplicate.

### Western blot validation for proteomic data

Total protein was extracted from serum samples and quantified by BCA assay. Equal amounts of protein were separated by SDS-PAGE, transferred to PVDF membranes, and incubated with primary antibodies against SLPI, SAA2, and β-actin (loading control), followed by HRP-conjugated secondary antibodies. Protein bands were visualized by ECL, and band intensity was quantified using ImageJ software.

## Result

### Growth performance

**Figure 1:**
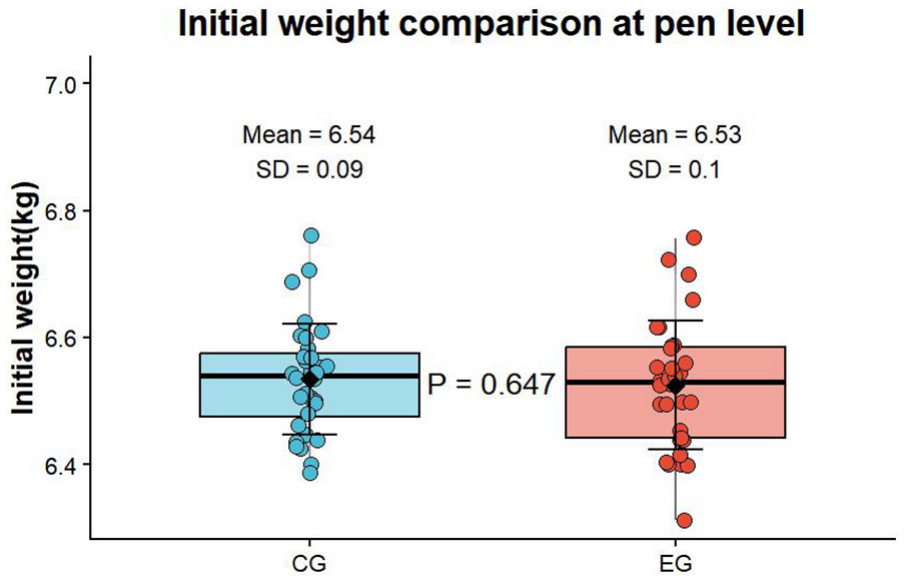
Initial weight comparison.

The average initial body weights of pigs in the whole experiment group (EG) and the whole control group (CG) were 6.55 kg and 6.57 kg, respectively. By comparing the weight of 32 pens piglets in EG group and CG group, there was no significant difference between the two groups (*P* > 0.05), confirming that the groups were comparable at baseline (Figure 1). At 172 days of age, the final market body weight of pigs in the EG group was higher than that in the CG group, with average weights of 128.1 kg and 122.1 kg, respectively, indicating an improvement in growth performance in the EG group. In terms of feed intake, the total feed consumption per pig was comparable between the two groups, with 279.0 kg in the EG group and 279.9 kg in the CG group, suggesting that the difference in body weight was not driven by increased feed intake. Accordingly, the EG group showed a lower feed conversion ratio (FCR = 2.30) compared with the CG group (FCR = 2.42), indicating that enhanced growth performance is induced by improving feed utilization efficiency and higher health status (Table 3).

**Table 3:** Overall FCR throughout the weaning-to-finishing period.

Group	Initial weight (kg)	Marketing weight (kg)	Weight gain (kg)	Feed consumption (kg)	FCR
EG	6.55	128.1	121.55	279.0	2.30
CG	6.57	122.1	115.53	279.9	2.42

FCR, Feed Conversion Ratio; EG, Experiment Group (biochar treatment); CG, Control Group.

**Figure 2:**
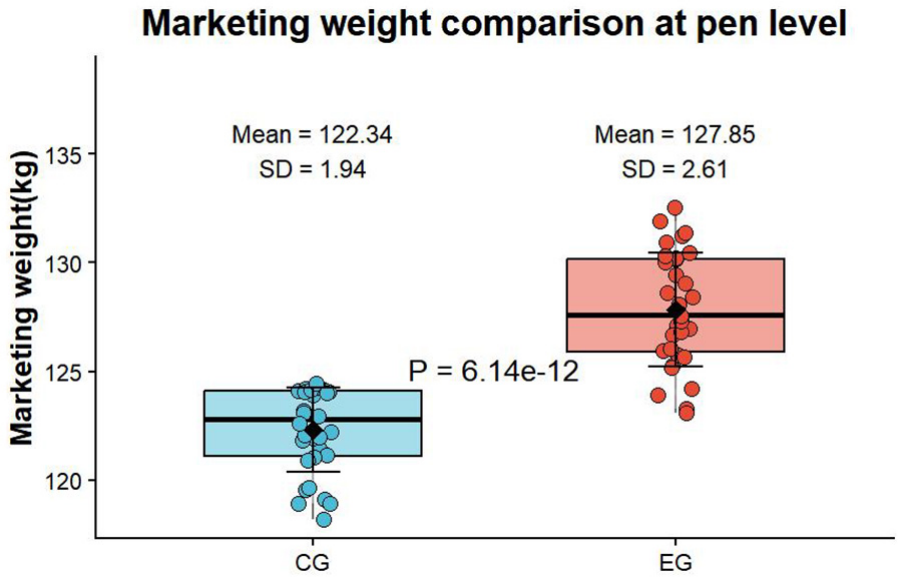
Marketing weight comparison.

**Figure 3:**
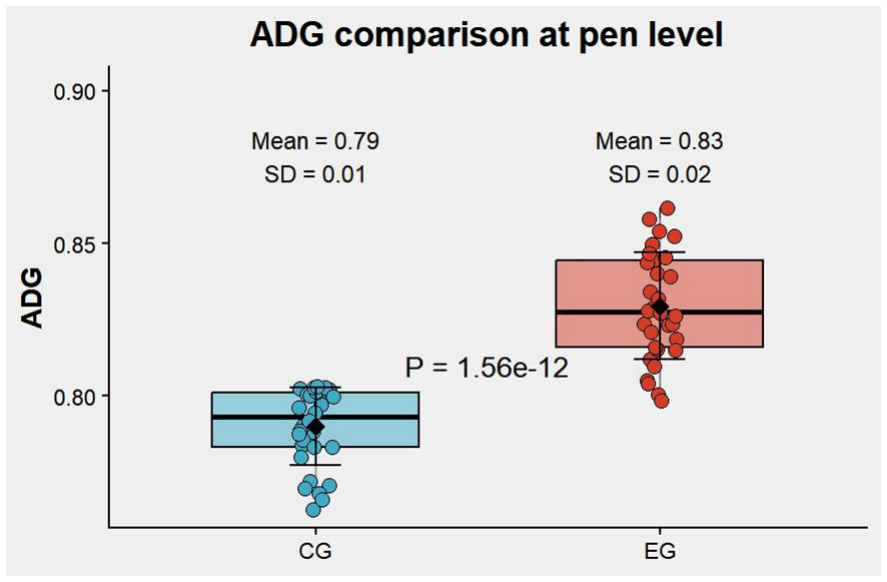
ADG comparison.

During the period from 26 to 172 days of age, the difference of growth performance was evaluated using 32 pens (32/40) per treatment excluding the pen number (8/40) from which omics-sampling pigs were selected. The initial body weights were comparable between the two groups, with average values of 6.53 kg in the EG group and 6.54 kg in the CG group. At market age (172 days of age), pigs in the EG group exhibited a significant higher final body weight than those in the CG group (Figure 2), reaching 127.85 kg compared with 122.34 kg (*P* < 0.01). In addition, the average daily gain (ADG) of pigs in the EG group was higher than that of the CG group, reaching 0.83 kg/day compared with 0.79 kg/day (*P* < 0.01), indicating the significant difference in growth performance of the EG group and CG group (Figure 3).

**Figure 4:**
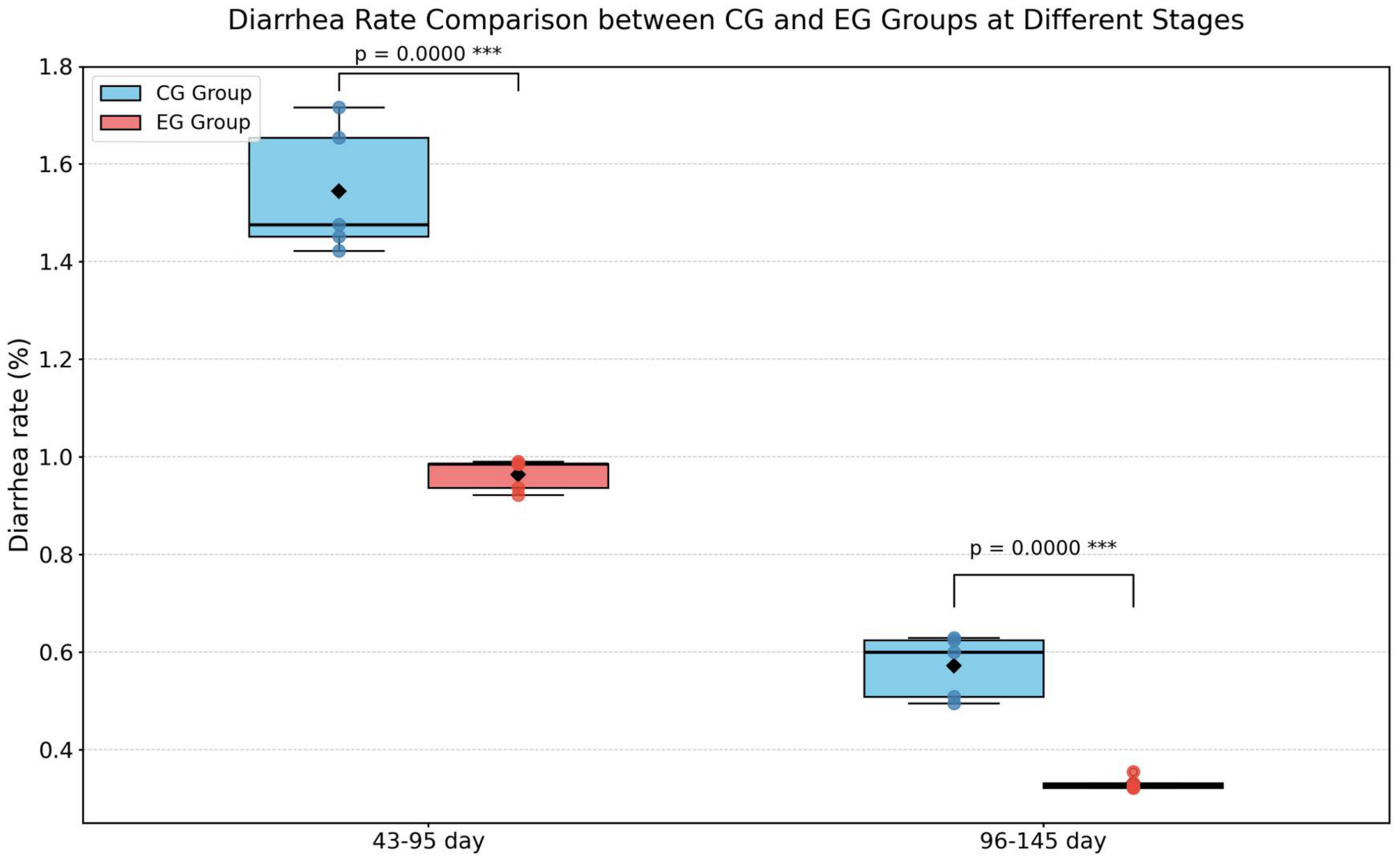
Diarrhea rate comparison. The triple asterisk symbol (***) presented an extremely significant statistical difference, corresponding to *P* < 0.001.

During the 43–95 days of age period, the diarrhea rate in the EG group was significantly lower than that in the CG group, with mean values of 0.97% and 1.54%, respectively (*P* < 0.01). In the 96–145 days of age period, diarrhea incidence decreased markedly in both groups, significance remained lower in the EG group compared with the CG group (0.33% vs. 0.58%, *P* < 0.01) (Figure 4).

**Figure 5:**
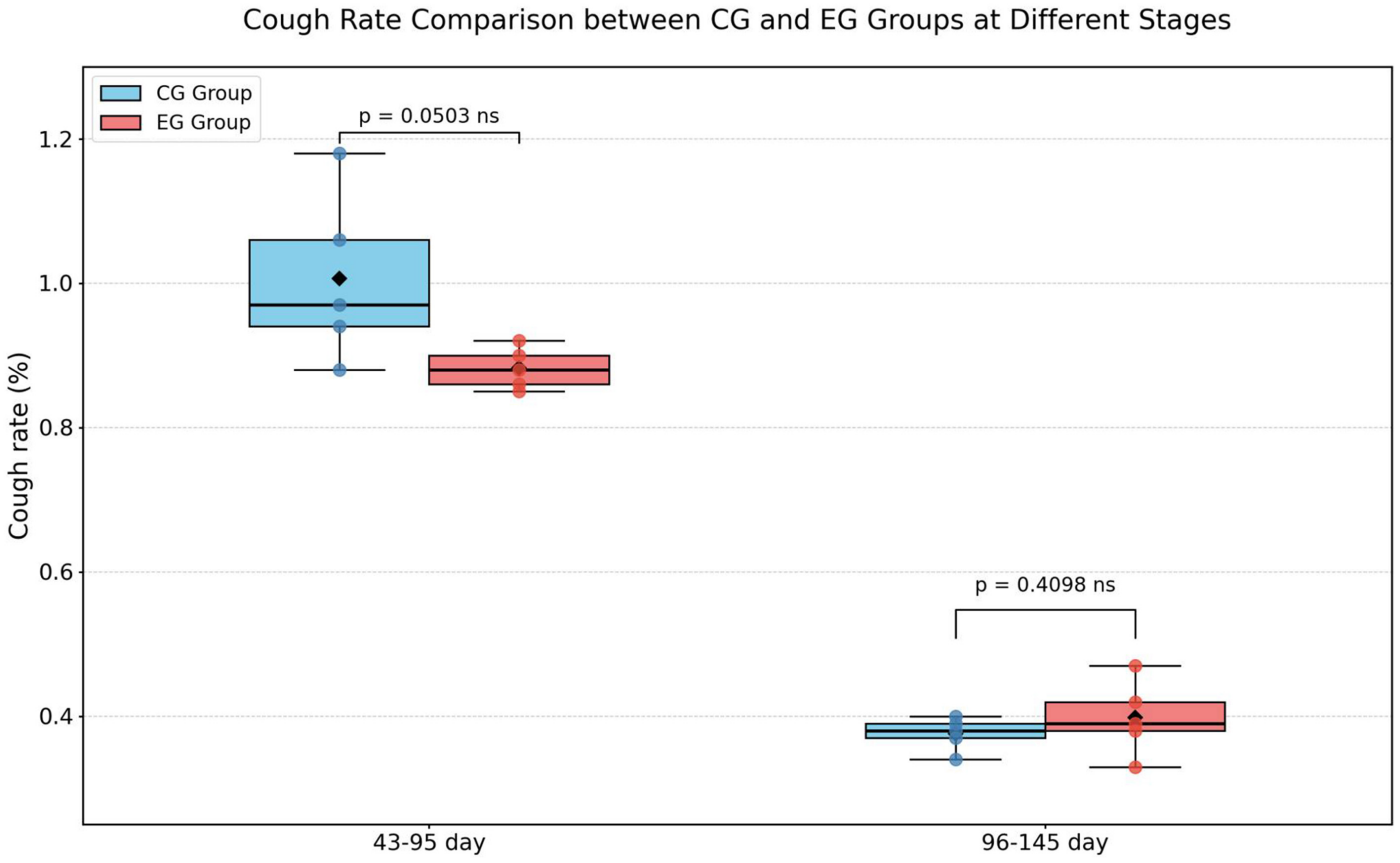
Cough rate comparison.

For the coughing rate, no significant differences were observed between the two groups during the 43–95 days of age period, although the EG group showed a slightly lower mean value compared with the CG group (0.88% vs. 1.01%, *P* > 0.05). In the 96–165 days of age period, coughing rates decreased in both groups, with values of 0.40% in the EG group and 0.38% in the CG group, and no significant difference was detected between treatments (*P* > 0.05). Although the differences were not statistically significant, the EG group showed a numerically lower coughing rate during the early stage, while both groups converged to similar levels in the later stage (Figure 5).

**Figure 6:**
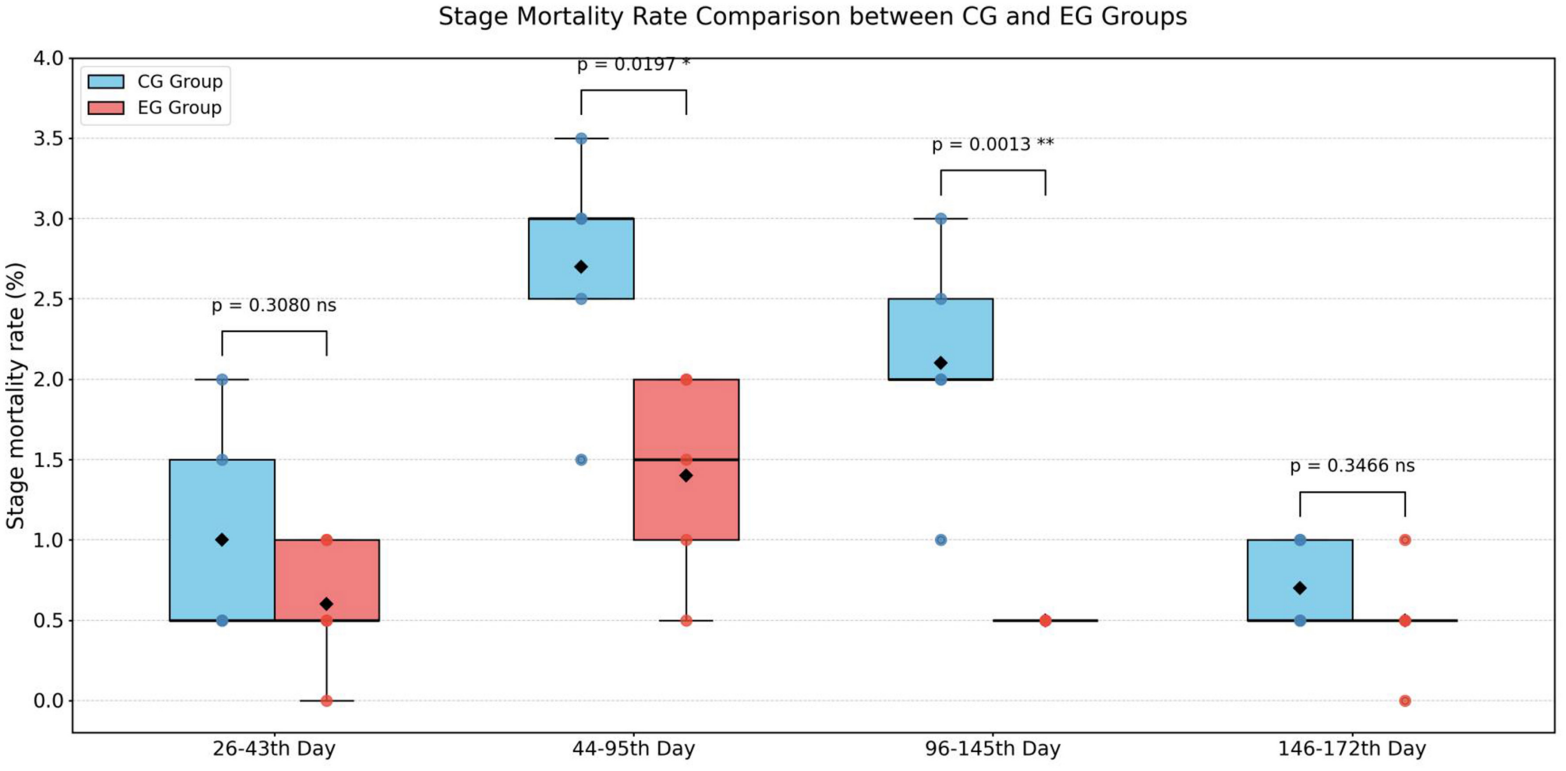
Staged mortality rate comparison. The single asterisk (*) represents *P* < 0.05 which means differences, and double asterisks (**) represent *P* < 0.01, denoting significant differences.

The mortality rates across different growth stages are presented in Figure 6. During the 26–43 days of age period, no significant difference in mortality rate was observed between the EG and CG groups (0.6% vs. 1.0%, *P* > 0.05). Nonetheless, differences between the two groups became evident during the subsequent growth stages. In the 43–95 days of age period, the mortality rate was significantly lower in the EG group compared with the CG group (1.4% vs. 2.7%, *P* < 0.05). A more pronounced difference was observed during the 95–145 days of age period, where the EG group showed a markedly reduced mortality rate compared with the CG group (0.5% vs. 2.1%, *P* < 0.01). In the 145–172 days of age period, mortality remained low in both groups, with no significant difference detected (0.5% vs. 0.7%, *P* > 0.05).

**Figure 7:**
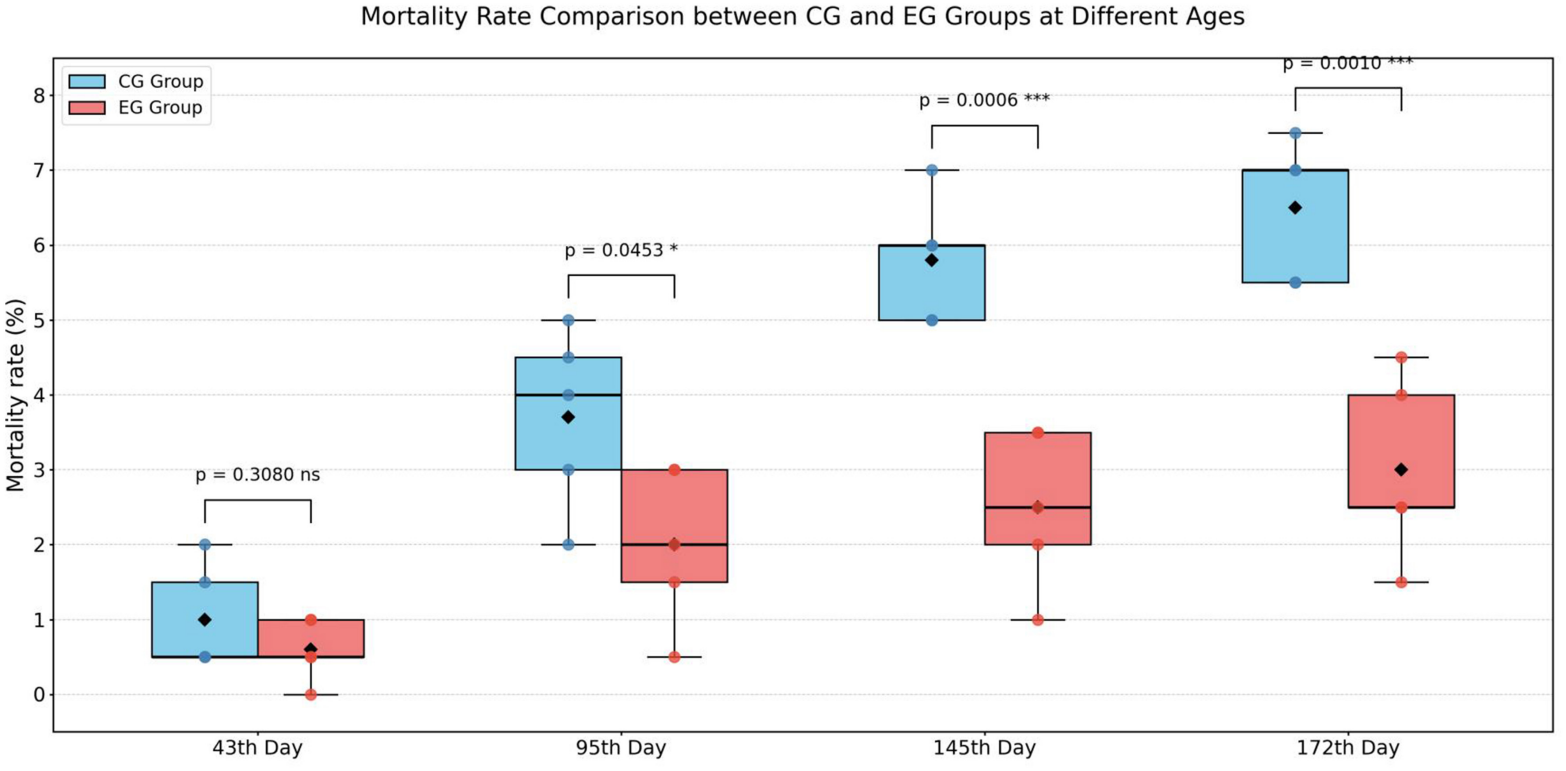
Cumulative mortality rate comparison. *p < 0.05; ***p < 0.001.

Consistently, the cumulative mortality rate showed an increasing divergence between the two groups over time. At 43 days of age, cumulative mortality was comparable between EG and CG groups (0.6% vs. 1.0%, *P* > 0.05). By 95 days of age, the CG group exhibited a significantly higher cumulative mortality than the EG group (3.7% vs. 2.0%, *P* < 0.05). This difference further expanded at 145 days of age (5.8% vs. 2.5%, *P* < 0.01) and remained significant at 172 days of age (6.5% vs. 3.0%, *P* < 0.01). Generally, the CG group showed a continuous increase in cumulative mortality throughout the growing period, whereas the EG group maintained a slower increasing trend, revealing that the biochar supplement in feeds effectively reduced mortality rate across the production cycle (Figure 7).

Taken together, dietary treatment improved growth performance and health status of pigs during the 26–172 days of age period. Pigs in the EG group showed higher final body weight and average daily gain compared with the CG group, while feed efficiency was improved without increasing feed intake. In addition, the EG group exhibited consistently lower incidence of diarrhea and reduced risk of mortality across key growth stages, particularly during the mid-growing period. Collectively, these results suggesting that the 3% biochar supplement in feed effectively enhanced growth performance and health outcomes in growing-finishing pigs (Table 4).

**Table 4:** Growth performance and health-related indicators of pigs.

Item compared	Age (day)	n	EG mean ±SD	CG mean ±SD	*t*-value	*p*-value
Ave. initial weight per pen	26–172th day	32	6.53 ± 0.10	6.54 ± 0.09	−0.42	0.6747
Ave. marketing weight per pen		32	127.85 ± 2.61	122.34 ± 1.94	9.59	6.14 × 10^−12^
ADG		32	0.83 ± 0.02	0.79 ± 0.01	10.33	1.56 × 10^−12^
Cumulative mortality rate of each sub group (200 heads)	43th day	5	0.60 ± 0.42	1.00 ± 0.71	−1.09	0.3080
	95th day	5	2.00 ± 1.06	3.70 ± 1.20	−2.37	0.0453
	145th day	5	2.50 ± 1.06	5.80 ± 0.84	−5.46	0.0006
	172th day	5	3.00 ± 1.22	6.50 ± 0.94	−5.08	0.0010
Staged mortality rate of each sub group (200 heads)	26–43th day	5	0.60 ± 0.42	1.00 ± 0.71	−1.09	0.3080
	44–95th day	5	1.40 ± 0.65	2.70 ± 0.76	−2.91	0.0197
	96–145th day	5	0.50 ± 0.00	2.10 ± 0.74	−4.82	0.0013
	146–172th day	5	0.50 ± 0.35	0.70 ± 0.27	−1.00	0.3466
Diarrhea incidence rate of each sub group (200 heads)	43–95th day	5	0.97 ± 0.03	1.54 ± 0.13	−9.44	0.0004
	96–145th day	5	0.33 ± 0.02	0.58 ± 0.06	−8.16	0.0007
Cough incidence rate of each sub group (200 heads)	43–95th day	5	0.88 ± 0.03	1.01 ± 0.12	−2.30	0.0503
	96–145th day	5	0.40 ± 0.05	0.38 ± 0.02	0.87	0.4098

EG, Experiment Group (biochar treatment); CG, Control Group; SD, Standard Deviation; n, the number of replicates.

### Identification of differentially expressed genes

#### Quality control

**Figure 8:**
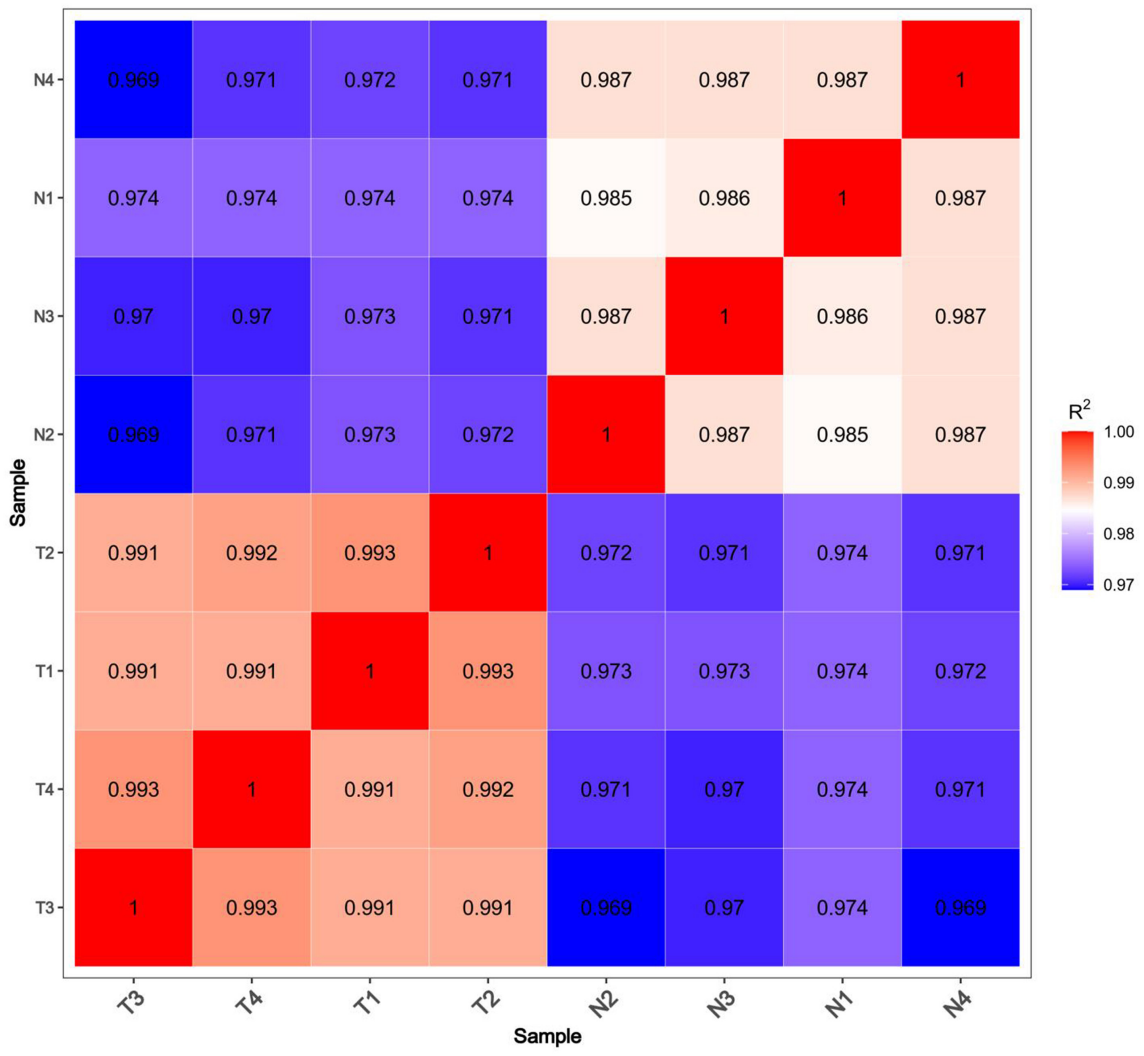
Correlation heatmap of FPKM expression levels.

**Figure 9:**
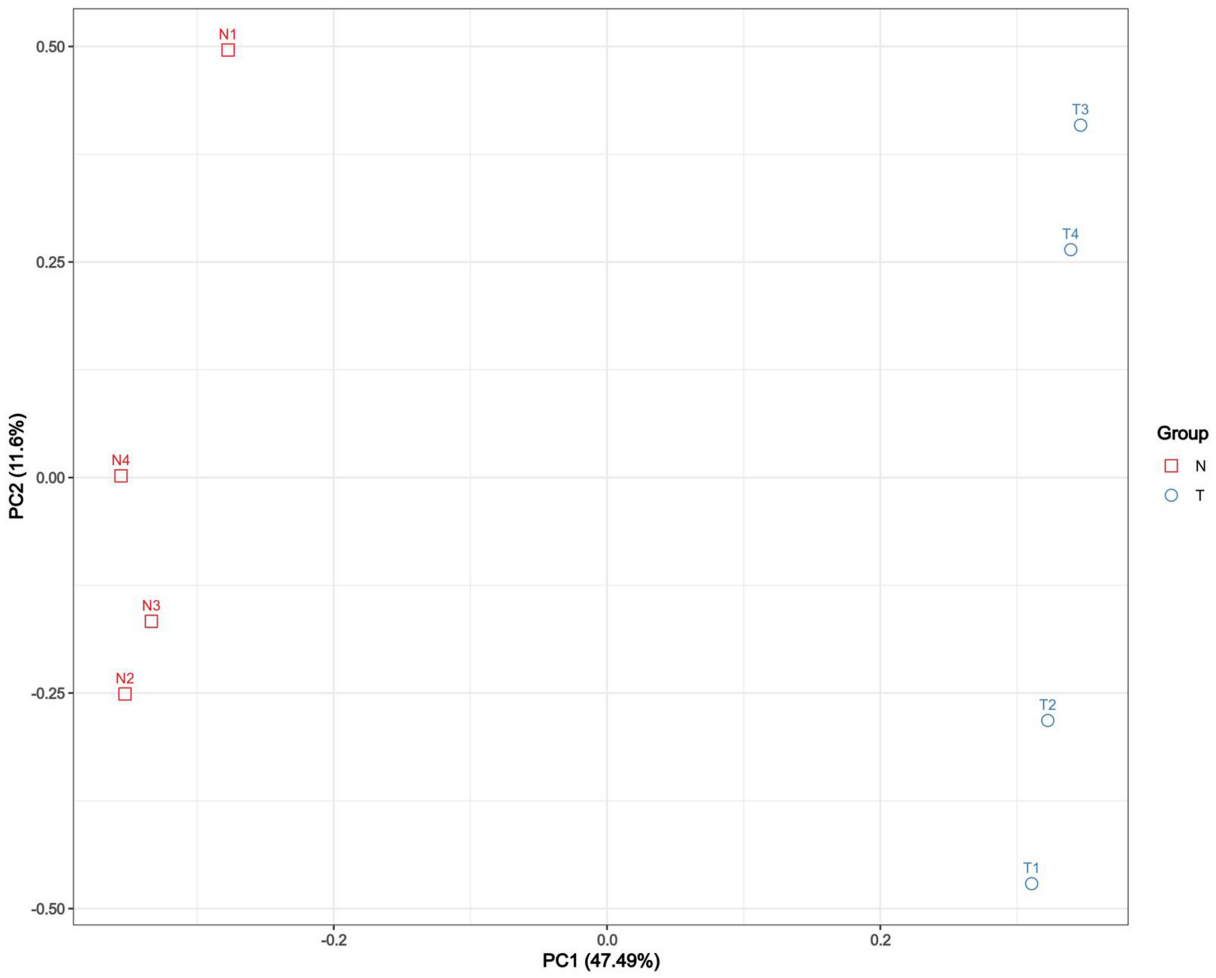
PCA plot of FPKM expression.

High-quality cDNA libraries were confirmed by read distribution and sequencing saturation analyses. Mapped reads were evenly distributed across transcripts, indicating effective RNA fragmentation and minimal positional bias (Supplementary Figures 1–8). Sequencing saturation analysis showed that relative errors in RPKM values decreased and reached a plateau with increasing sequencing depth, demonstrating sufficient transcriptome coverage (Supplementary Figures 9–16). Pearson correlation analysis revealed strong consistency among biological replicates (R^2^≈1; Figure 8), while PCA showed clear separation between experimental groups and tight clustering of replicates, indicating high data reproducibility and reliability for downstream analyses (Figure 9).

#### Analysis of different expressed genes

**Figure 10:**
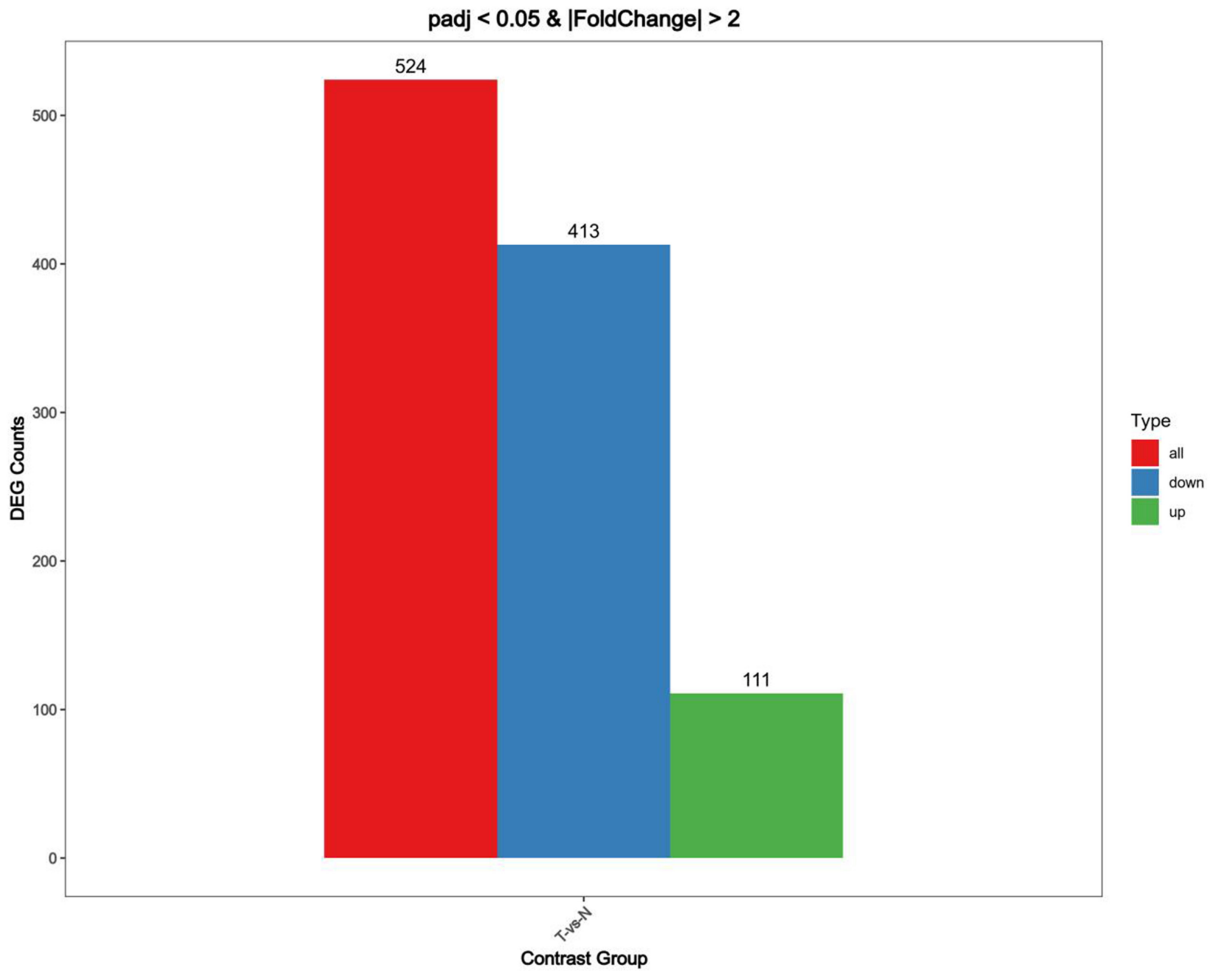
Bar plot of DEGsFigure.

**Figure 11:**
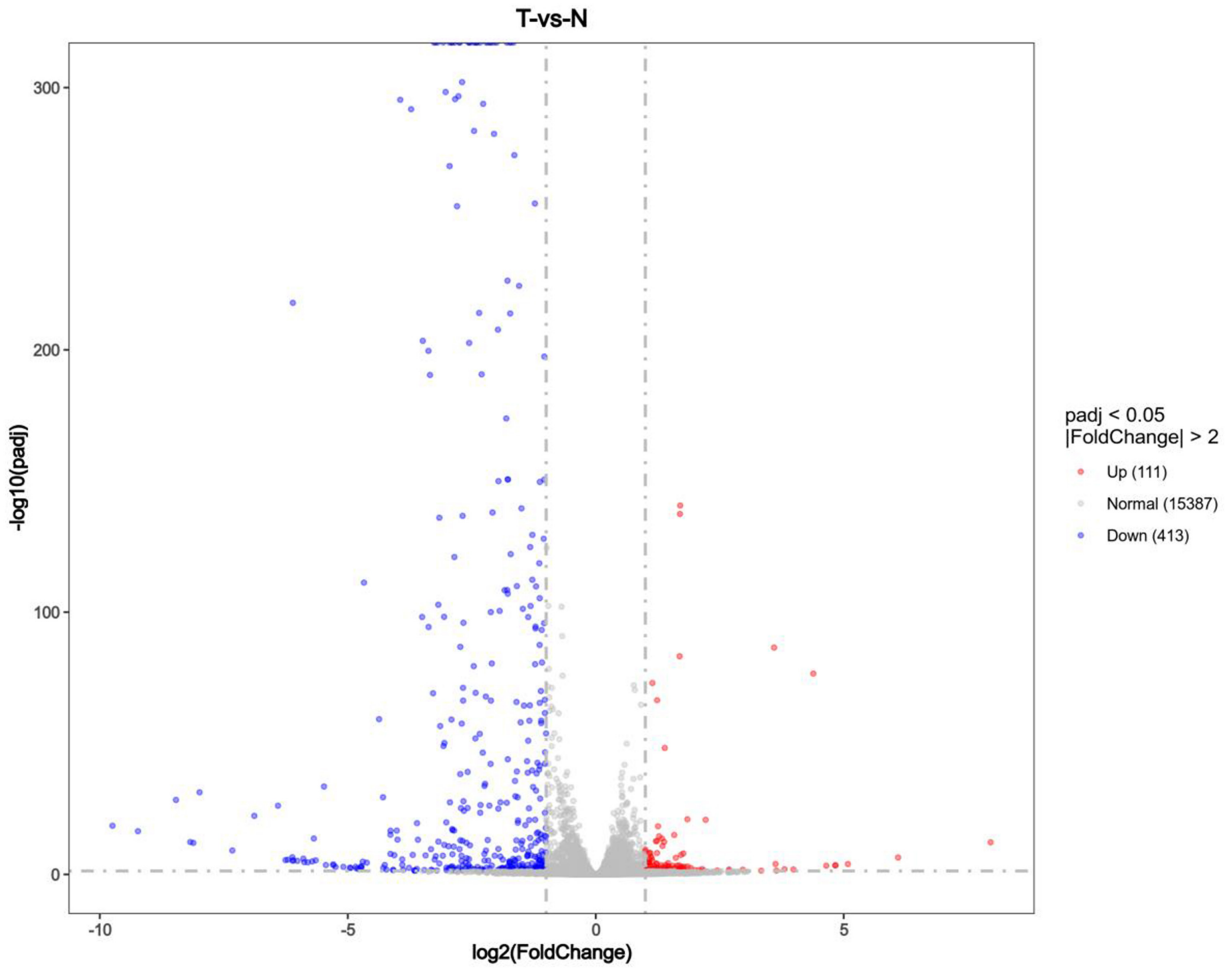
volcano plot of DEGsFigure.

Differentially expressed genes (DEGs) were identified using an adjusted *P*-value (FDR) <0.05 and |log2FC| > 1. Among the 15,911 expressed genes detected, 524 were significantly dysregulated: 111 were up-regulated and 413 were down-regulated in the biochar-treated group. The distribution and statistical significance of these DEGs are summarized in a bar plot and volcano plot (Figures 10, 11, respectively).

**Figure 12:**
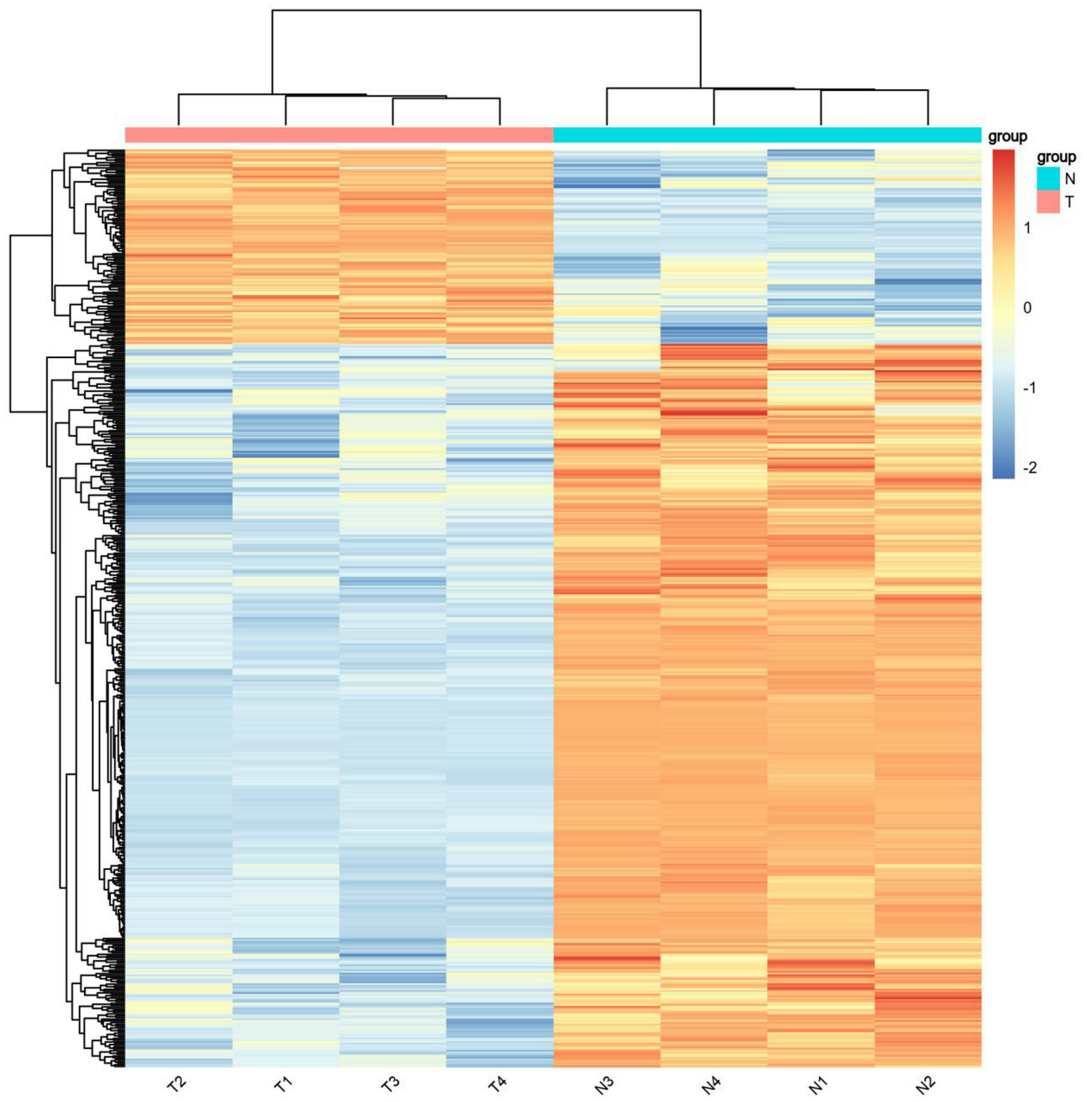
Heatmap plot of DEGs.

**Figure 13:**
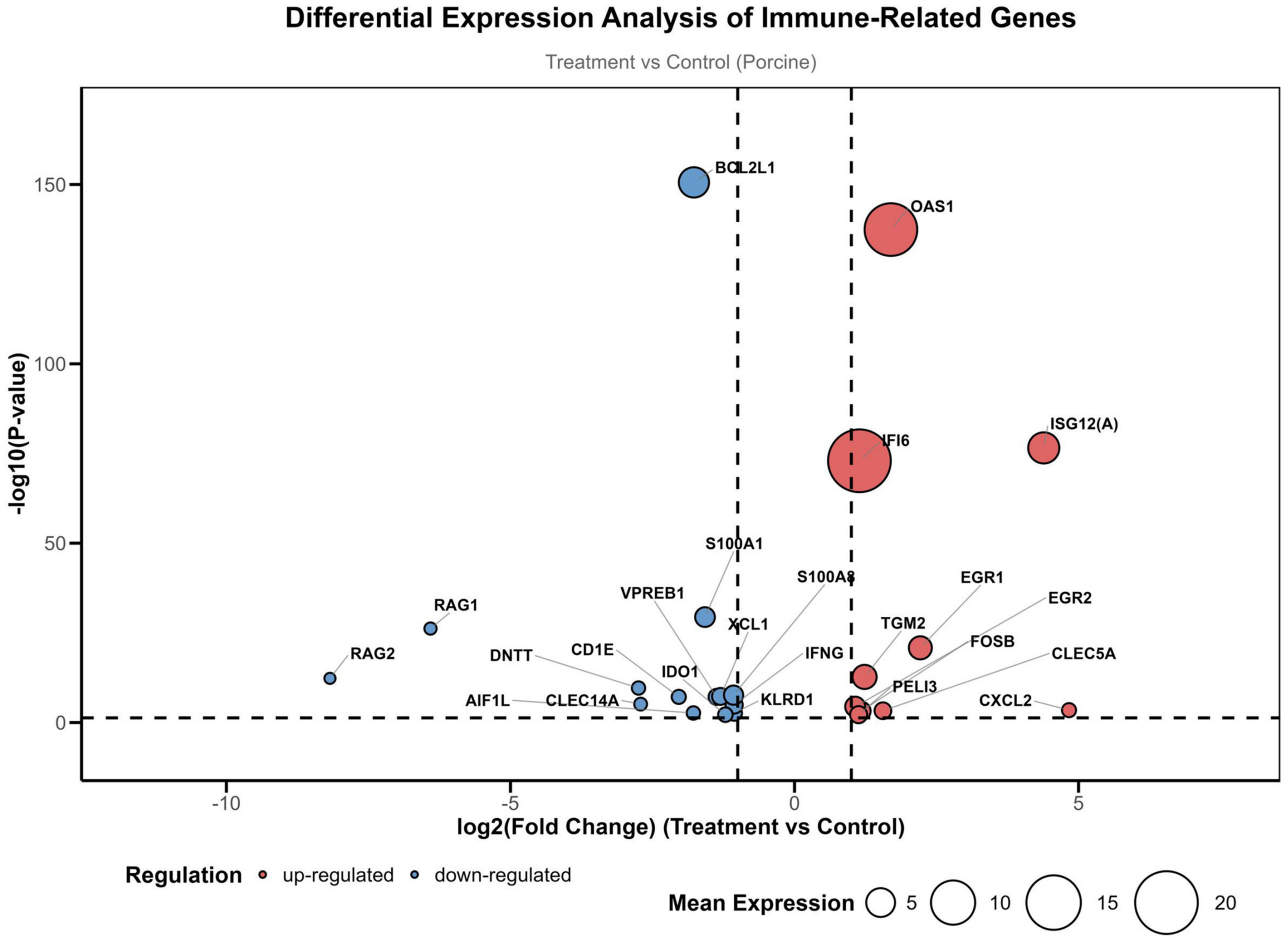
Immune-related DEGs.

To uncover co-expression patterns and infer potential functional modules, genes with similar expression profiles across samples were subjected to hierarchical clustering and visualized in a heatmap (Figure 12). Notably, among the DEGs, several immune-related genes involved in innate immunity, antiviral defense, and lymphocyte development exhibited marked expression changes. These include *OAS1, IFI6, IFNG, CXCL2, IDO1, RAG1, RAG2, CLEC5A, BCL2L1*, and *FOS*. The coordinated dysregulation of these genes suggests that dietary biochar supplementation modulates host immune responses in field-raised pigs. A focused visualization of the key immune-related DEGs is provided in Figure 13.

#### Functional enrichment analysis of differentially expressed gene

**Figure 14:**
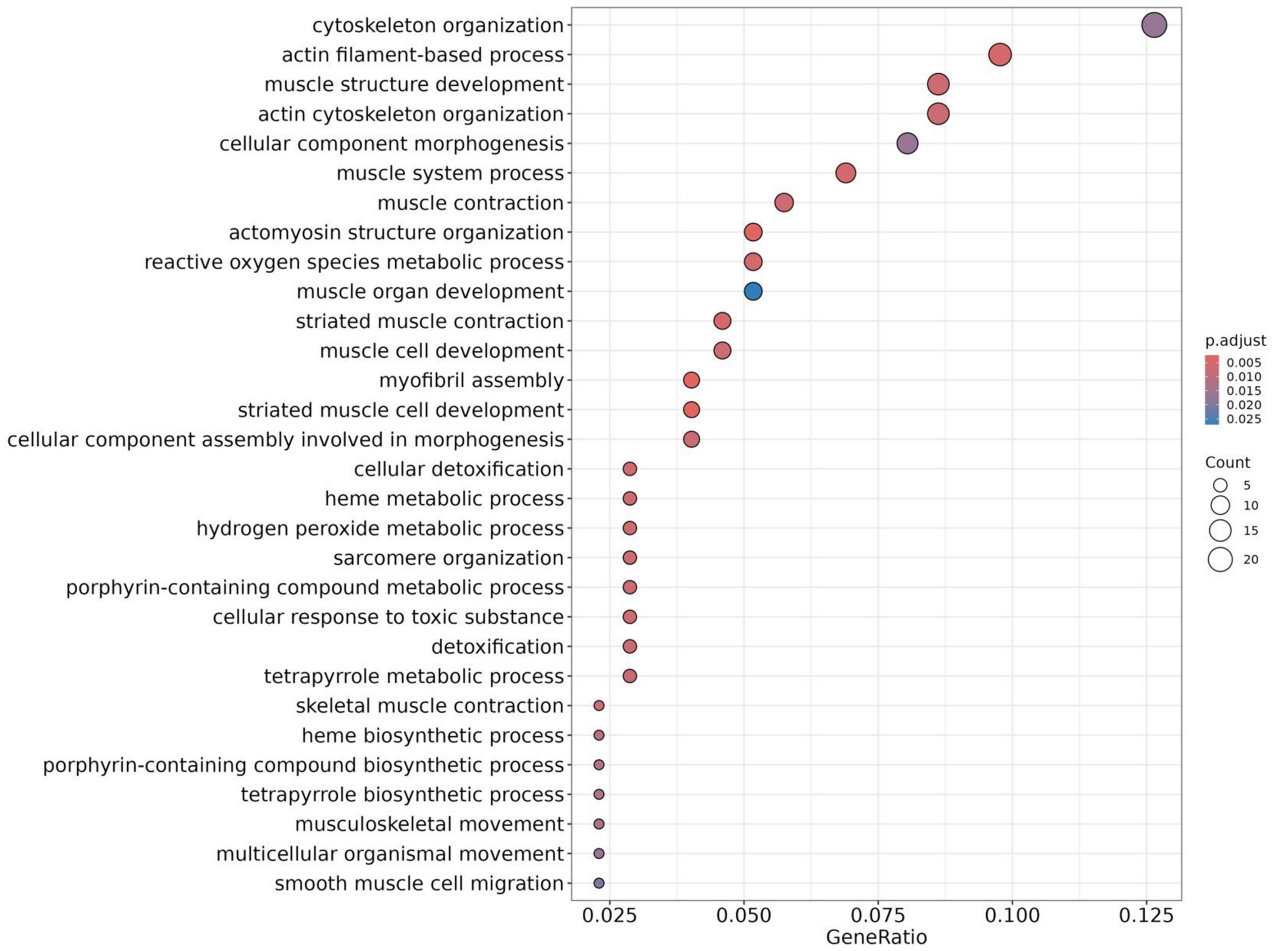
Bubble plot of GO enrichment for DEGs.

**Figure 15:**
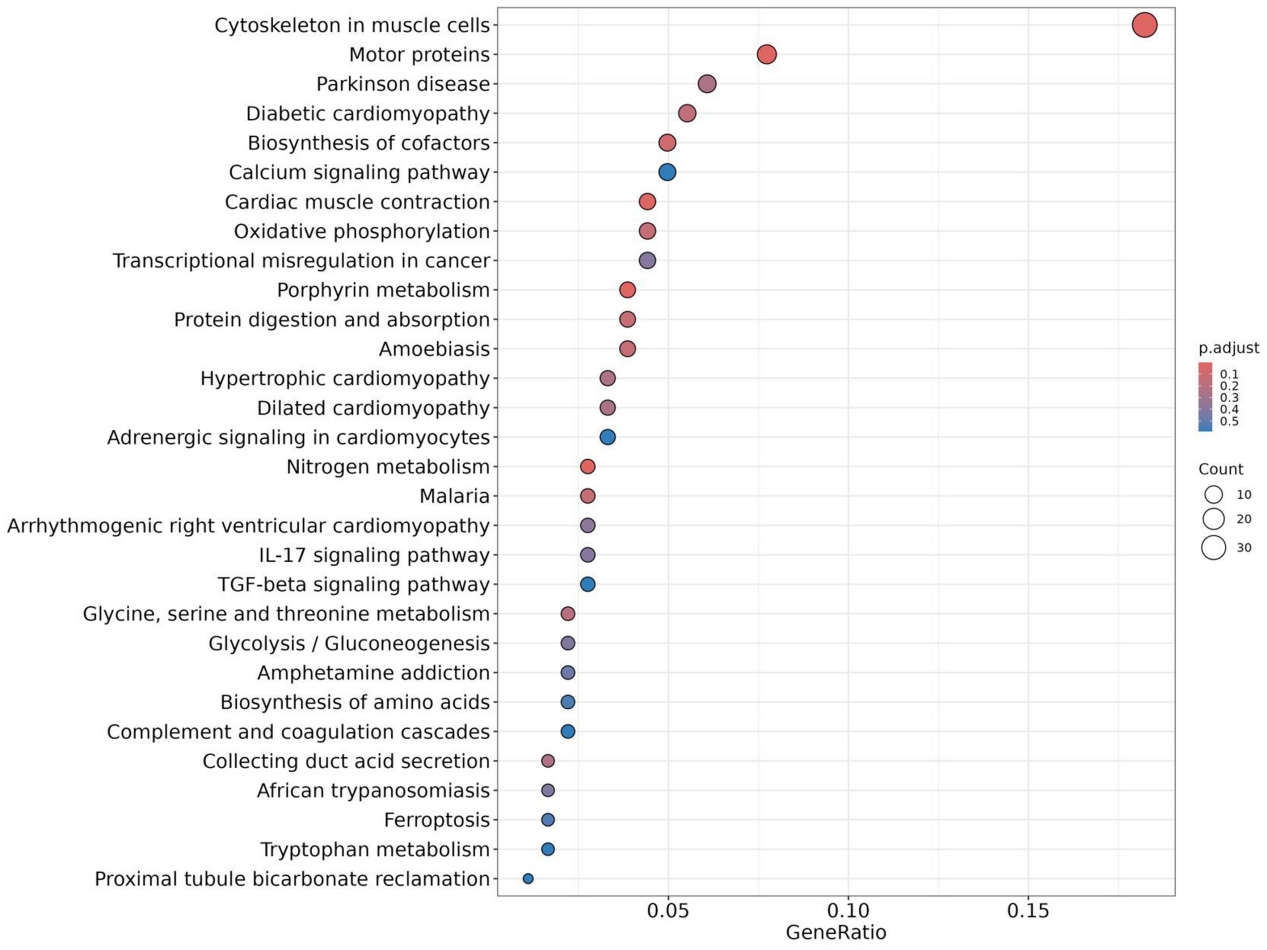
Bubble plot of KEGG enrichment for DEGs.

To elucidate the biological functions and signaling networks associated with the identified DEGs, Gene Ontology (GO) and Kyoto Encyclopedia of Genes and Genomes (KEGG) enrichment analyses were performed. GO classification revealed significant enrichment in biological processes (BP) related to actin filament organization and muscle system regulation. In the cellular component (CC) category, DEGs were predominantly associated with supramolecular complexes, fibers, and polymers. These transcriptional signatures suggest that dietary biochar may promote myofiber development and structural integrity, potentially supporting improved growth performance. Consistent with these findings, KEGG pathway analysis highlighted significant enrichment in pathways governing cytoskeletal dynamics and motor protein activity in muscle tissue. Furthermore, KEGG analysis identified notable enrichment in key immune-modulatory pathways, particularly the IL-17 pathway and TGF-β signaling cascades. In addition, *CXCL-2, FOS*, and *FOSB* genes in IL-17 pathway were significant up-regulated while *FING* gene was down-regulated which means that better immune homeostasis in biochar supplement group. This suggests that biochar supplementation actively modulates transcriptional programs underlying hematological and systemic immune responses. The differentially enriched KEGG immune-related pathways and up-regulated genes were listed in Table 5. The comprehensive GO functional profiles and significantly enriched KEGG pathways are visualized in Figures 14, 15, respectively.

**Table 5:** Differentially enriched KEGG immune-related pathways in transcriptome.

Pathway	*p* value	*p*.adjust	*q* value	Count	Gene ID	Regulation
Complement and coagulation cascades	0.003	0.067	0.063	3	PLAU/PLAUR/SERPINE2	up
IL-17 signaling pathway	0.004	0.072	0.069	3	CXCL2/FOS/FOSB	up
Efferocytosis	0.019	0.271	0.257	3	ALOX15/AXL/P2RY2	up
TNF signaling pathway	0.060	0.414	0.393	2	CXCL2/FOS	up

### Identification of different expressed proteins

#### Quality control

**Figure 16:**
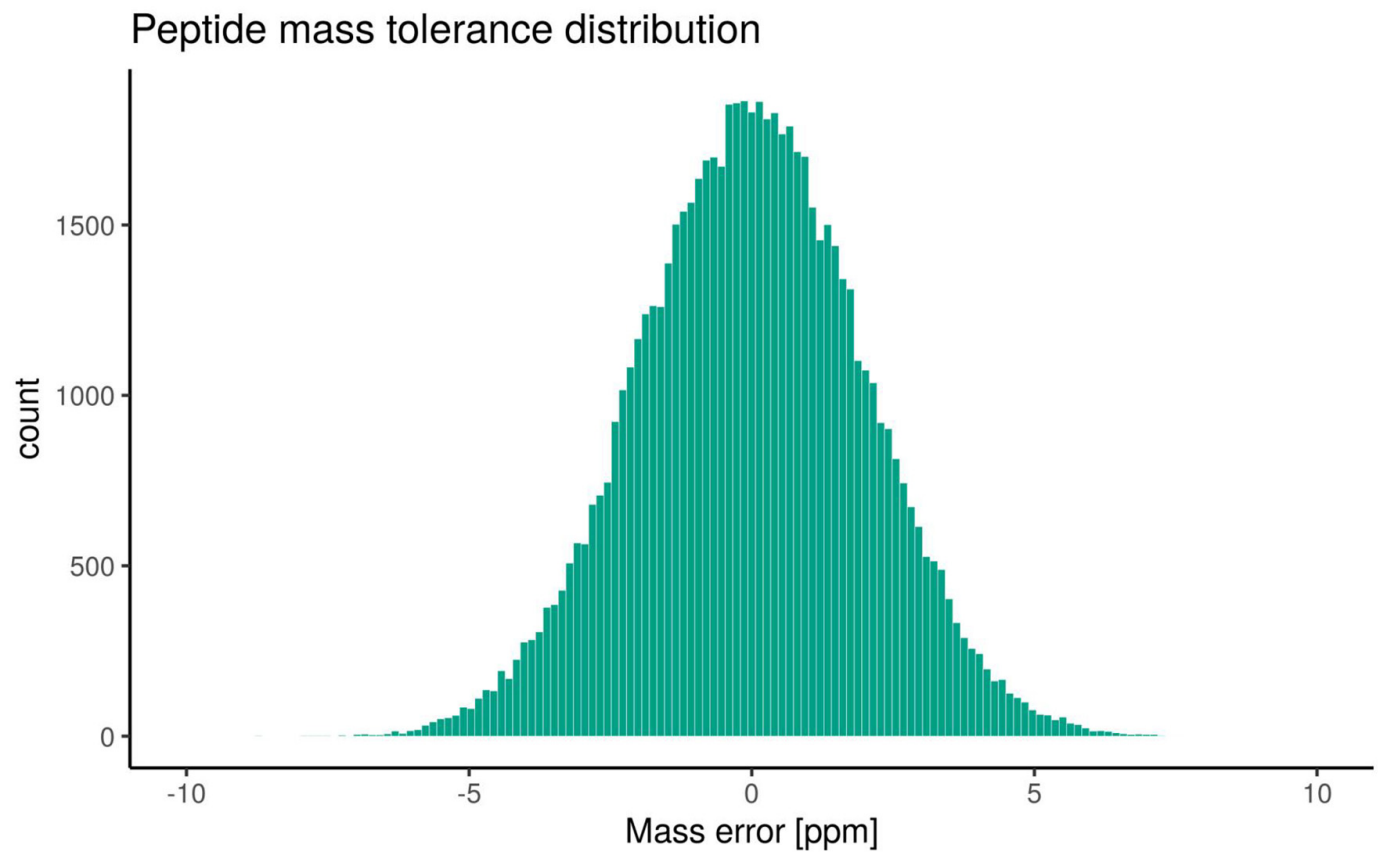
Peptide mass tolerance distribution.

**Figure 17:**
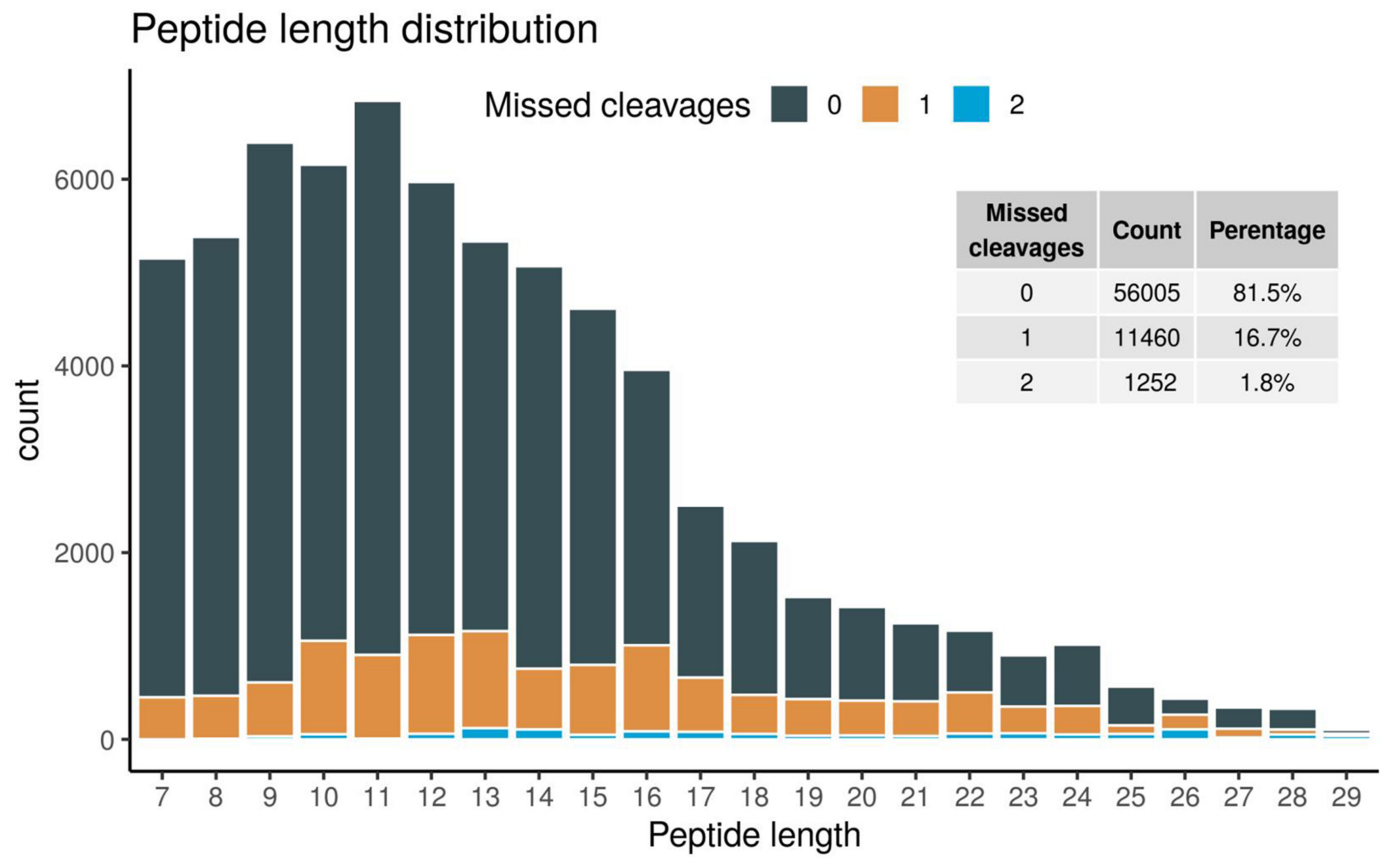
Peptide length distribution.

**Figure 18:**
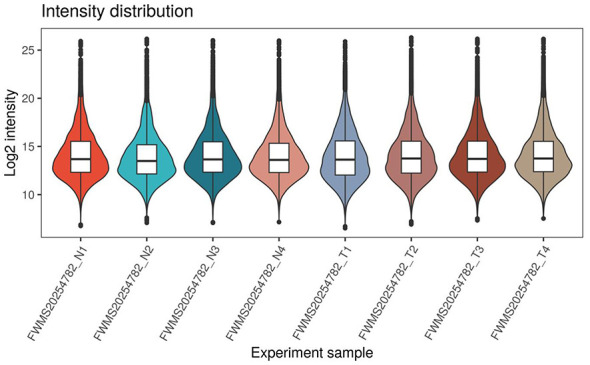
Intensity distribution.

**Figure 19:**
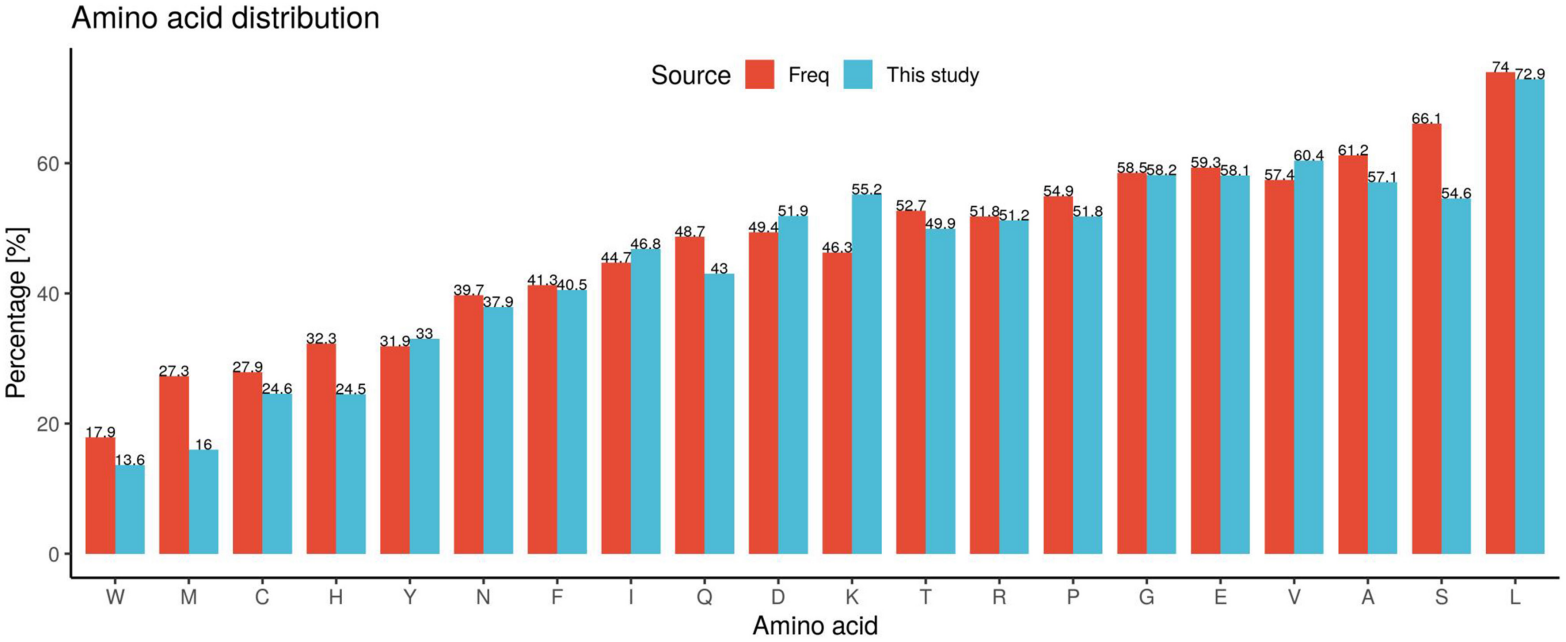
Amino acid distribution.

**Figure 20:**
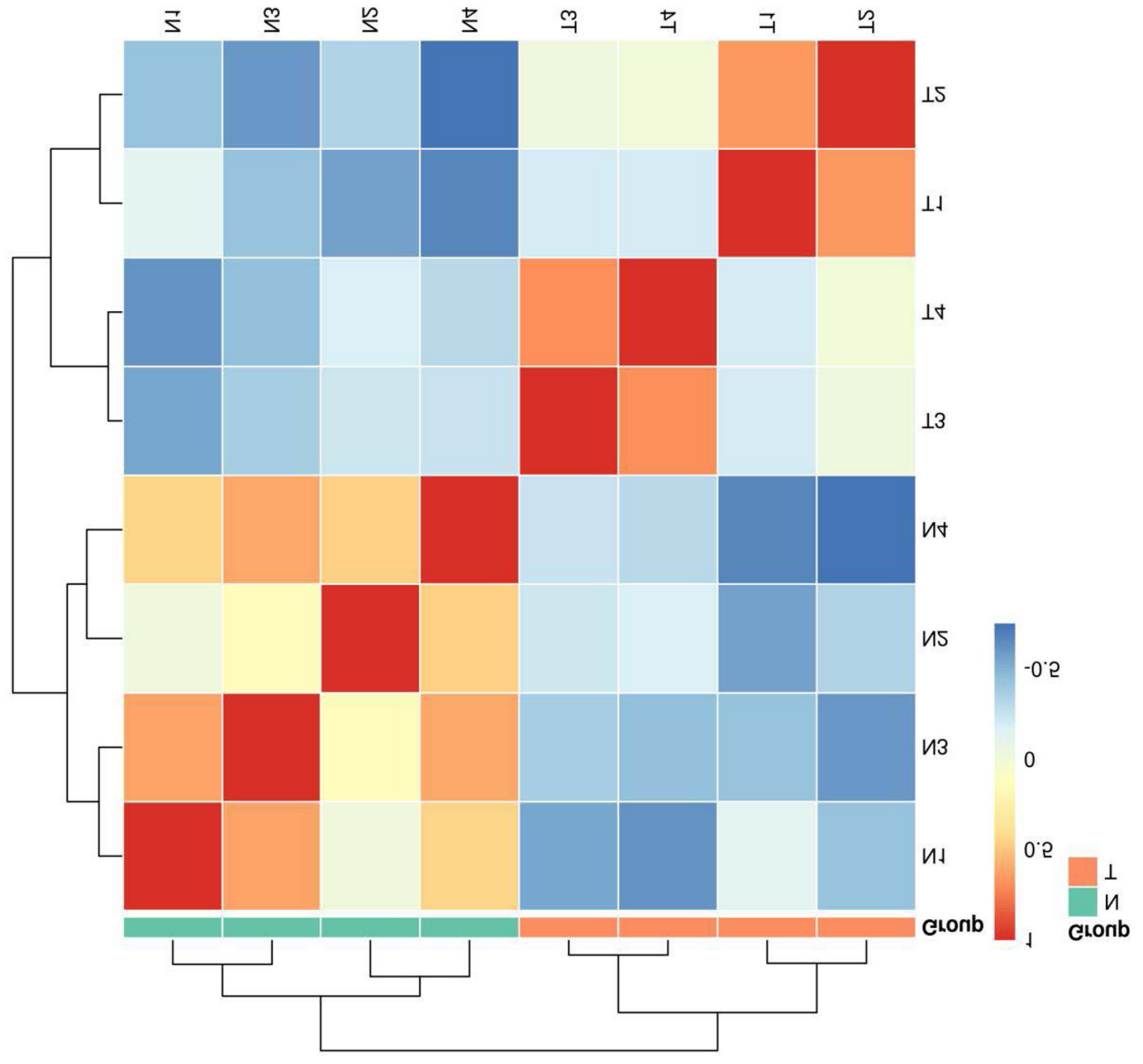
PCC plot.

**Figure 21:**
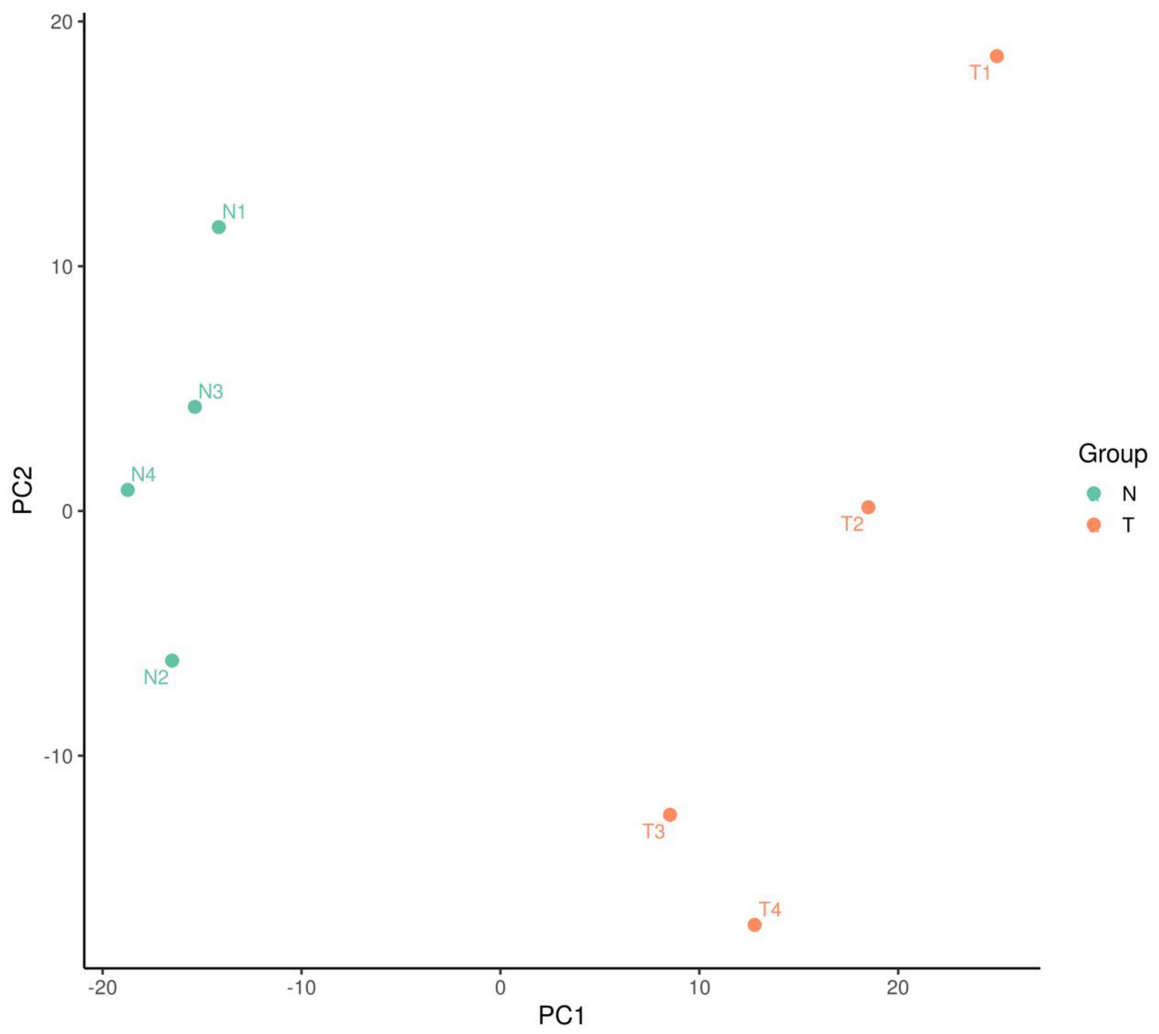
PCA plot.

**Figure 22:**
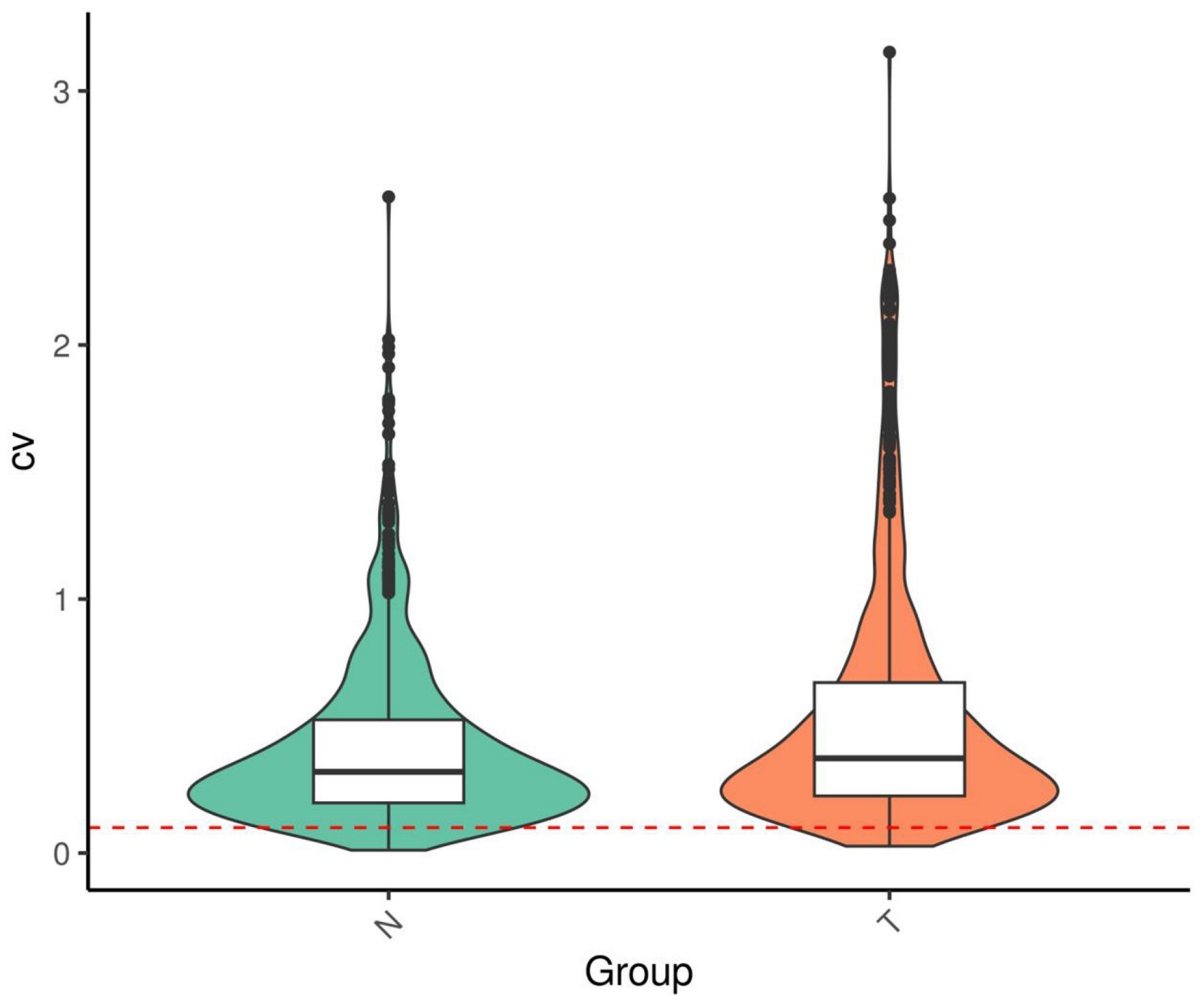
CV plot.

Quality control was assessed based on peptide mass tolerance, peptide length distribution, amino acid composition, and quantitative reproducibility. After database searching, peptide mass error, peptide length, and amino acid frequency were statistically evaluated. The mass deviation between measured and theoretical peptide masses showed a normal distribution, with nearly all values within ±10 ppm (Figure 16). Most identified peptides were 7–20 amino acids in length, and 81.5% contained no missed cleavages, indicating efficient trypsin digestion (Figure 17). Peptide intensity distributions were uniform and met quality control criteria (Figure 18), while amino acid frequencies were consistent with database expectations (Figure 19). These four figures present the core quality control visualizations of a high-resolution mass spectrometry-based quantitative proteomics experiment. They comprehensively demonstrate the accuracy of mass spectrometric detection, the efficiency of enzymatic digestion, the stability of sample replicates, and the reliability of protein identification which could provide a solid and reliable foundation for subsequent protein quantification and differential expression analysis. Quantitative reproducibility and sample consistency were further evaluated using Pearson's correlation coefficient (PCC), principal component analysis (PCA), and relative standard deviation (RSD), as shown in Figures 20–22.

#### Analysis of different expressed proteins

Following LC-MS/MS analysis and DIA quantification, relative protein abundances were obtained from reporter ion intensities after logarithmic transformation, centering, and median normalization. Differentially expressed proteins (DEPs) were defined as proteins with *P* < 0.05 and |log2FC| > 0.379. In total, 892 proteins were identified and quantified. Compared with the control group, 196 proteins were significantly upregulated and 186 were significantly downregulated, while the remaining 510 proteins showed no significant changes between the two groups.

**Figure 23:**
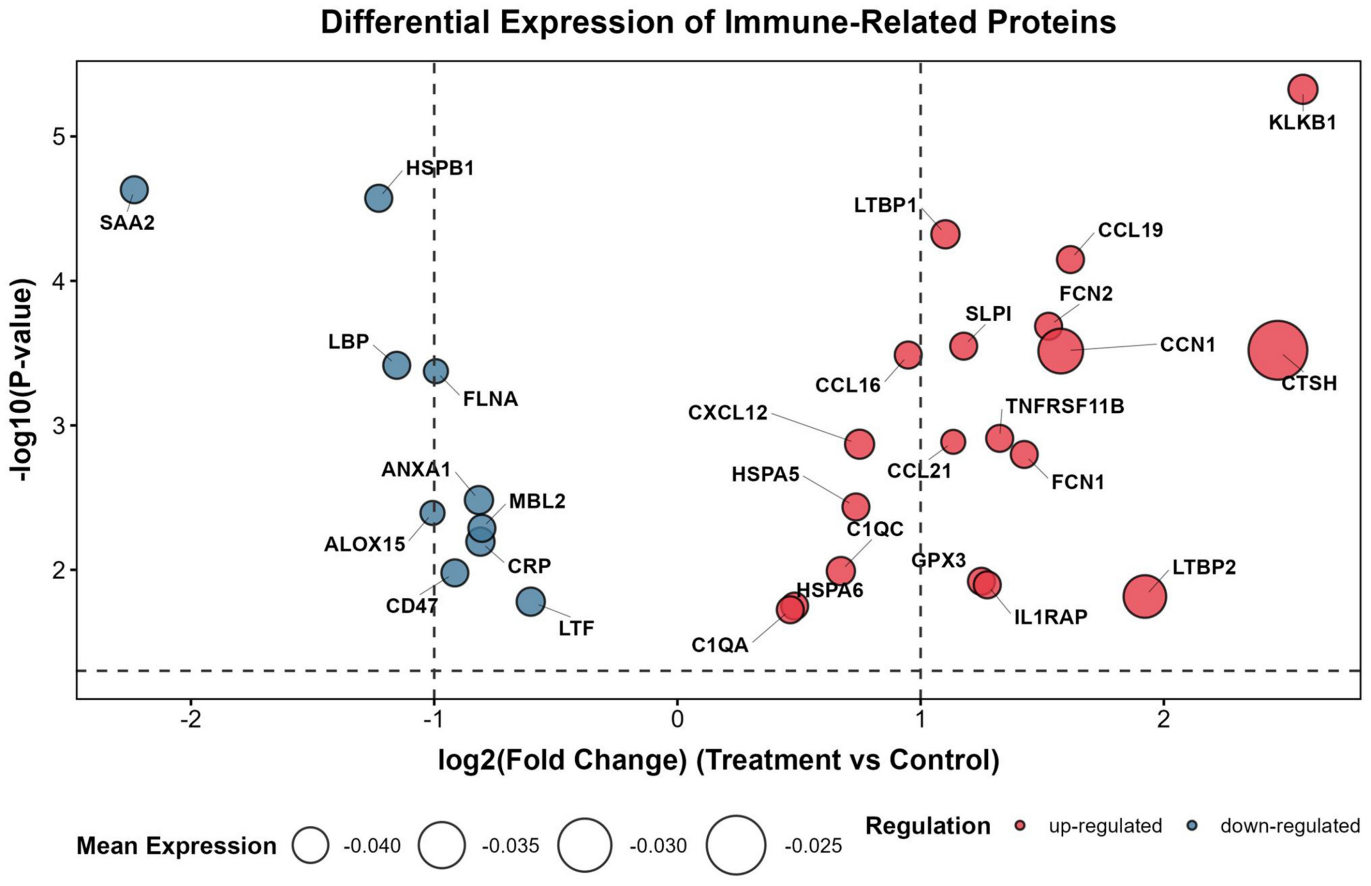
Immune-related DEPs.

**Figure 24:**
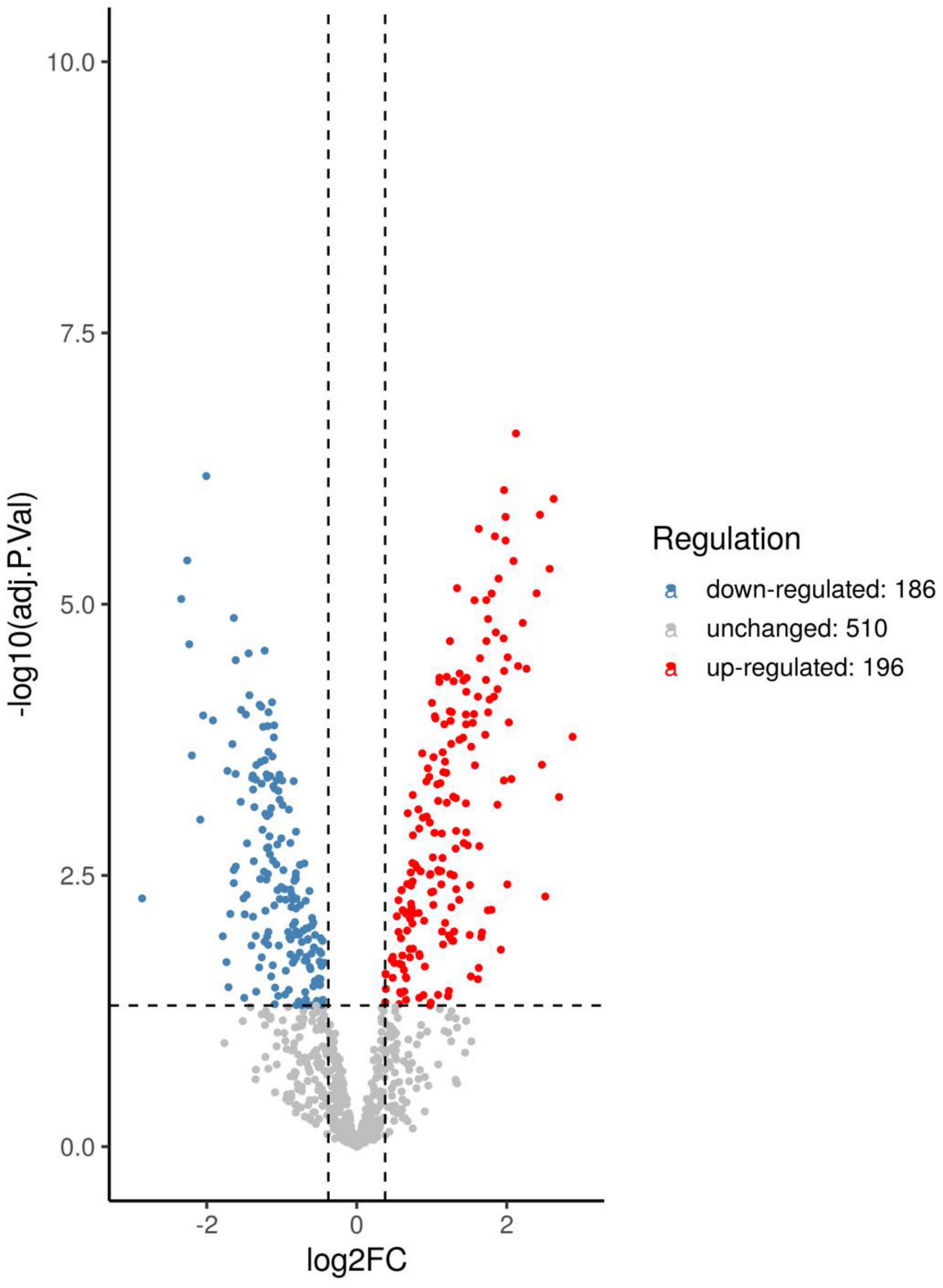
DEPs volcano of T-vs-N.

**Figure 25:**
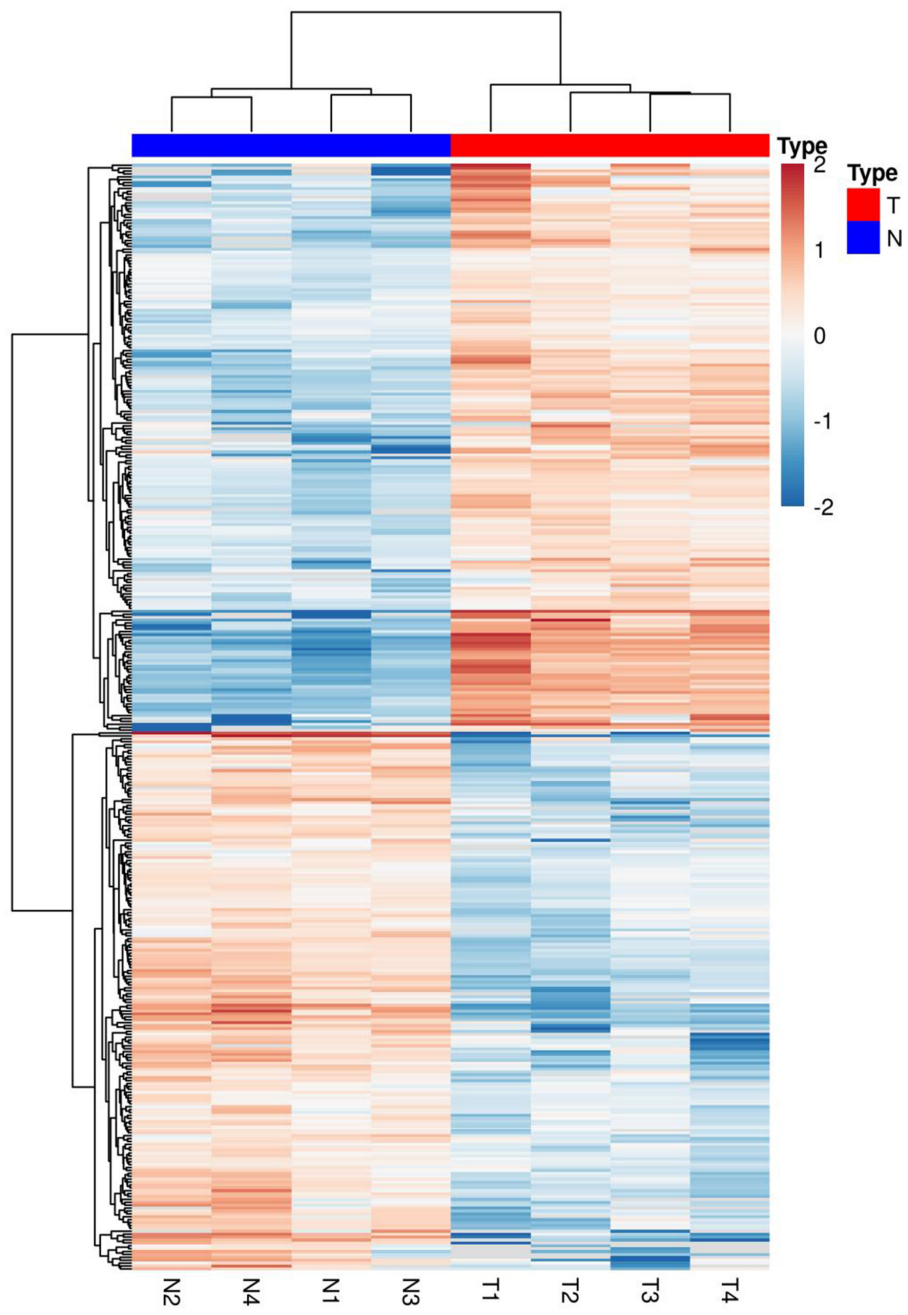
DEPs heatmap of T-vs-N.

Among the upregulated proteins, several immune-related proteins exhibited increased expression, including Kallikrein B1 (*KLKB1*), pro-cathepsin H (*CTSH*), SLPI_PIG Antileukoproteinase (*SLPI*), A0A286ZZX3_PIG Metalloproteinase inhibitor 3 (*TIMP3*) and an Ig-like domain-containing protein (*A0A5G2QXT5_PIG*). In contrast, several immune-related proteins were downregulated, such as Serum amyloid A-2 protein (*SAA2*), CRP_PIG C-reactive protein (*CRP*), Proteoglycan 3 (*LOC100739611*), MYH1_PIG Myosin-1 (*MYH1*), S10AC_PIG Protein S100-A12 (*S100A12*) and another Ig-like domain-containing protein (*A0A8W4FEL9_PIG*). The essential differentially expressed immune-related proteins is shown in Figure 23. In addition, several chemokines, including *CCL16, CCL19, CCL21*, and *CXCL12*, showed increased expression in the experimental group. Ficolins such as *FCN1* and *FCN2* were also more abundant compared with the control group. These differentially expressed inflammation-related proteins were listed in Table 6. Conversely, *CRP* and *SAA* were downregulated in the experimental group. The differential expression patterns of all 892 detected proteins are visualized in Figures 24, 25.

**Table 6:** Differentially expressed inflammation-related proteins.

Gene name	EG mean	CG mean	log2FC	*P*.Value	adj.*P*.Val	Regulation
FCN2	0.722 ± 0.287	−0.805 ± 0.395	1.526	*P* < 0.001	*P* < 0.001	up–regulated
CCL16	0.433 ± 0.172	−0.516 ± 0.164	0.949	*P* < 0.001	*P* < 0.001	up–regulated
CCL19	0.767 ± 0.289	−0.850 ± 0.317	1.616	*P* < 0.001	*P* < 0.001	up–regulated
CXCL12	0.333 ± 0.130	−0.416 ± 0.186	0.749	*P* < 0.001	*P* < 0.001	up–regulated
A0A5G2QXT5_PIG	1.398 ± 0.239	−1.481 ± 0.971	2.878	*P* < 0.001	*P* < 0.001	up–regulated
KLKB1	1.245 ± 0.404	−1.328 ± 0.287	2.572	*P* < 0.001	*P* < 0.001	up–regulated
LOC100739611	−1.212 ± 0.360	1.129 ± 0.330	−2.34	*P* < 0.001	*P* < 0.001	down–regulated
CRP	−0.447 ± 0.419	0.364 ± 0.113	−0.81	*P* < 0.001	*P* < 0.001	down–regulated
SAA2	−1.158 ± 0.508	1.075 ± 0.191	−2.233	*P* < 0.001	*P* < 0.001	down–regulated
SLPI	0.547 ± 0.323	−0.631 ± 0.144	1.177	*P* < 0.001	*P* < 0.001	up–regulated
S100A12	−0.303 ± 0.334	0.219 ± 0.138	−0.522	*P* < 0.05	*P* < 0.05	down–regulated
FCN2	0.375 ± 0.226	−0.459 ± 0.159	0.833	*P* < 0.001	*P* < 0.001	up–regulated
FCN1	0.671 ± 0.389	−0.755 ± 0.538	1.426	*P* < 0.001	*P* < 0.001	up–regulated
CCL21	0.525 ± 0.288	−0.609 ± 0.378	1.134	*P* < 0.001	*P* < 0.001	up–regulated
MYH1	−1.000 ± 0.315	0.917 ± 0.501	−1.917	*P* < 0.001	*P* < 0.001	down–regulated
TIMP3	1.020 ± 0.117	−1.104 ± 0.095	2.123	*P* < 0.001	*P* < 0.001	up–regulated

EG, Experiment Group (biochar treatment); CG, Control Group.

#### Functional classification and enrichment analysis of differentially expressed proteins

The list of differentially expressed proteins (DEPs) was compiled based on relative quantities, including relevant information such as gene name, log2FC, *P* value, and regulation direction. Using all DEPs, Gene Ontology (GO) annotation was performed according to alignment results with the UniProt-GOA database, covering biological processes, cellular components, and molecular function.

**Figure 26:**
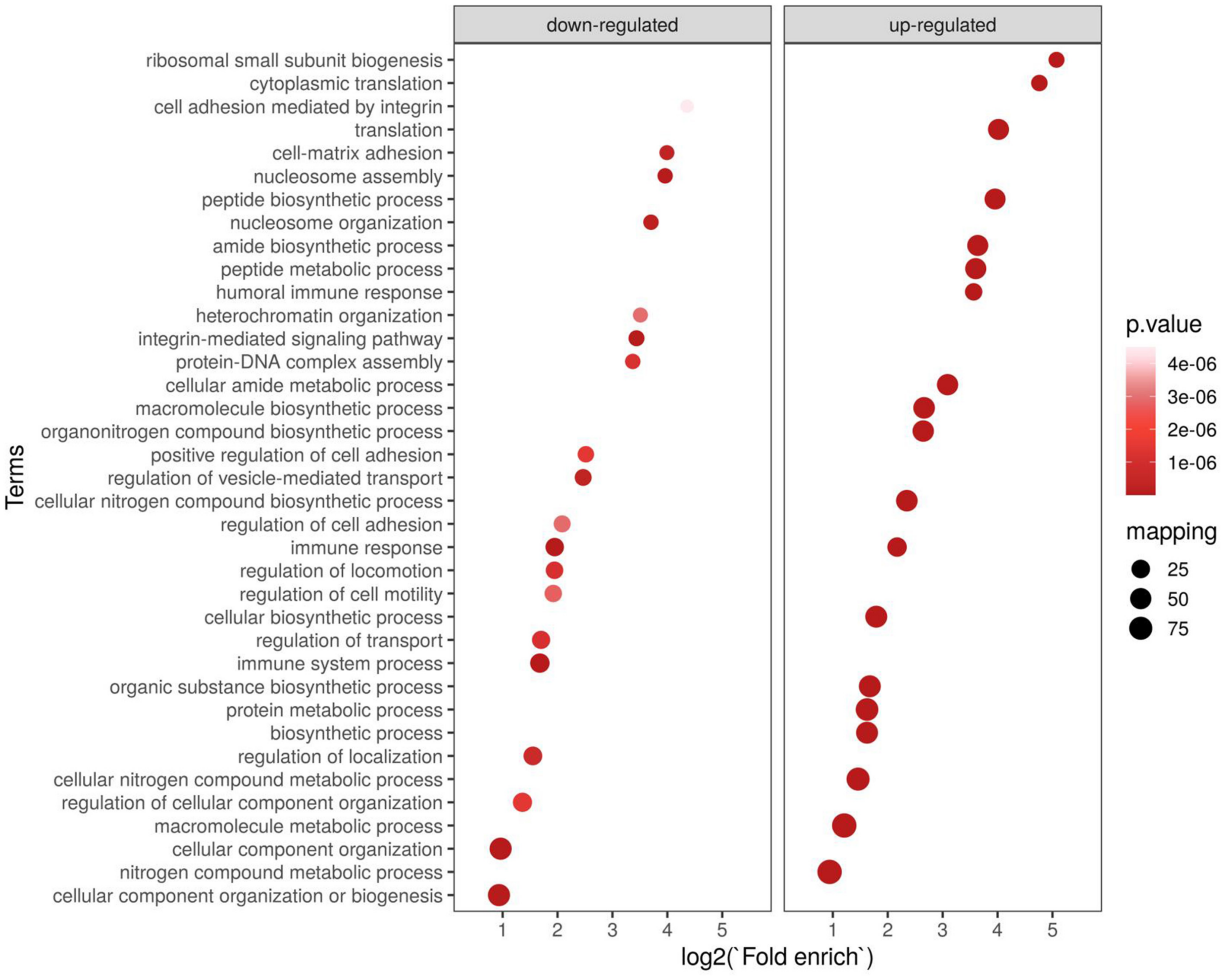
Biological process enrichment of T-vs-N.

**Figure 27:**
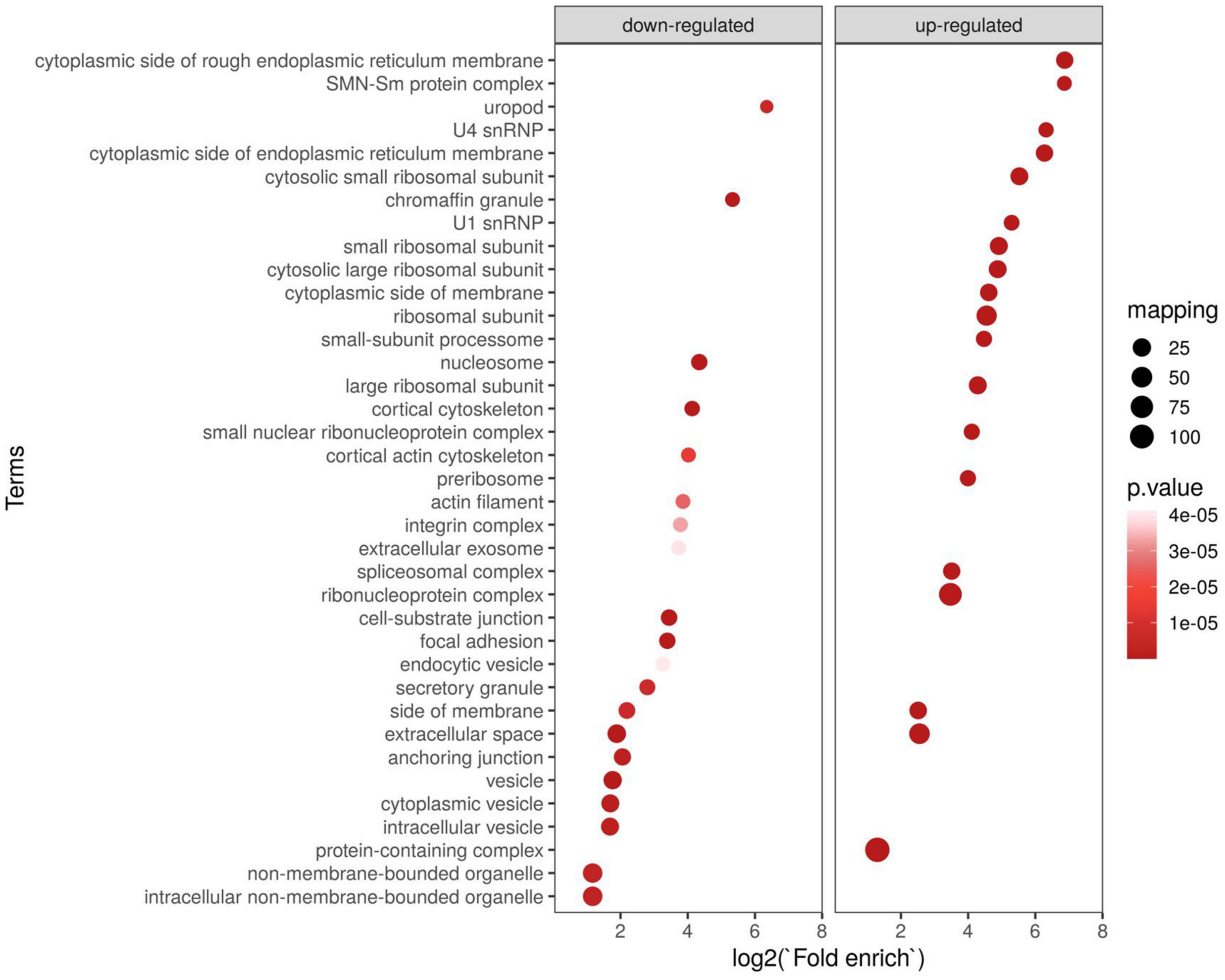
Cellular component enrichment of T-vs-N.

**Figure 28:**
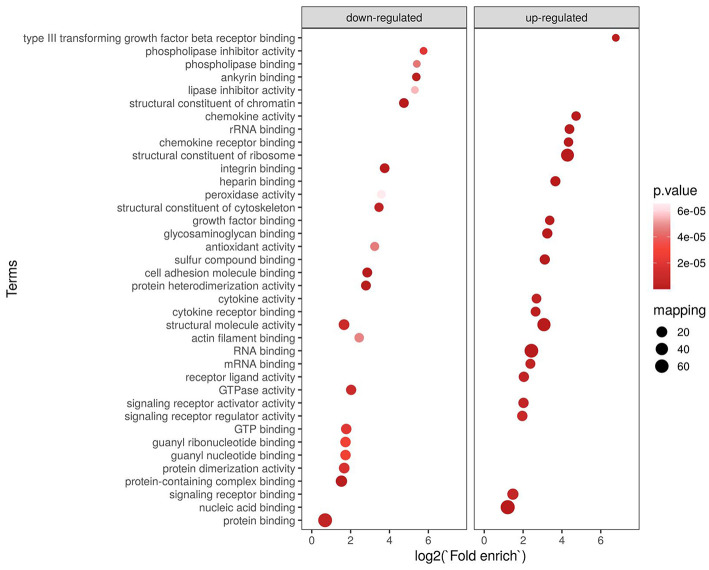
Molecular function enrichment of T-vs-N.

Based on log2 fold enrichment, the top upregulated biological processes included ribosomal small subunit biogenesis, integrin-mediated cell adhesion, peptide biosynthetic process, and humoral immune response. Downregulated processes were mainly nucleosome assembly and organization (Figure 26). For cellular components, upregulated proteins were enriched in the cytoplasmic side of the endoplasmic reticulum membrane, SMN-Sm protein complex, small ribosomal subunit, and cytoplasmic side of the membrane, suggesting enhanced protein biosynthesis and secretion in the experimental group (Figure 27). Regarding molecular function, downregulated proteins were involved in phospholipase inhibitor activity, lipase inhibitor activity, and phospholipase binding, while upregulated proteins with the largest fold changes were associated with chemokine activity, chemokine receptor binding, and structural constituent of the ribosome, indicating enhanced immunity in the experimental group (Figure 28).

**Figure 29:**
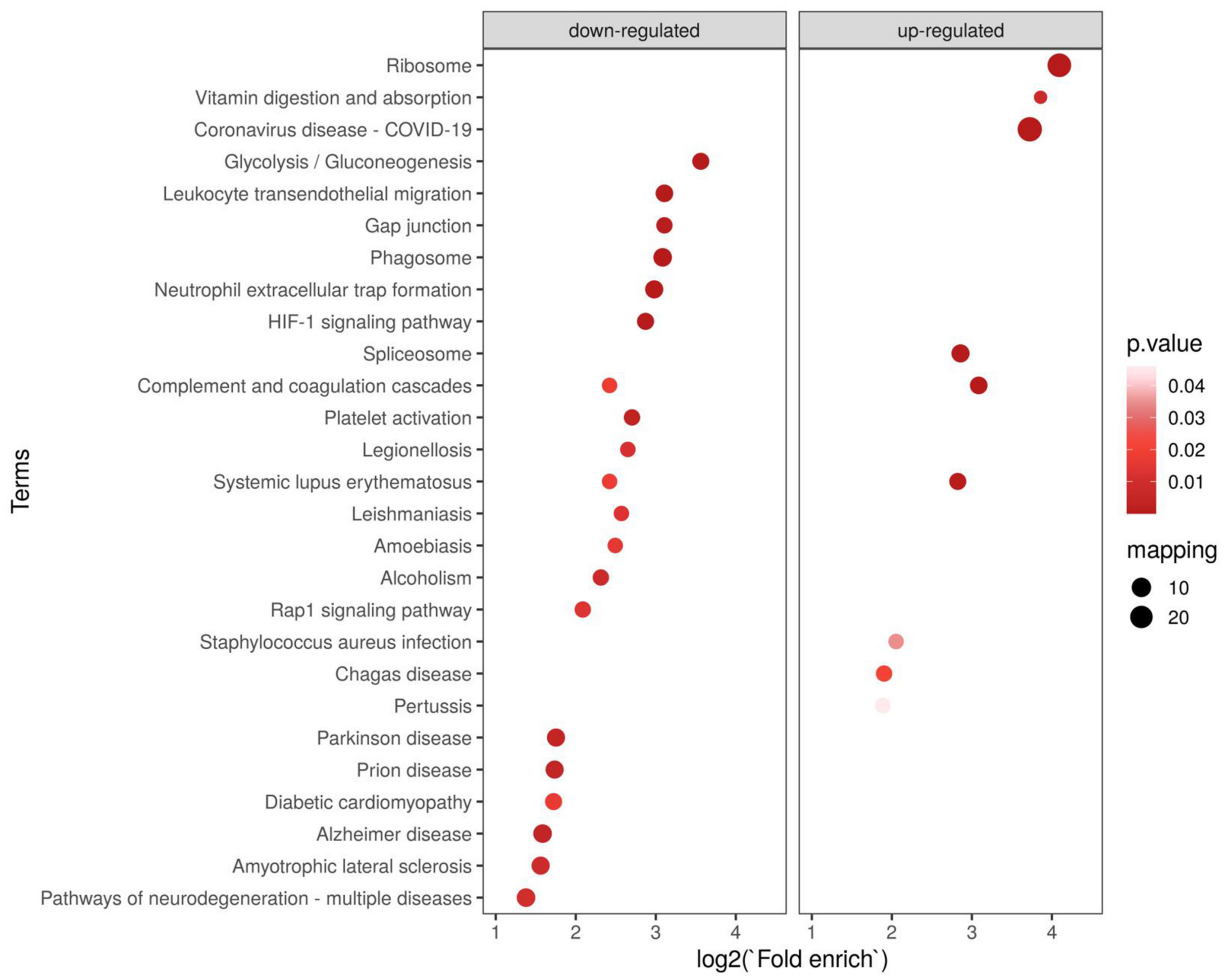
KEGG enrichment of T-vs-N.

**Figure 30:**
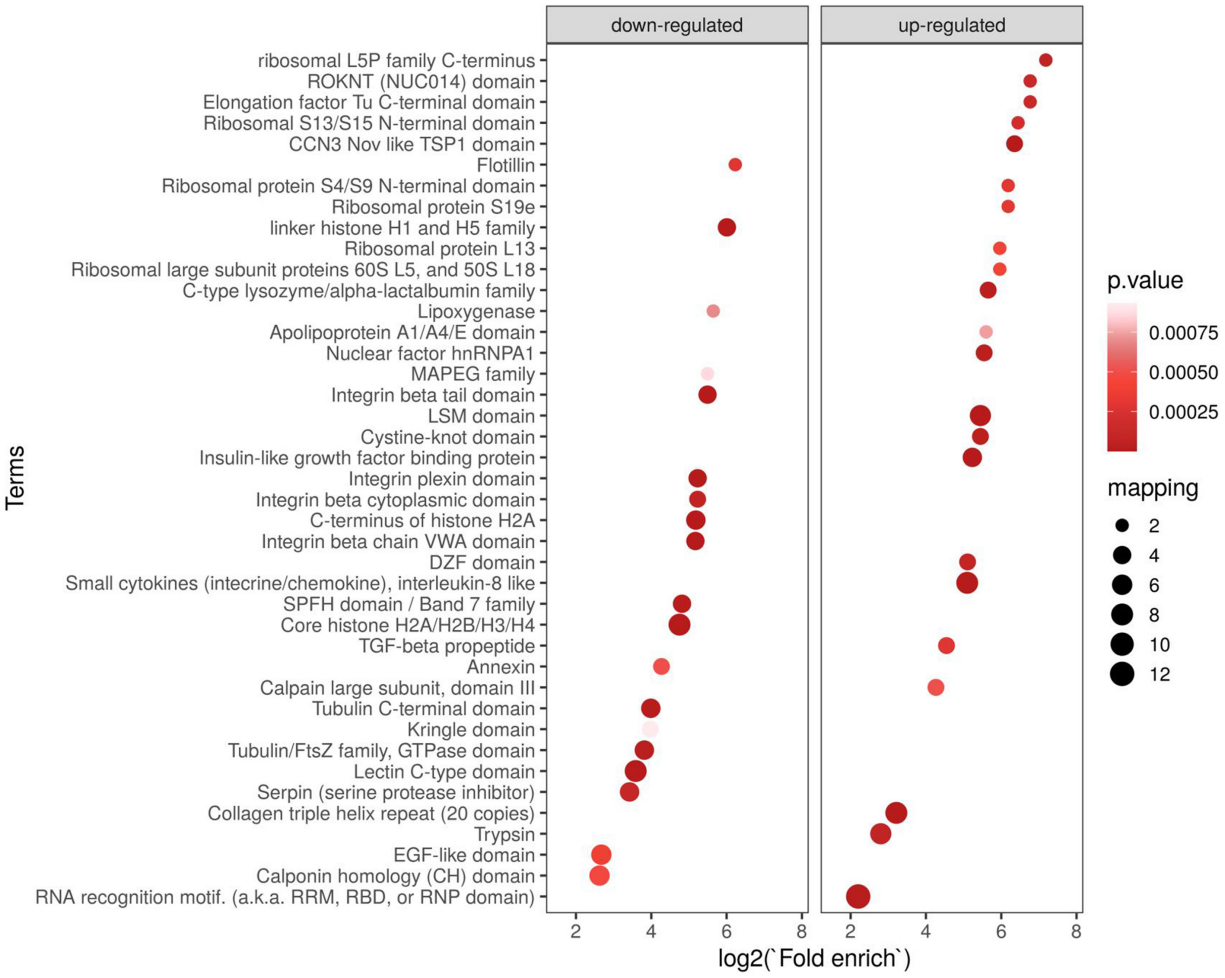
Pfam enrichment of T-vs-N.

**Figure 31:**
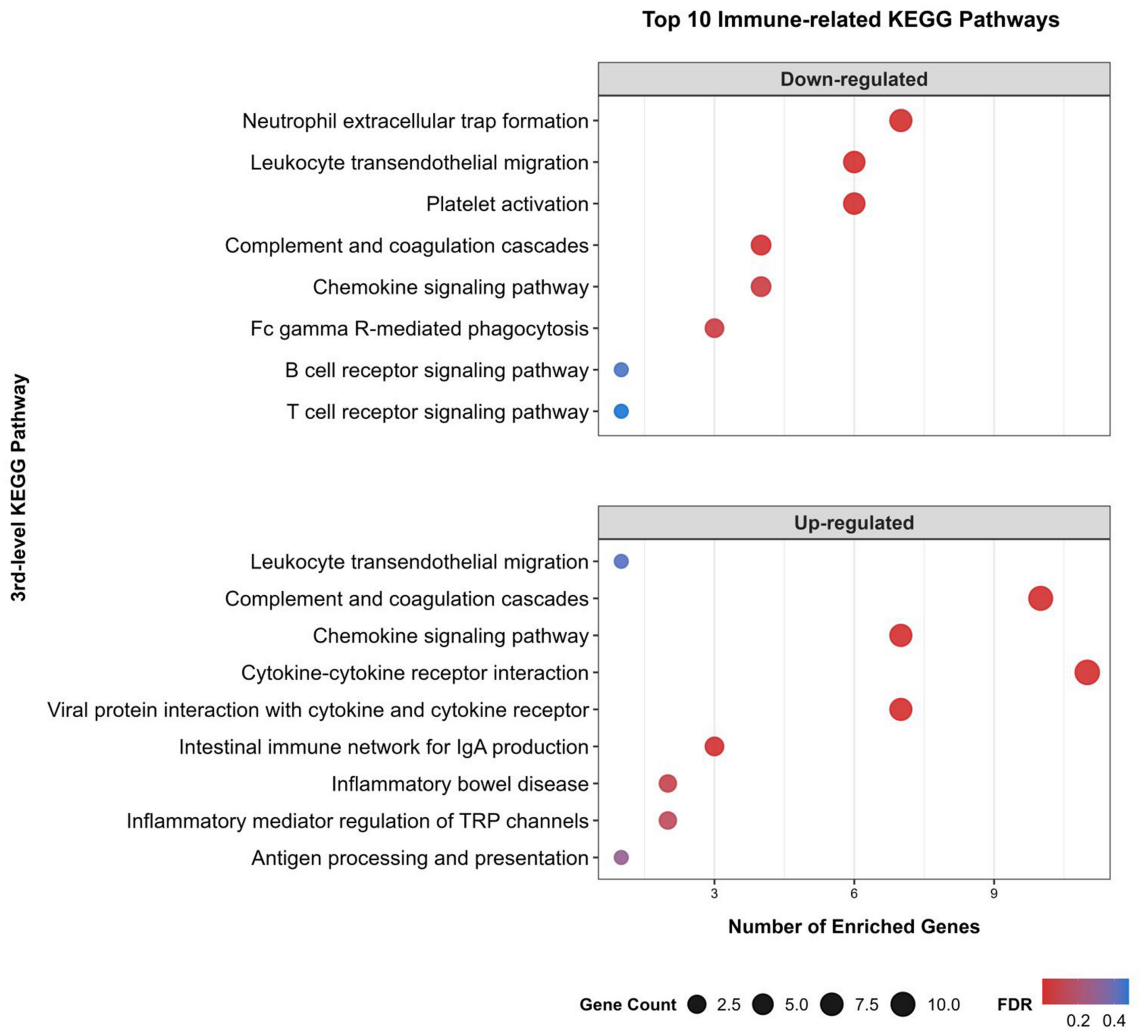
Immune-related KEGG enrichment of T-vs-N.

To further explore the functional impact of DEPs, KEGG analysis was performed. Downregulated pathways included glycolysis/gluconeogenesis, leukocyte transendothelial migration, phagosome, and neutrophil extracellular trap formation. Upregulated pathways included ribosome, vitamin digestion and absorption, and Coronavirus disease-COVID-19 (Figure 29). Protein domain enrichment analysis showed that the ribosomal L5P family C-terminus, elongation factor Tu C-terminal domain, and CCN3 Nov-like TSP1 domain were more abundant in the experimental group (Figure 30). To assess whether biochar addition enhanced immunity, immune-related KEGG analysis was conducted, with upregulated and downregulated pathways shown in Figure 31. The complement and coagulation cascades and cytokine-cytokine receptor interaction were the most significantly altered pathways in the experimental group.

### Integrated transcriptomic and proteomic analysis

**Figure 32:**
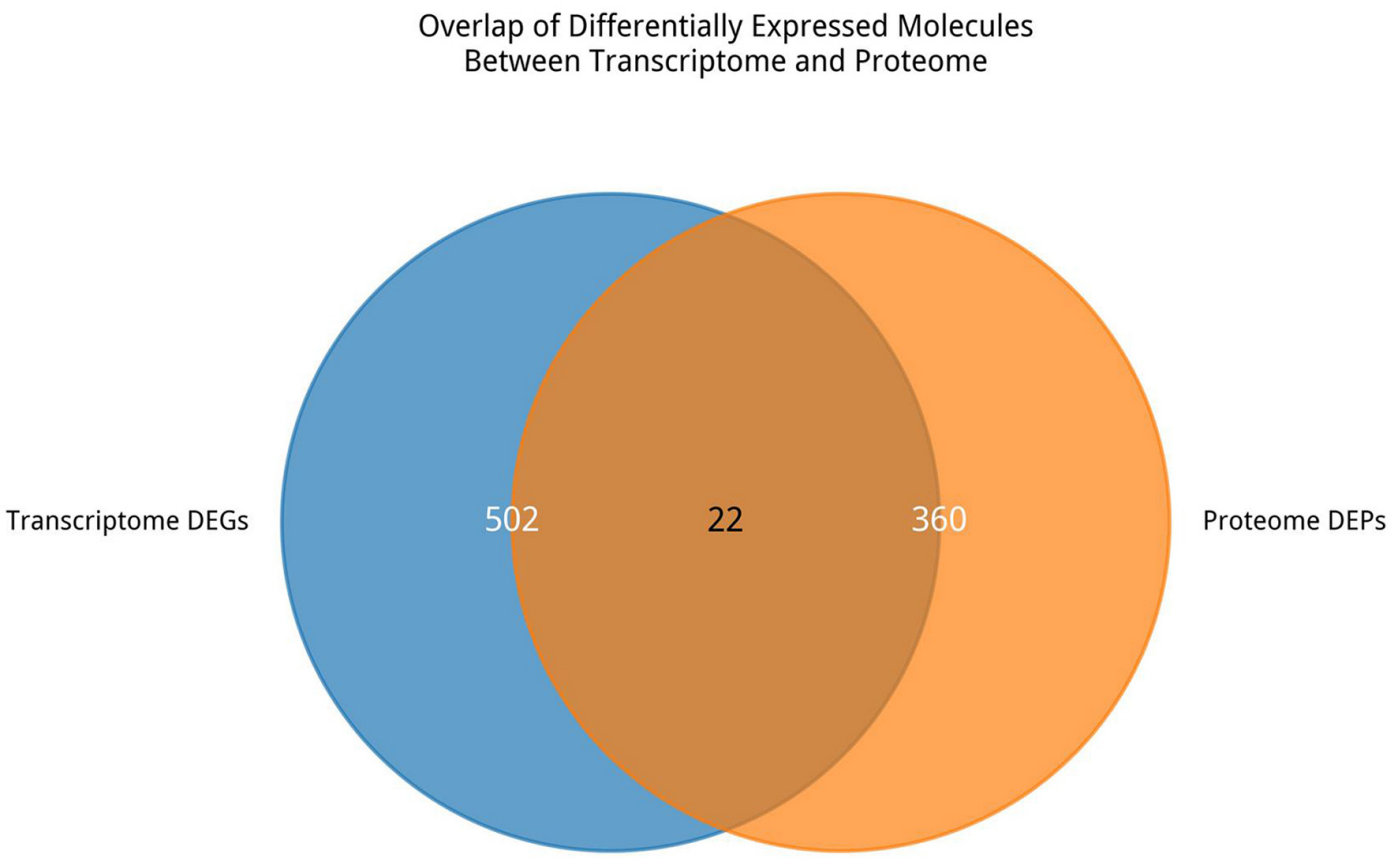
Venn diagram of integrated transcriptomic and proteomic analyses.

An integrated transcriptomic and proteomic analysis was conducted to investigate molecular changes at both RNA and protein levels. Venn analysis identified 502 DEGs unique to the transcriptome, 360 DEPs unique to the proteome, and 22 overlapping molecules that were differentially expressed in both datasets (Figure 32).

**Figure 33:**
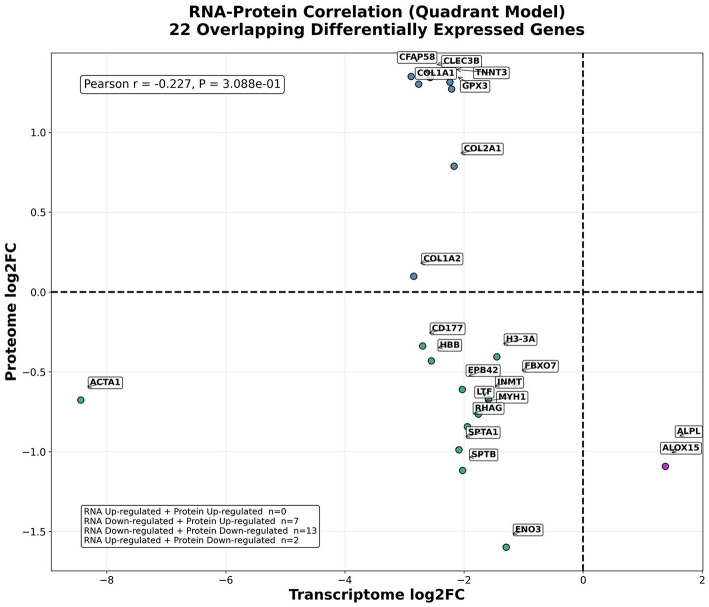
integrated RNA-protein correlation.

**Figure 34:**
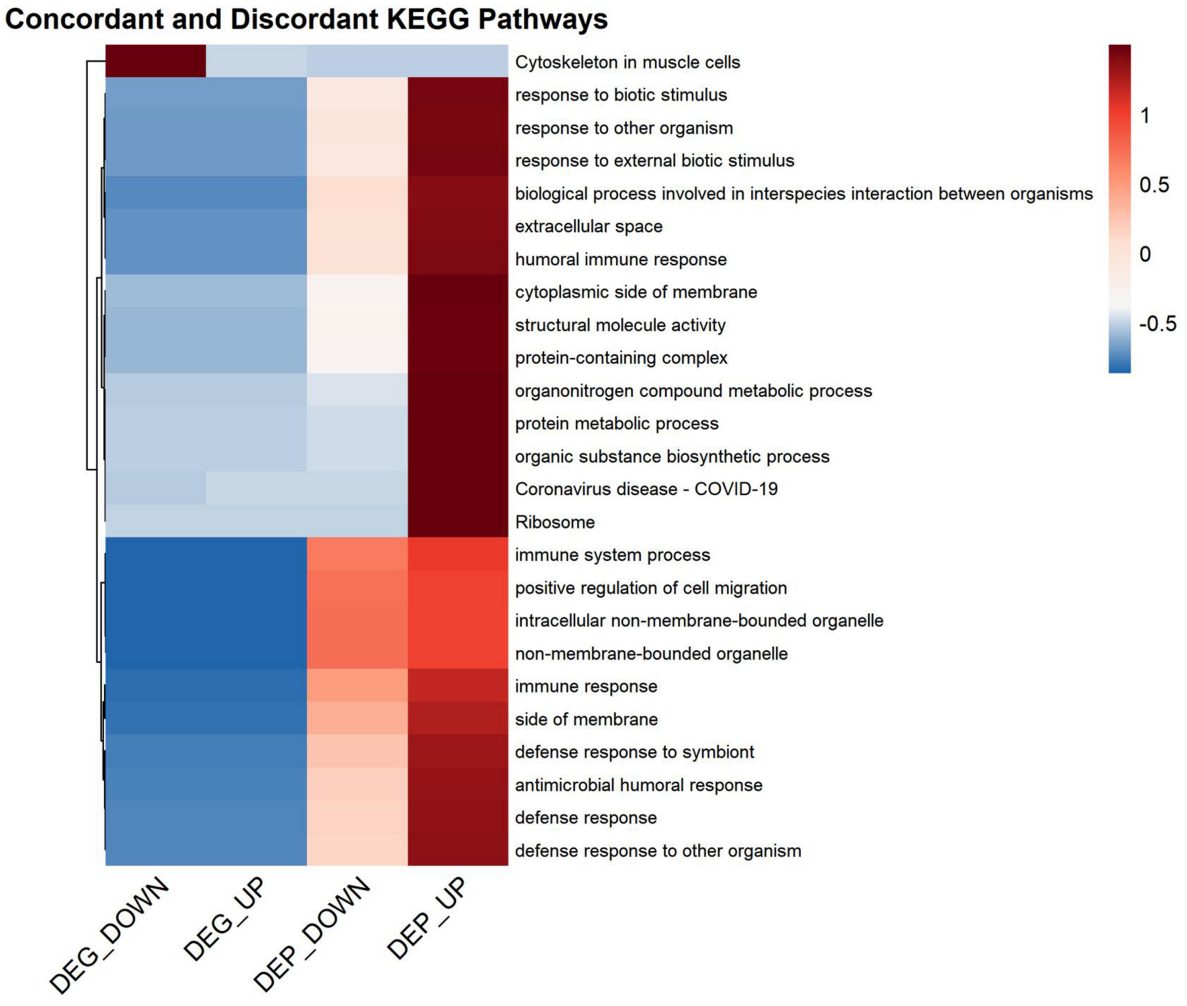
Concordant and discordant KEGG pathways.

To assess the relationship between transcript and protein expression, a quadrant analysis was performed for the 22 overlapping molecules (Table 7). The overall correlation between mRNA and protein abundance was weak and not significant (R = −0.0227, *p* > 0.05). Among the overlapping molecules, 13 were down-regulated at both RNA and protein levels (Down-Down), 2 were up-regulated at the RNA level but down-regulated at the protein level (RNA↑Protein↓), and 7 were down-regulated at the RNA level but up-regulated at the protein level (RNA↓Protein↑), whereas no molecules were simultaneously up-regulated at both levels (Up-Up) (Figure 33). To investigate concordant and discordant regulation between transcriptome and proteome, KEGG enrichment analysis was performed on DEGs and DEPs. Immune-and inflammation-related pathways, including immune response, defense response, and antimicrobial humoral response, were significantly enriched in upregulated DEPs (DEP_UP), whereas these pathways were predominantly downregulated in DEGs (DEG_DOWN) (Figure 34).

**Table 7:** Quadrant classification of the 22 overlapping genes.

Gene Name	Transcriptome log2FC	Proteome log2FC	Regulation	Quadrant
ACTA1	−8.47	−0.68	RNA ↓+ Protein ↓	Q3
ALOX15	1.39	−1.01	RNA ↑+ Protein ↓	Q4
ALPL	1.47	−0.96	RNA ↑+ Protein ↓	Q4
CD177	−2.79	−0.35	RNA ↓+ Protein ↓	Q3
CFAP58	−2.87	1.42	RNA ↓+ Protein ↑	Q2
CLEC3B	−2.55	1.32	RNA ↓+ Protein ↑	Q2
COL1A1	−2.86	1.34	RNA ↓+ Protein ↑	Q2
COL1A2	−2.89	0.12	RNA ↓+ Protein ↑	Q2
COL2A1	−2.14	0.82	RNA ↓+ Protein ↑	Q2
ENO3	−1.32	−1.71	RNA ↓+ Protein ↓	Q3
EPB42	−2.95	−0.95	RNA ↓+ Protein ↓	Q3
FBXO7	−1.13	−1.12	RNA ↓+ Protein ↓	Q3
GPX3	−1.80	1.25	RNA ↓+ Protein ↓	Q2
H3-3A	−1.21	−1.10	RNA ↓+ Protein ↓	Q3
HBB	−2.84	−0.87	RNA ↓+ Protein ↓	Q3
INMT	−2.77	−1.18	RNA ↓+ Protein ↓	Q3
LTF	−2.28	−0.60	RNA ↓+ Protein ↓	Q3
MYH1	−4.01	−1.92	RNA ↓+ Protein ↓	Q3
RHAG	−2.42	−0.88	RNA ↓+ Protein ↓	Q3
SPTA1	−2.67	−1.18	RNA ↓+ Protein ↓	Q3
SPTB	−3.37	−1.26	RNA ↓+ Protein ↓	Q3
TNNT3	−6.88	1.75	RNA ↓+ Protein ↑	Q2

### Immune-growth trait correlation

**Figure 35:**
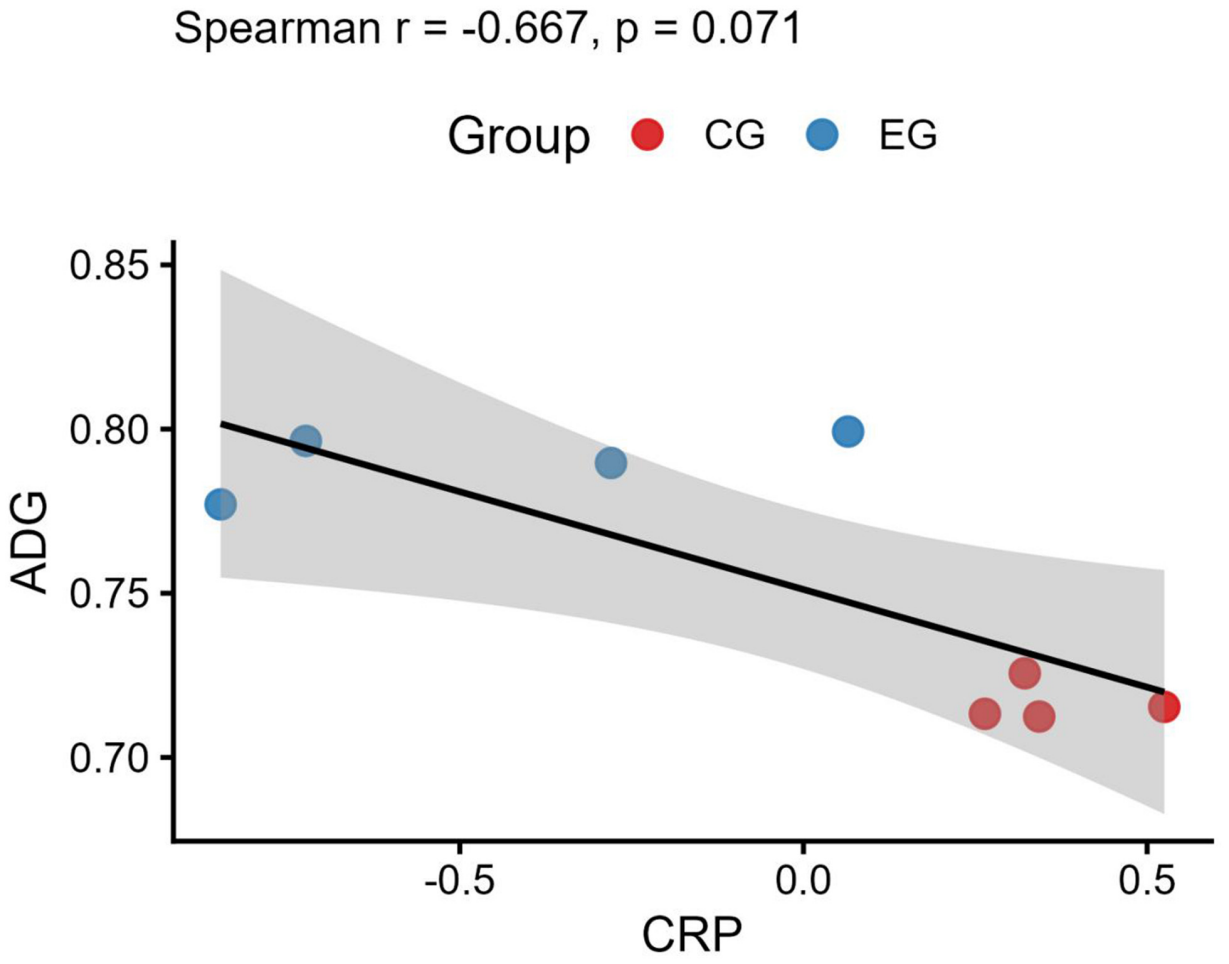
CRP-ADG correlation.

**Figure 36:**
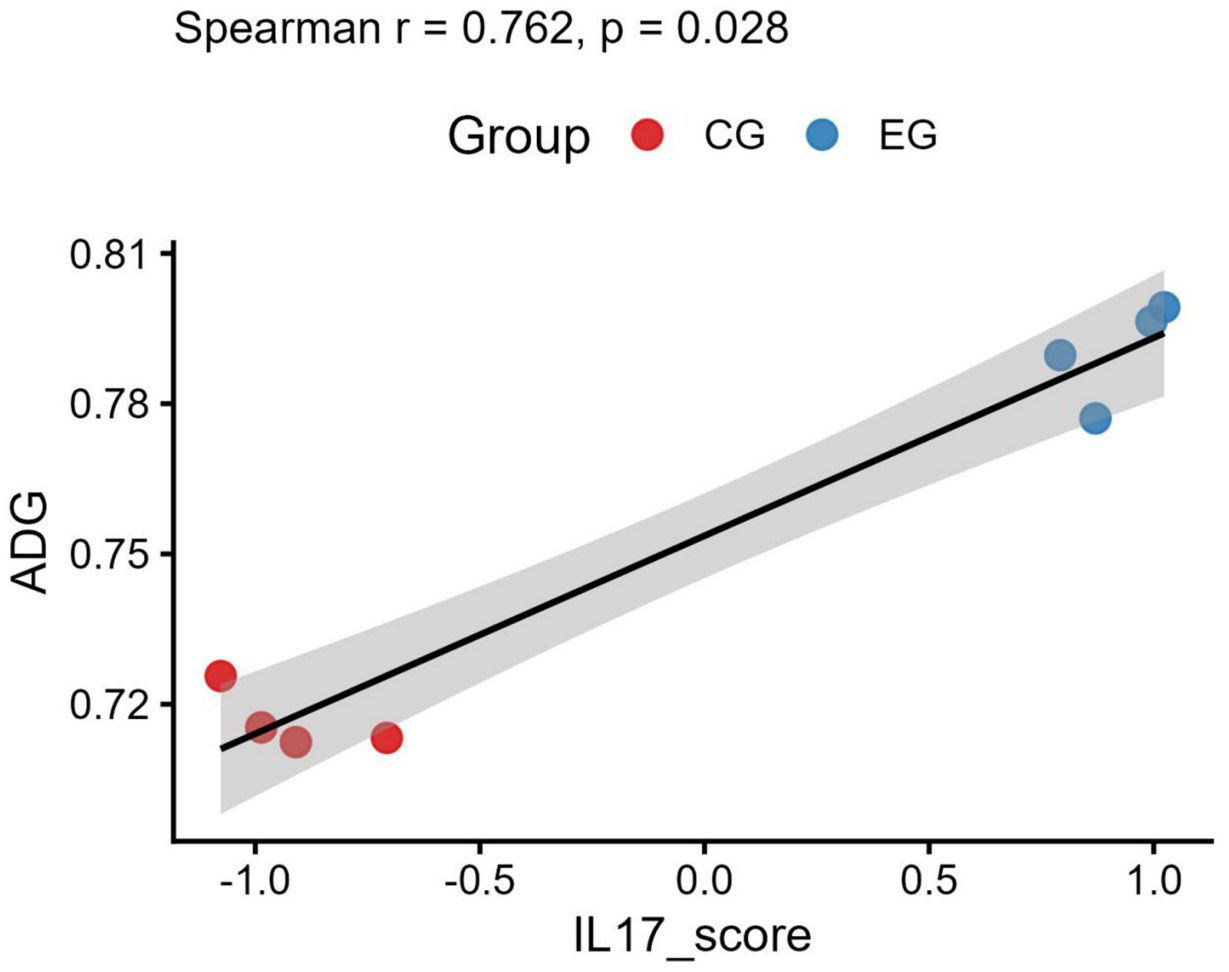
IL-17-ADG correlation.

**Figure 37:**
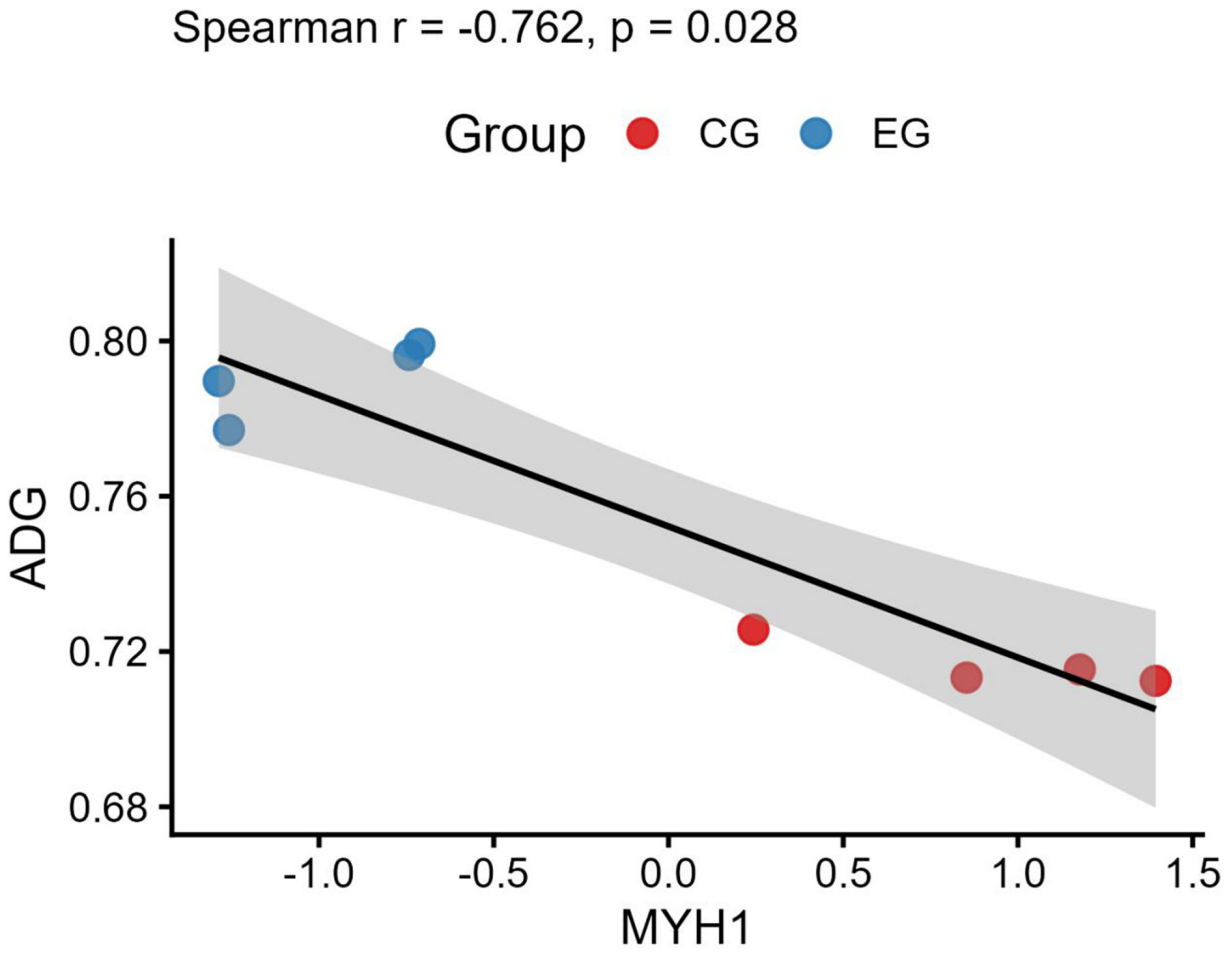
MYH1-ADG correlation.

**Figure 38:**
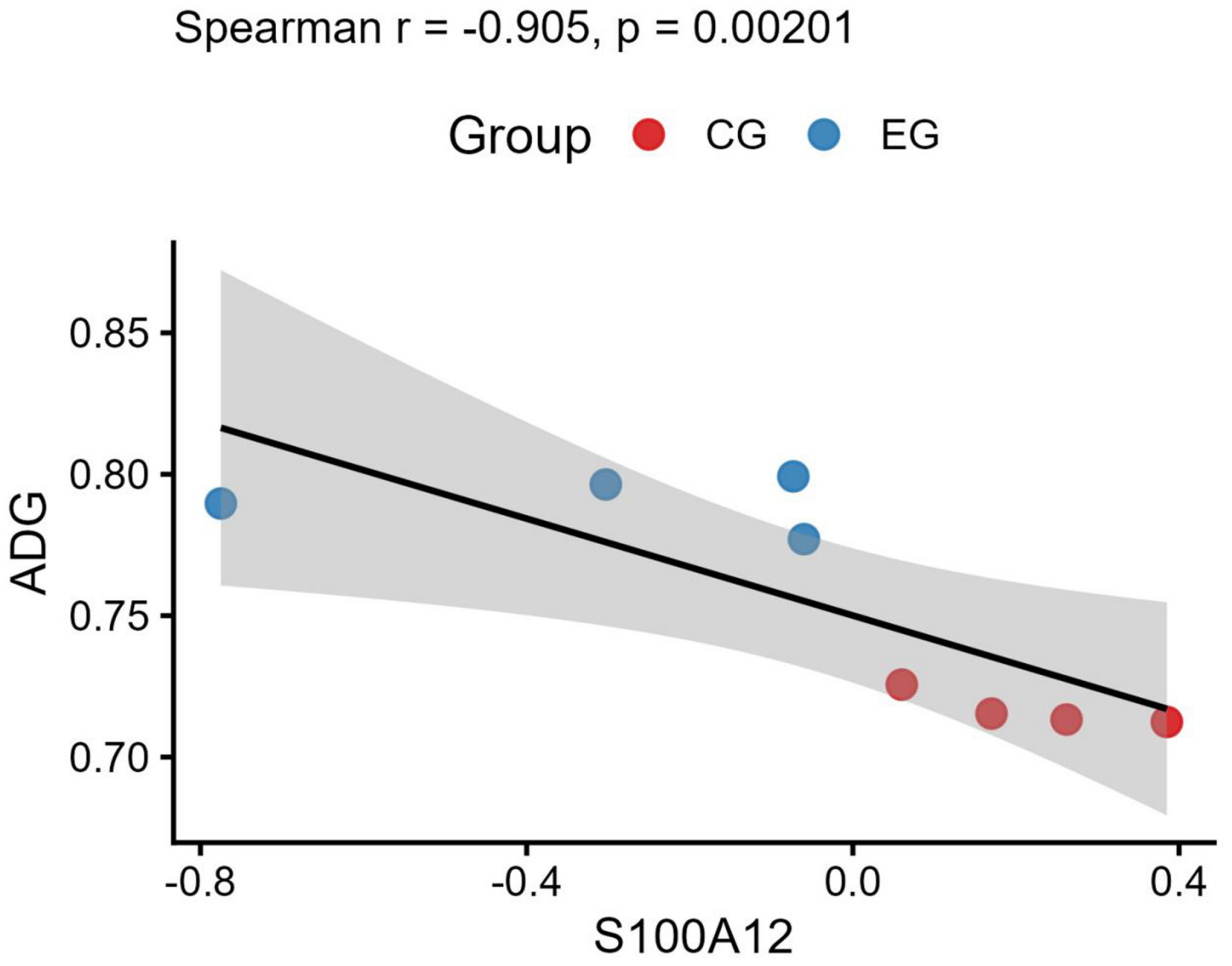
S100A12-ADG correlation.

**Figure 39:**
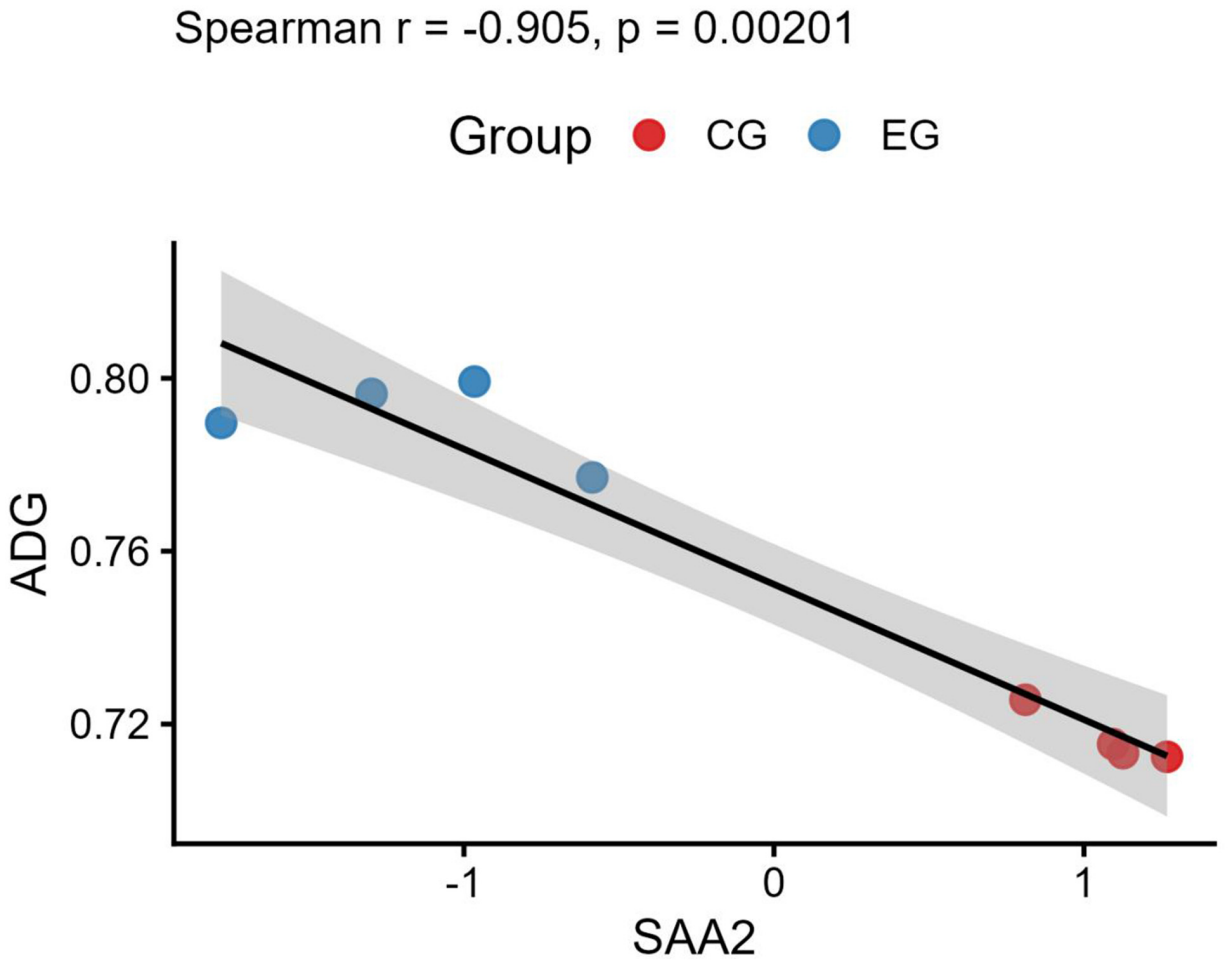
SAA2-ADG correlation.

**Figure 40:**
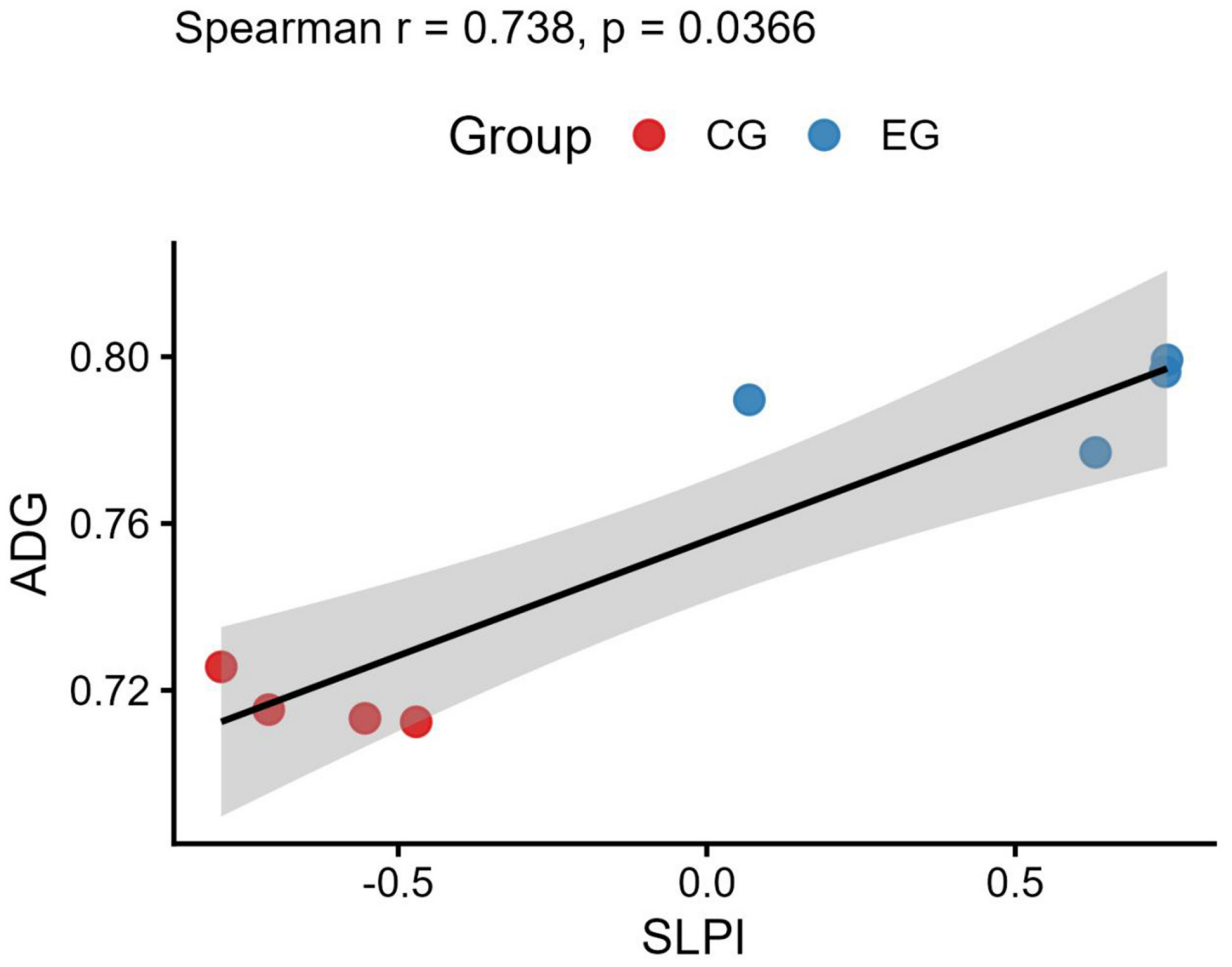
SLPI-ADG correlation.

**Figure 41:**
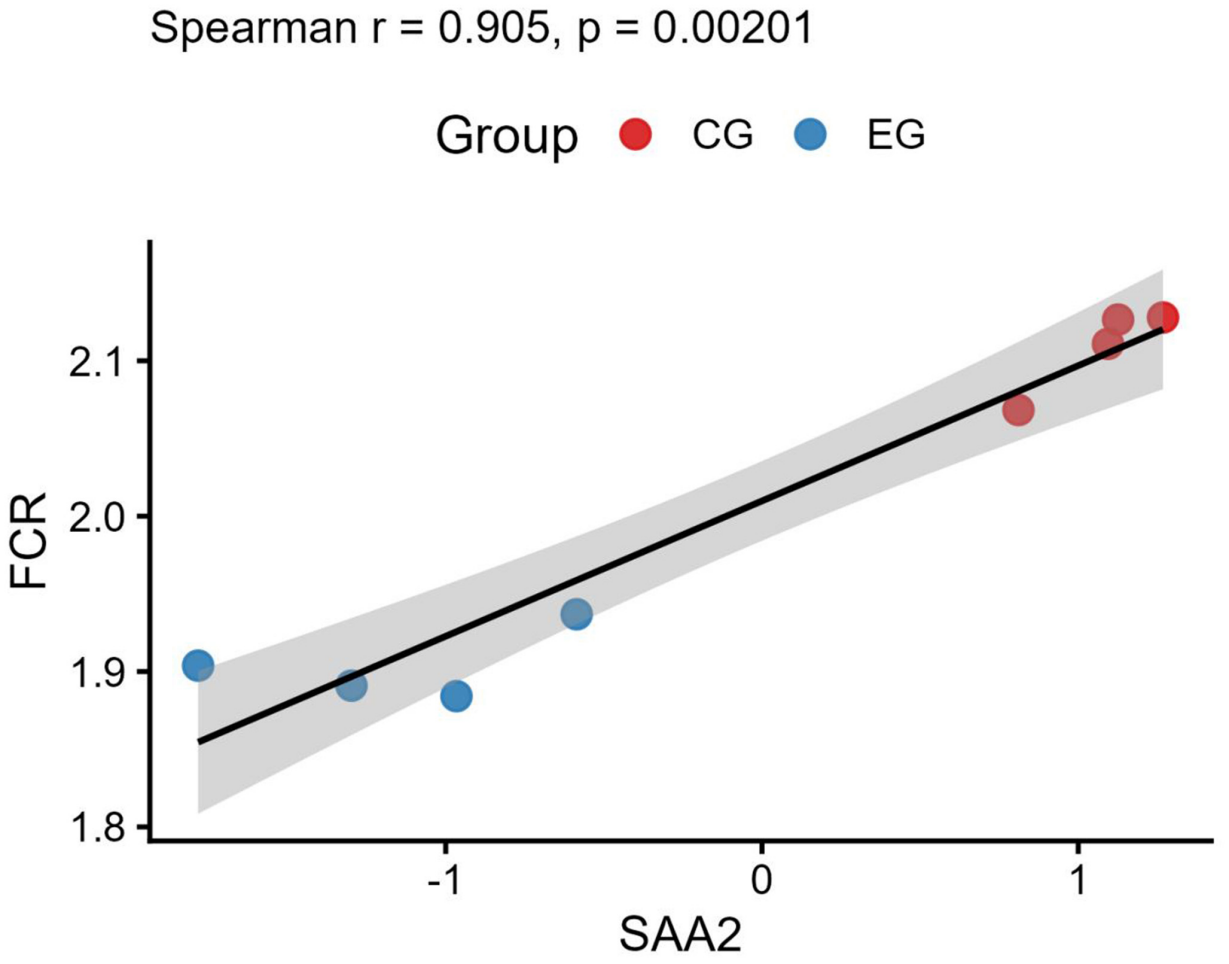
SAA2-FCR correlation.

**Figure 42:**
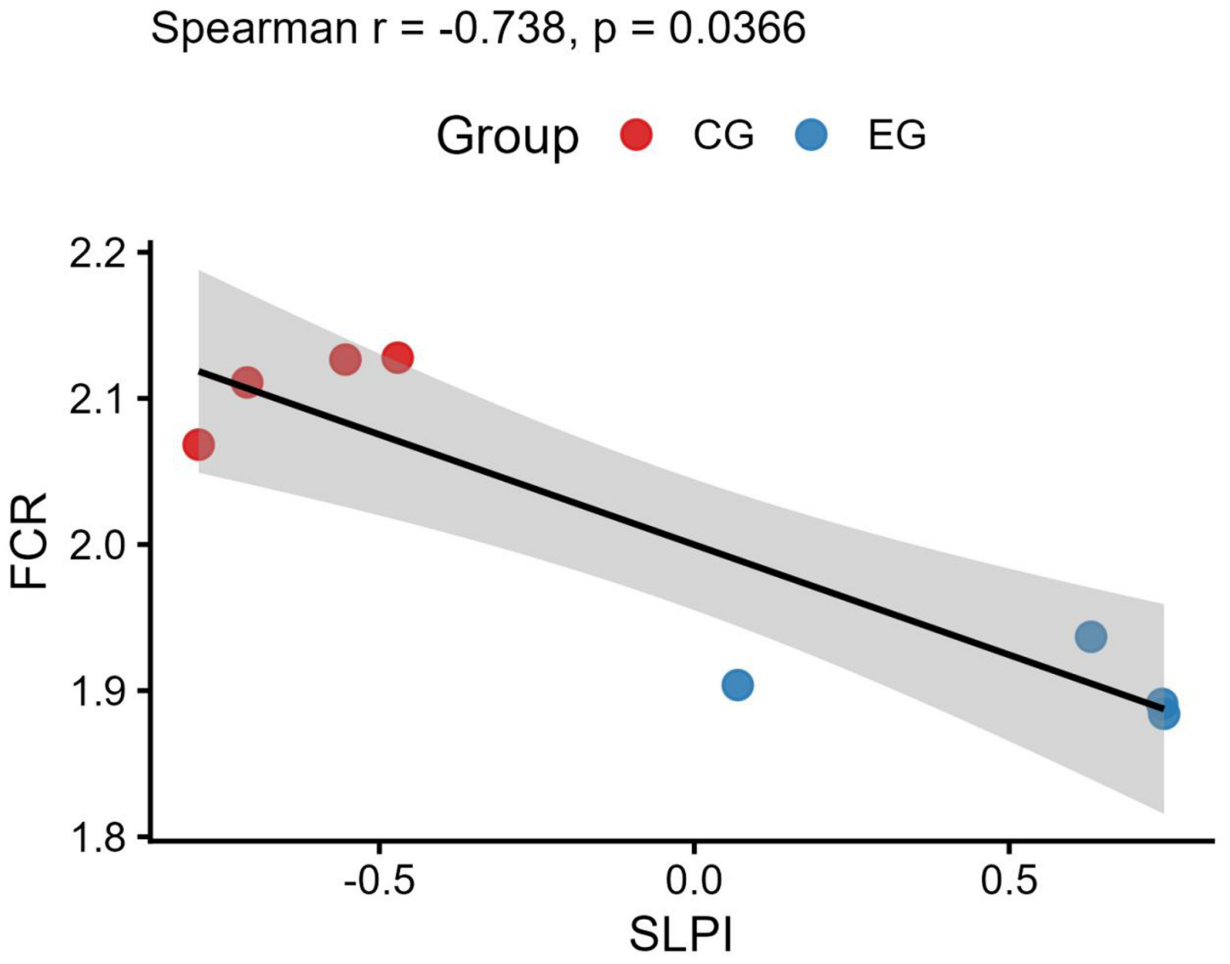
SLPI-FCR correlation.

**Figure 43:**
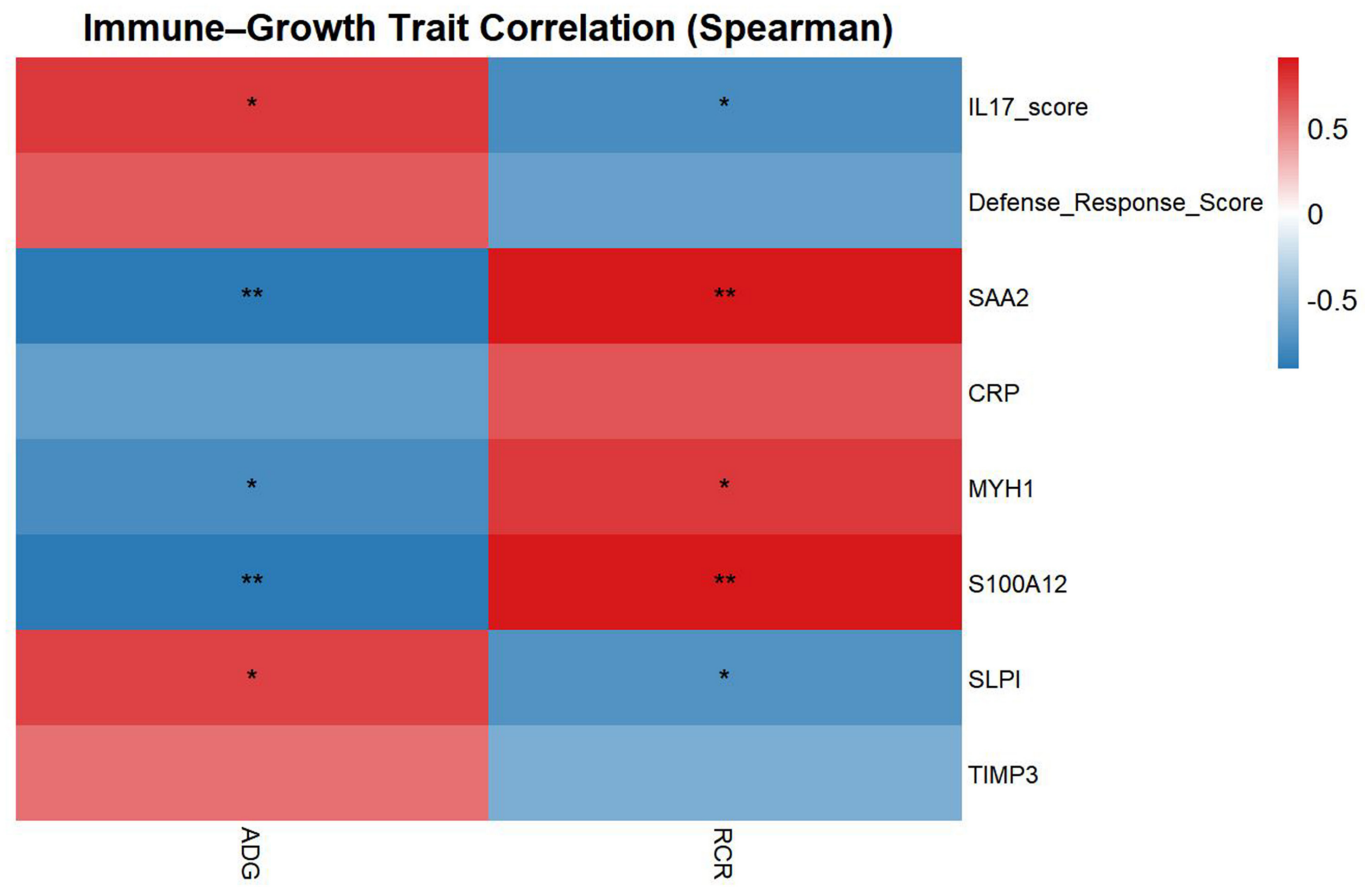
Immune-growth trait correlation. The single asterisk (*) represents *P* < 0.05 which means differences, and double asterisks (**) represent *P* < 0.01, denoting significant differences.

To investigate the relationship between immune-inflammatory status and growth performance, Spearman correlation analysis was conducted between key immune-related proteins, pathway scores, and growth traits (ADG and FCR). Pro-inflammatory proteins were generally negatively associated with growth performance. SAA2 and S100A12 showed significant negative correlations with ADG and positive correlations with FCR (*P* < 0.01), while MYH1 exhibited similar correlations at a lower significance level (*P* < 0.05). CRP displayed the same trend but without statistical significance. Conversely, anti-inflammatory and immune defense-related factors showed favorable associations with growth. IL17_score and SLPI were positively correlated with ADG and negatively correlated with FCR (P <0.05) (Figures 35–43).

### Validation of transcriptomic findings by qRT-PCR and western blot

**Figure 44:**
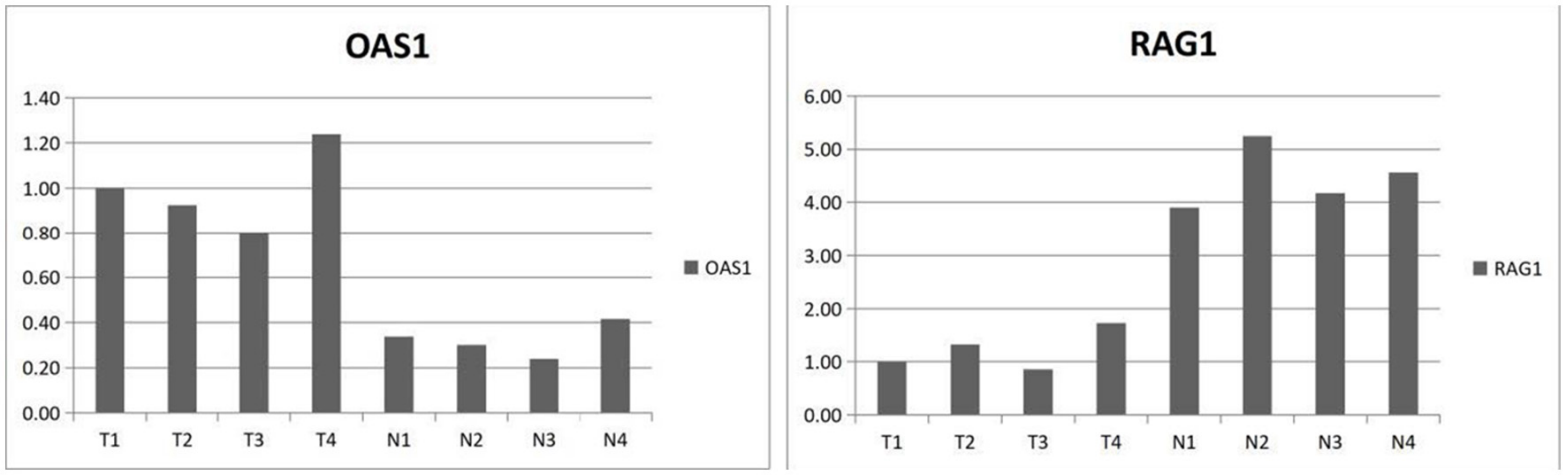
Fold change (FC) of OAS1 and RAG1 in the treated group and control group.

**Figure 45:**
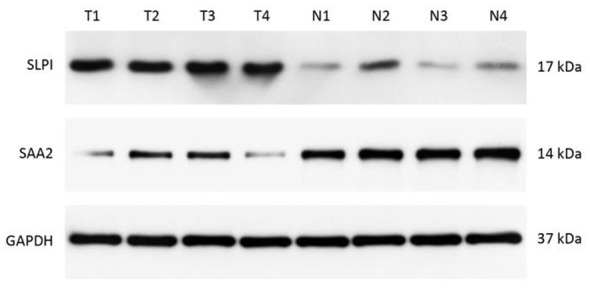
Western Blot result of SLPI and SAA2.

**Figure 46:**
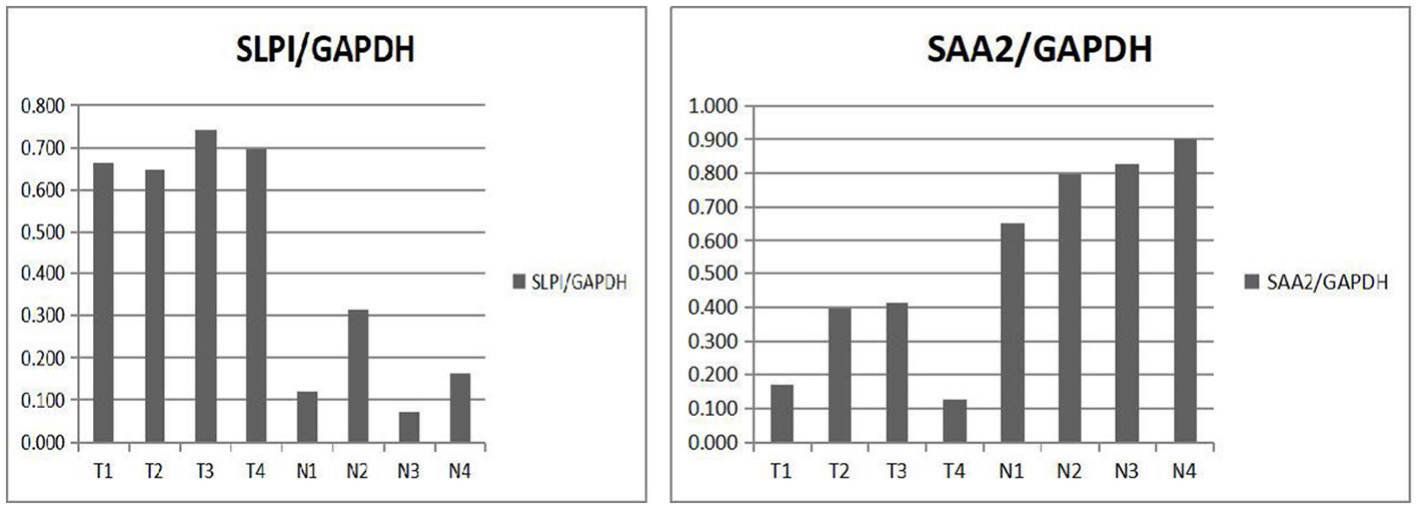
Gray-scale ratio of SLPI and SAA2 in the treated group and control group.

**Figure 47:**
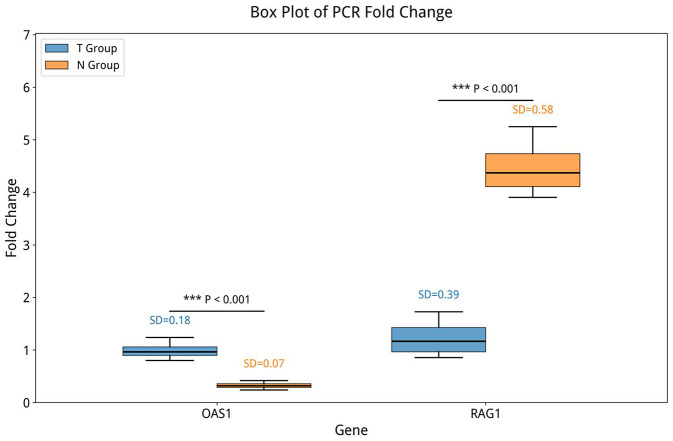
Box plot of PCR fold change of OAS1 and RAG1. The triple asterisk symbol (***) presented an extremely significant statistical difference, corresponding to *P* < 0.001.

**Figure 48:**
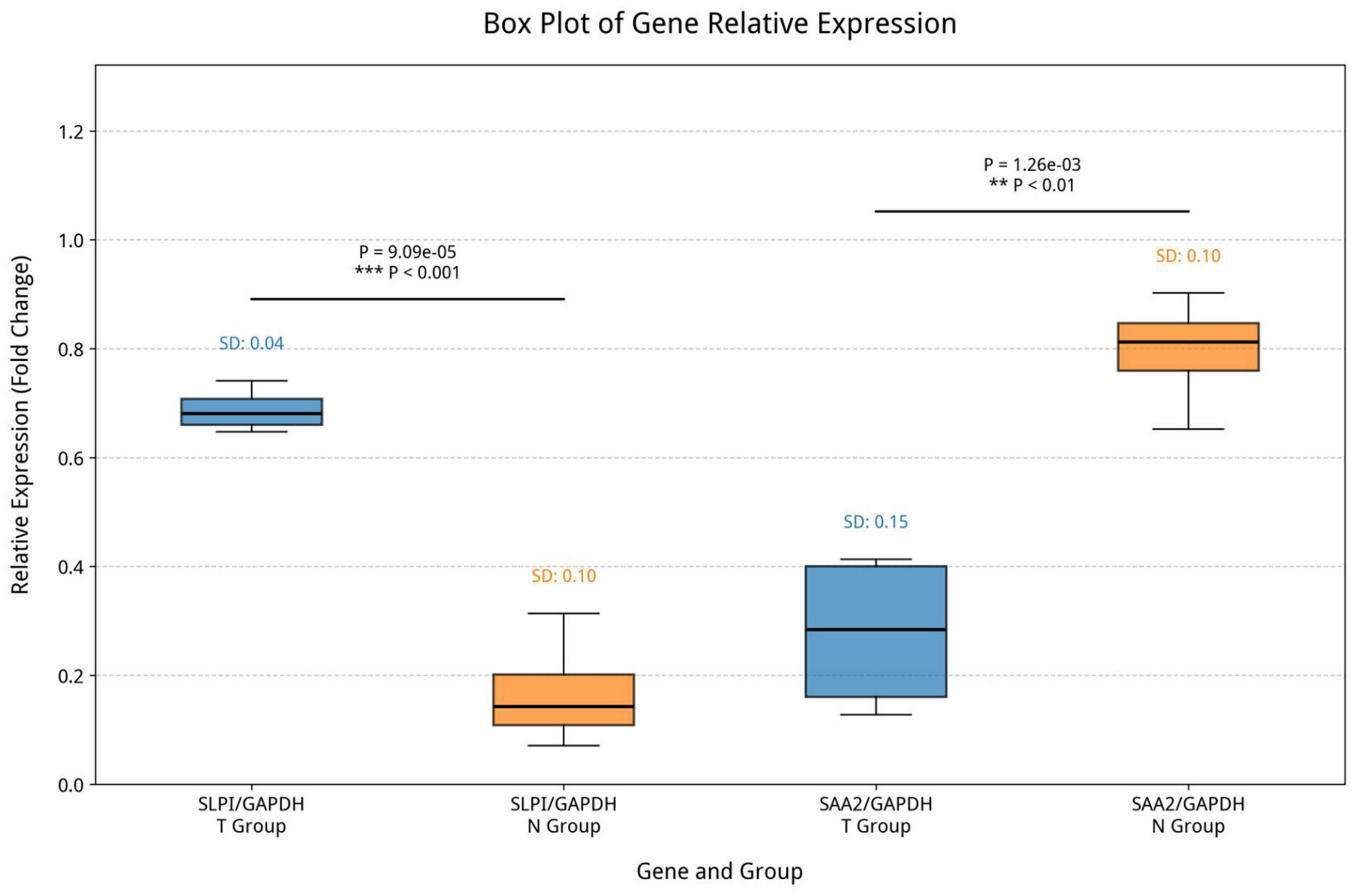
Box plot of WB fold change SLPI and SAA2.

To corroborate the RNA-seq results, key differentially expressed genes were validated at both the transcriptional and translational levels. Quantitative real-time PCR (qRT-PCR) analysis confirmed that OAS1 mRNA expression was significantly up-regulated in the biochar-treated group, whereas RAG1 transcript levels were markedly down-regulated relative to controls (*P* < 0.05; Figure 44). Consistent with the transcriptomic trends, Western blot analysis revealed elevated SLPI protein abundance in the experimental group, while SAA2 protein expression was significantly reduced following biochar supplementation (Figures 45, 46). Both qPCR and Western blot validation analyses showed significant differences between the treatment and control groups (Figures 47, 48). These orthogonal validation results support the reliability of the RNA-seq data and reinforce the conclusion that biochar modulates immune-related gene expression in field-raised pigs.

## Discussion

Dietary inclusion of 3% biochar has been shown to enhance growth performance and feed efficiency in broilers, alongside improvements in digestive enzyme activity, gut microbiota composition, and antioxidant capacity, with greater benefits observed compared with lower inclusion levels such as 1.5% (18). At this supplementation level, favorable modulation of the intestinal environment has also been associated with increased cecal acetate production without compromising microbial diversity, ultimately contributing to improved feed conversion efficiency and overall growth performance throughout the rearing period (17). The beneficial effects of dietary biochar supplementation on gut flora regulation can be explained through multiple complementary mechanisms. Owing to highly porous structure, large specific surface area, and strong adsorption capacity of biochar, it may provide favorable habitats for microbial colonization, thereby enhancing the proliferation and metabolic activity of beneficial bacteria and improving microbial ATP production and feed utilization efficiency (36). In parallel, its adsorptive properties enable the binding of toxic compounds and metabolic waste in the gut, reducing energy losses and contributing to a more stable intestinal environment conducive to improved animal performance (37). Consistently, biochar has been reported to promote the growth of beneficial microorganisms such as lactic acid bacteria while inhibiting pathogenic species including Escherichia coli (22, 38). In addition to microbial modulation, biochar may contribute to the stabilization of intestinal pH through functional groups on its surface such as hydroxyl and carboxyl groups, which can adsorb excess H^+^ or OH^−^ ions in the gastrointestinal tract through ion-exchange processes, thereby exerting a buffering effect that helps prevent drastic fluctuations in digestive tract pH (39, 40). Importantly, gut microbial dysbiosis can promote excessive lipopolysaccharide (LPS) accumulation, activating oxidative stress and inflammatory responses via the TLR4/NLRP3 pathway and ultimately compromising intestinal barrier integrity. Biochar-mediated modulation of the gut microbiota may reduce LPS levels while increasing short-chain fatty acid production, such as butyrate, and enhancing β-defensin-2 secretion, thereby improving antimicrobial defense and maintaining intestinal barrier function (41). Furthermore, Biochar can modulate microbial community structure and enhance carbohydrate and amino acid metabolic activities, thereby promoting the enrichment of beneficial bacteria and strengthening microbial functional interactions, ultimately improving overall microbial ecological balance. (42). The resulting healthier microbial ecosystem can also contribute to improved host immune function, reduced disease incidence, and decreased reliance on antibiotics (43, 44). In this study, there is limited concordance between transcriptomic and proteomic changes, and these discrepancies may be caused by post-transcriptional regulation (e.g., translational repression and miRNA activity), differences in mRNA and protein stability, and altered protein turnover and degradation. This may also be attributed to the complex multi-level biological regulation induced by biochar supplementation through modulation of the microbial and metabolic environment.

The gut represents the largest immune and metabolic organ in animals, and its physiological status is closely interconnected with peripheral circulation through the gut-blood axis (28, 45). Recent multi-omics studies have provided increasing evidence supporting the interconnection between the gut health and circulating blood parameters. Tretiak demonstrated that gut microbiota dysbiosis and intestinal barrier disruption in broilers induce profound remodeling of the plasma proteome, with differentially expressed proteins significantly enriched in immune activation, complement cascade, acute phase response, and extracellular matrix remodeling pathways. Notably, several circulating proteins, including CFD, HPS5, and MASP2, were identified as robust biomarkers of intestinal injury, showing strong sensitivity in reflecting gut pathological status. Similarly, in necrotic enteritis models, blood proteomic signatures accurately mirrored the severity of intestinal inflammation and barrier dysfunction, highlighting the feasibility of using blood-based omics as a non-invasive and dynamic surrogate for intestinal health assessment (46). Intestinal infection and inflammatory responses are frequently accompanied by marked increases in circulating acute-phase proteins, including serum amyloid A (SAA) and haptoglobin (PIT54), whose abundances are closely associated with the severity of intestinal damage (47). These observations suggest that peripheral blood immune biomarkers can serve as sensitive indicators of gut inflammatory status. Moreover, alterations in gut microbiota composition and microbial metabolic activity induced by nutritional interventions have been shown to induce coordinated changes in plasma metabolites and immune-related gene expression, suggesting that blood multi-omics profiles effectively capture host responses originating from the intestinal environment (45). In the present study, the pronounced upregulation of pathways governing complement and coagulation cascades, cytokine-receptor interactions, and the intestinal IgA immune network indicates a potent enhancement of innate immune surveillance and mucosal defense. Protein domain enrichment analysis revealed higher abundance of ribosomal L5P C-terminal, elongation factor Tu C-terminal, and CCN3 Nov-like TSP1 domains in the experimental group, suggesting enhanced protein synthesis-related activity and immune cell migration/adhesion (48). Moreover, the coordinated upregulation of chemokines (e.g., CCL12, CCL19, CCL21) coupled with enriched cytokine-receptor crosstalk suggests that biochar may fine-tune leukocyte recruitment and promote a resolution-oriented immune phenotype. This aligns with the observed downregulation of acute-phase proteins such as serum amyloid A2 (SAA2) and C-reactive protein (CRP) (49).

Beyond immunomodulation, the significant enrichment of differentially expressed molecules in muscle system processes and actin filament-based biological pathways points to a protective effect of biochar on skeletal muscle homeostasis. The bidirectional communication between systemic immunity and muscle physiology, often termed immune-muscle crosstalk, is increasingly recognized as a critical determinant of growth efficiency and tissue repair. Cytokines such as IL-6, which mediate signaling between immune cells and myocytes, likely serve as pivotal nodes in this interaction (50). Inflammation plays a dual role in skeletal muscle growth, acting as both a necessary component of tissue repair and a potential inhibitor of muscle accretion when sustained or excessive. Evidence suggests that efficient growth is associated with a balanced immune response that supports metabolic activity and nutrient utilization, whereas chronic inflammation diverts resources toward immune defense, thereby impairing growth performance (51). Mechanistically, pro-inflammatory cytokines such as TNF-α, IL-6, and IL-1β activate NF-κB and MAPK-dependent pathways, which suppress myogenic differentiation and reduce muscle protein synthesis. In parallel, chronic inflammation enhances proteolysis via the ubiquitin-proteasome system through upregulation of MuRF1 and Atrogin-1, leading to reduced muscle fiber hypertrophy (52). In addition, inflammatory stress may induce epigenetic modifications, including altered HDAC and DNMT activity, which further suppress myogenic gene expression and enhance muscle atrophy pathways (53). In the present study, pathways related to cytoskeletal dynamics and motor protein activity in muscle such as cytoskeleton in muscle cells, motor proteins, and muscle structure development pathway were down-regulated, it may originate from lower inflammation levels caused lower muscle damage.

A negative association between systemic inflammatory status and growth performance is consistently observed, indicating that elevated inflammation is linked to reduced weight gain and feed efficiency which is consistent with the result of immune-growth trait correlation in this study. One study demonstrated that chronic inflammation activates TLR4/NF-κB and MAPK signaling pathways, leading to excessive production of pro-inflammatory cytokines such as TNF-α, IL-1β, and IL-6. This inflammatory state suppresses appetite, impairs nutrient utilization, and enhances muscle protein degradation, ultimately reducing average daily gain and increasing feed conversion ratio (54). At the molecular level, inflammation disrupts the growth hormone-insulin-like growth factor (GH-IGF) axis by downregulating GH receptor expression and attenuating IGF-1 signaling, thereby weakening anabolic processes and limiting muscle accretion (55). Conversely, reduced inflammatory status is associated with improved immune balance, enhanced metabolic efficiency, and better growth performance, accompanied by increased immunoglobulin levels and reduced tissue damage markers (56). Correlation analysis between growth performance and immune-related indicators in this study further supported a close association between immune status and animal growth. In the biochar-supplemented group, the inflammatory markers SAA2 and CRP were downregulated and negatively correlated with growth traits, suggesting that excessive inflammatory activity may impair growth performance. In contrast, SPLI and the IL-17 signaling pathway, enriched by the upregulated genes FOS, FOSB, and CXCL2, showed positive correlations with growth indicators, indicating that enhanced innate immune defense may contribute to improved growth. Moreover, the decreased abundance of MYH1 in the blood proteome of the treated group may reflect reduced muscle damage resulting from a lower inflammatory burden or enhanced immune protection. Similar results have been reported in several studies (54, 56), suggesting that elevated inflammatory responses may negatively affect muscle growth and integrity, whereas improved immune defense and controlled inflammatory status may help preserve muscle function and promote body weight gain in pigs.

In conclusion, integrated proteomic and transcriptomic analyses demonstrate that dietary supplementation with 3% biochar exerts coordinated immunoregulatory in pigs, coinciding with enhanced growth performance and improved survivability. Notably, a limited concordance between transcriptomic and proteomic profiles was observed, suggesting that biochar-induced effects are regulated through complex multi-layered biological mechanisms, including post-transcriptional regulation and metabolic-microbial interactions. By concurrently upregulating innate immune defense pathways and attenuating inflammatory signaling, while supporting cytoskeletal and muscle-related processes, biochar emerges as a promising nutritional strategy for optimizing health and productivity in swine production. Further mechanistic and translational research is warranted to fully harness its potential in precision livestock nutrition.

## Data Availability

The whole-genome sequencing raw data have been deposited in the NCBI SRA repository under accession number PRJNA1480378, and the serum proteomic mass spectrometry raw data have been deposited in the iProX repository via the ProteomeXchange Consortium under accession number PXD079982.
