# Supplementary material for: Dietary biochar enhances growth performance and feed efficiency in growing pigs evidence from integrated transcriptomic and proteomic analyses
Source: Front Vet Sci. 2026 Jul 2;13:1863140. doi: 10.3389/fvets.2026.1863140 (PMC13372601; doi:10.3389/fvets.2026.1863140)
Supplement: Supplementary file 1 [file Data_Sheet_1.DOC]

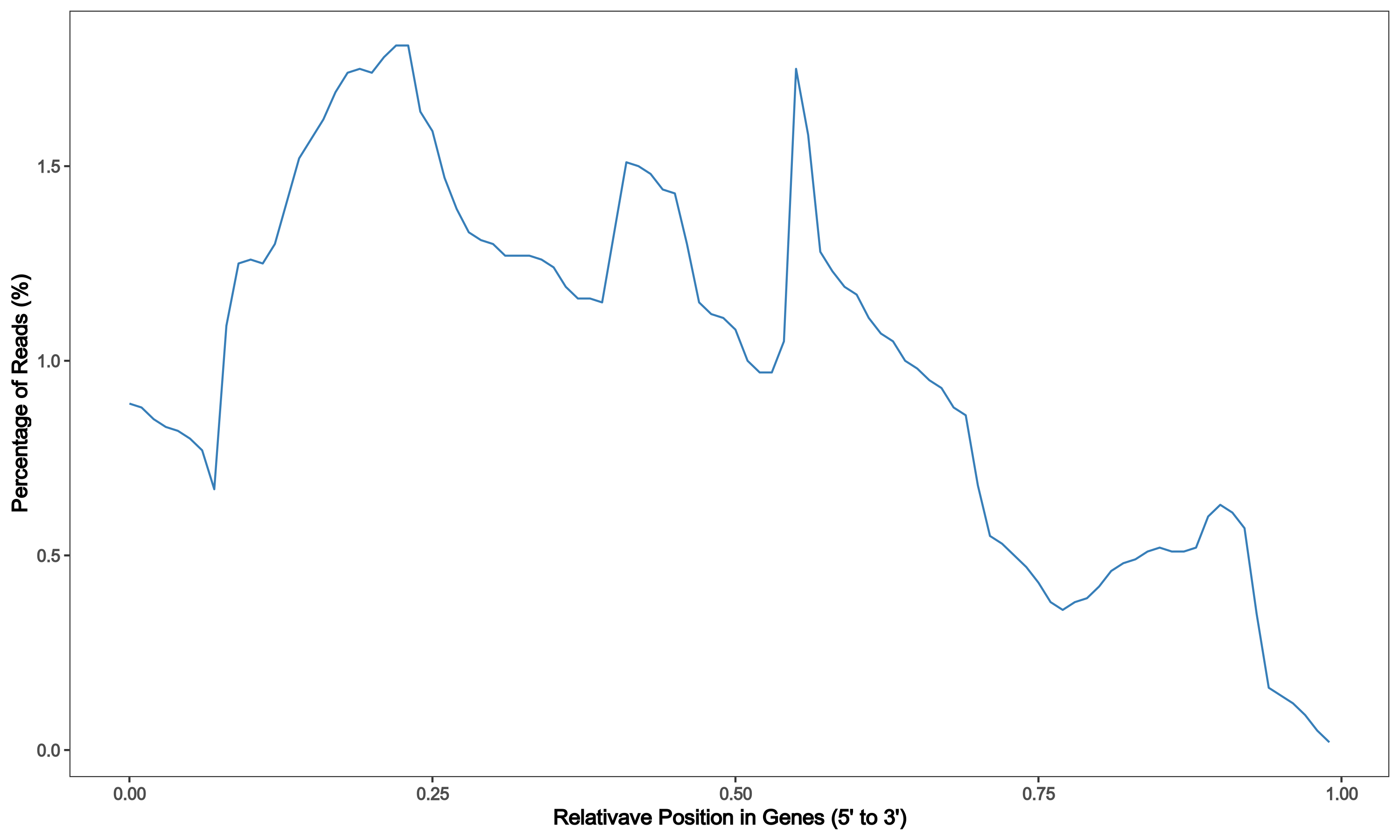

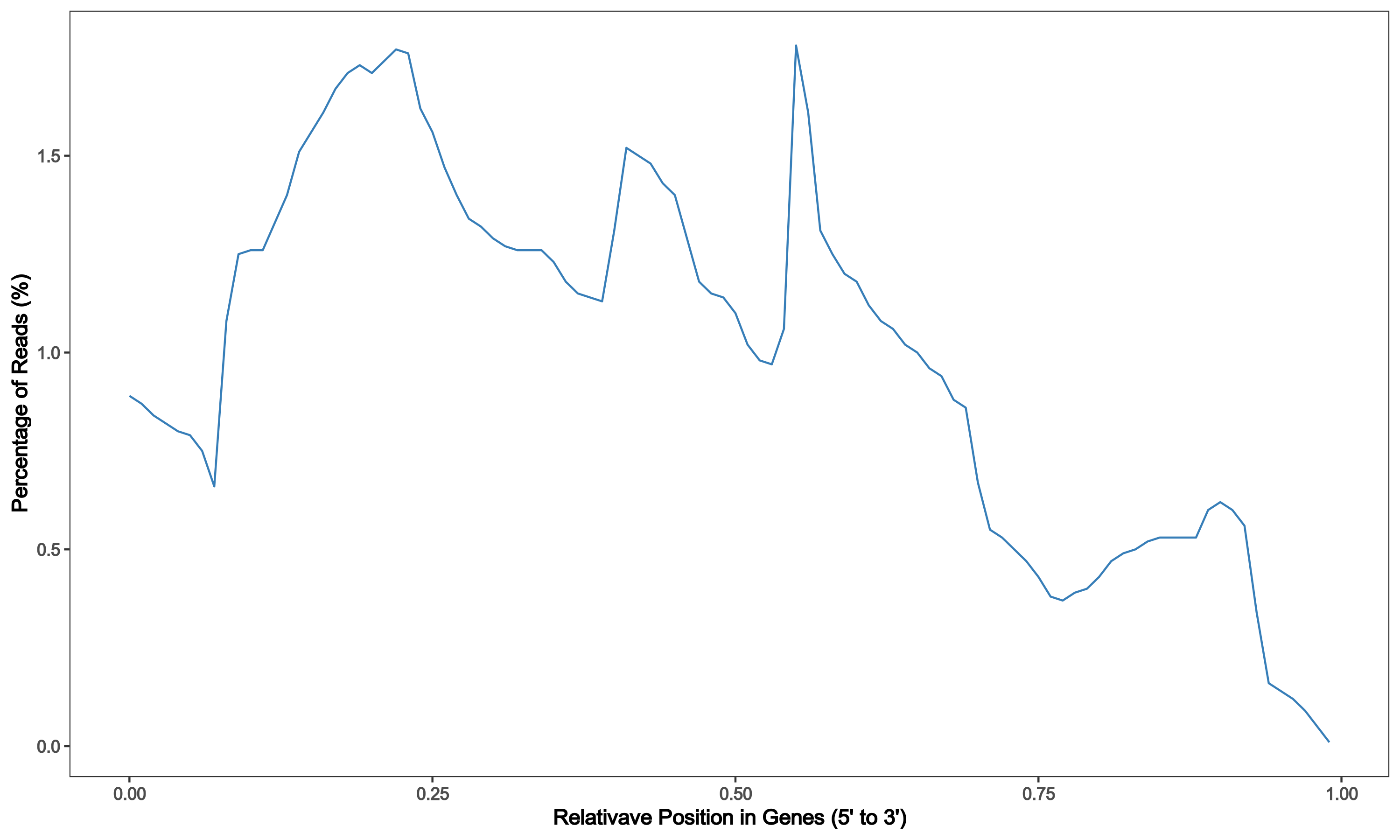


Supplementary Figures 1：N1_randcheck Supplementary Figures 2：N2_randcheck


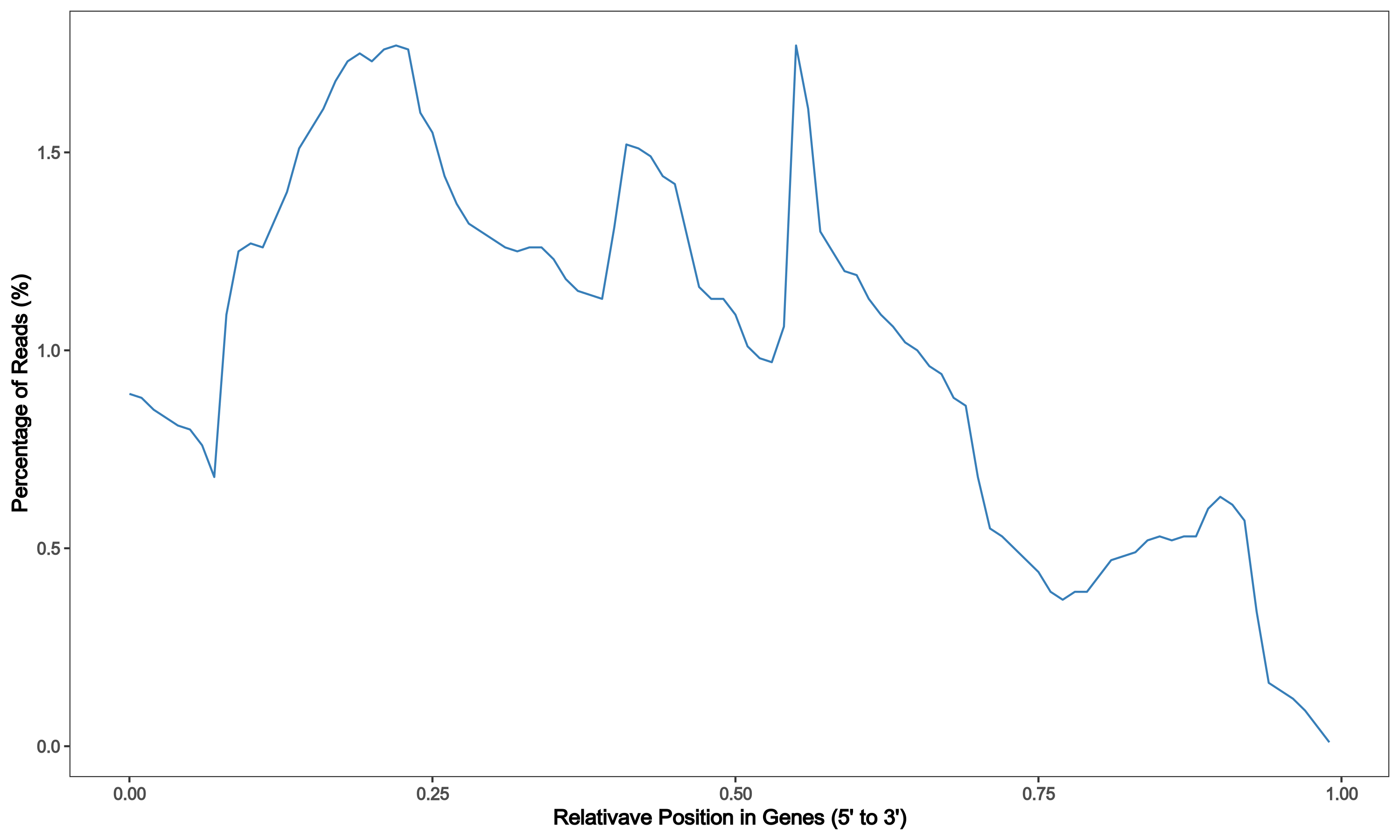

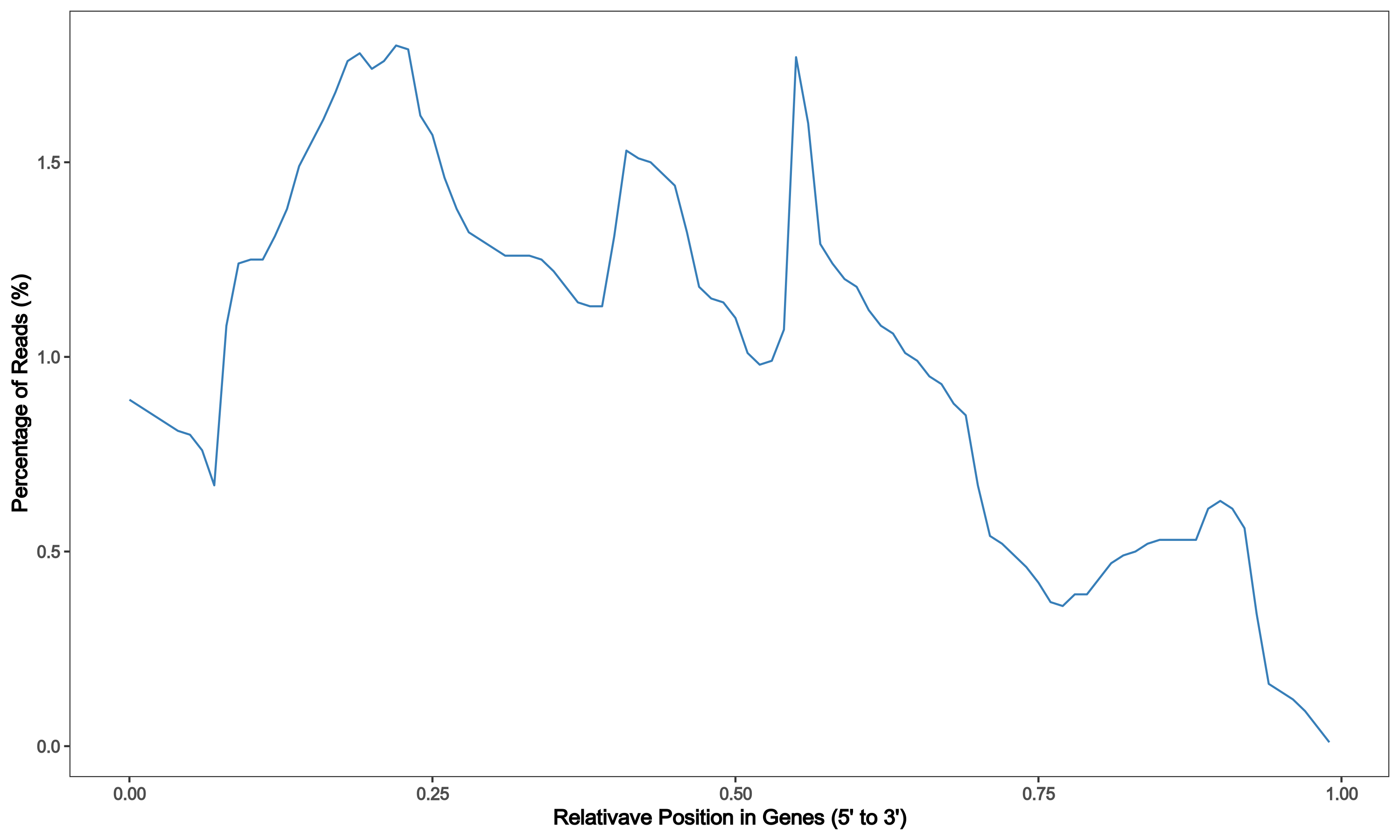


Supplementary Figures 3：N3_randcheck Supplementary Figures 4：N4_randcheck


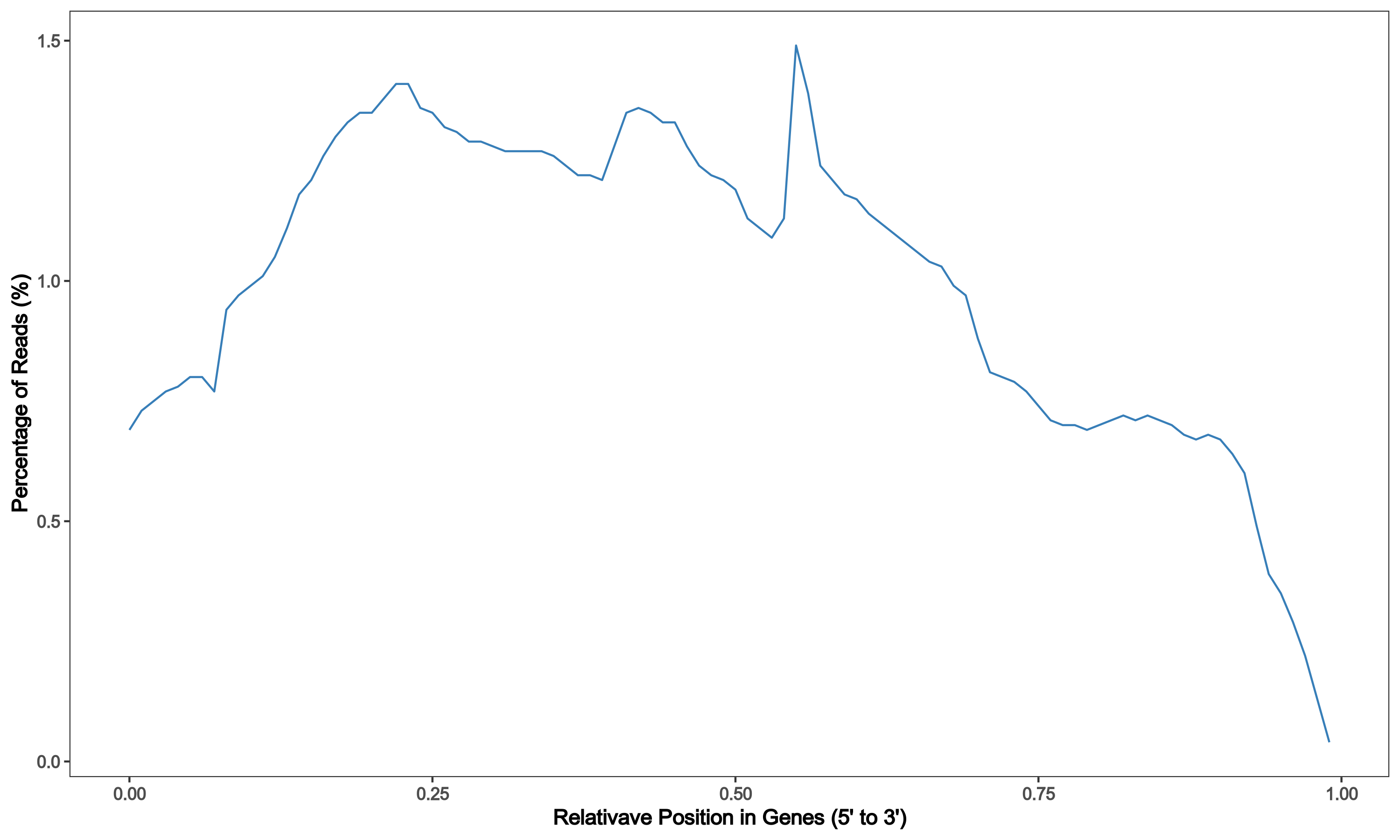

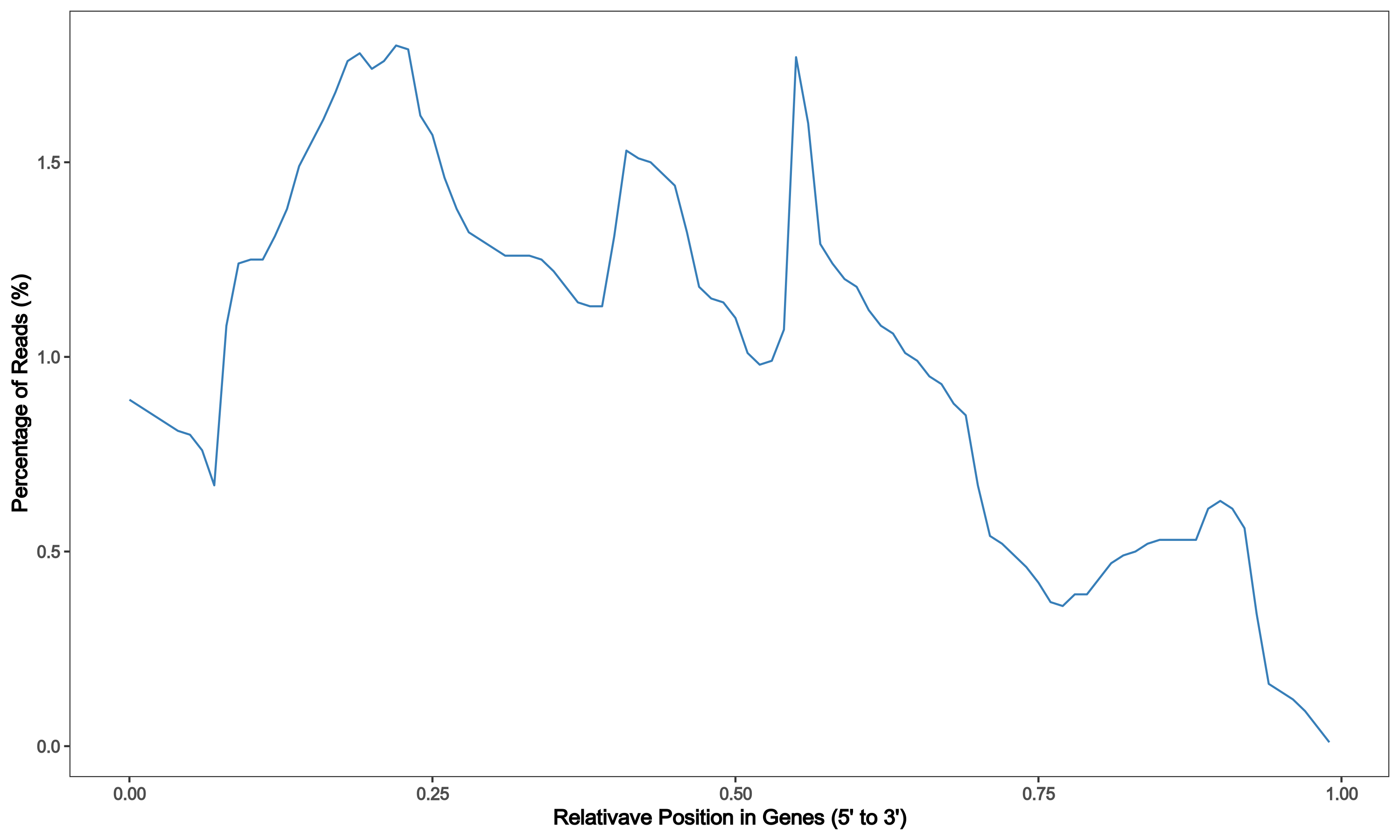


Supplementary Figures 5：T1_randcheck Supplementary Figures 6：T2_randcheck


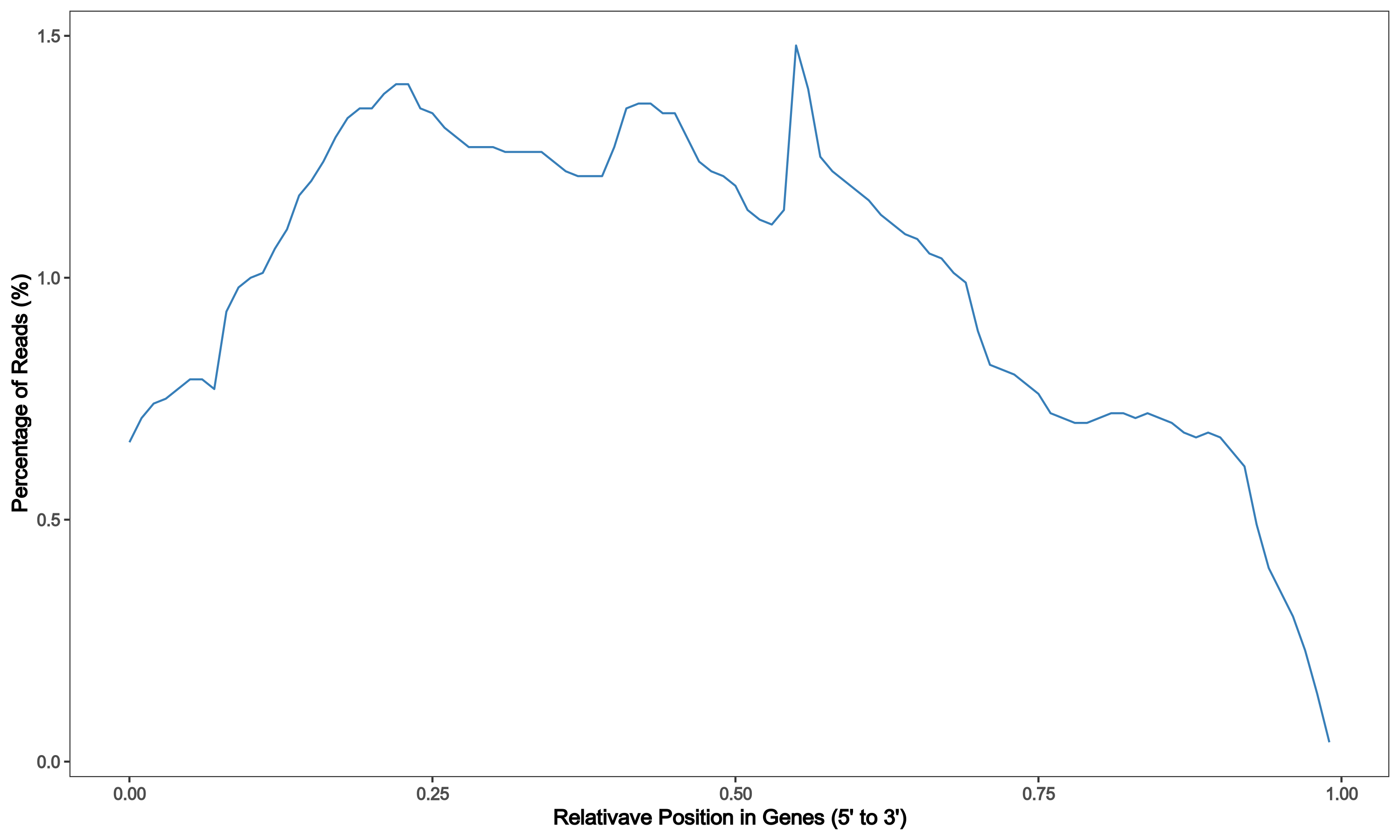

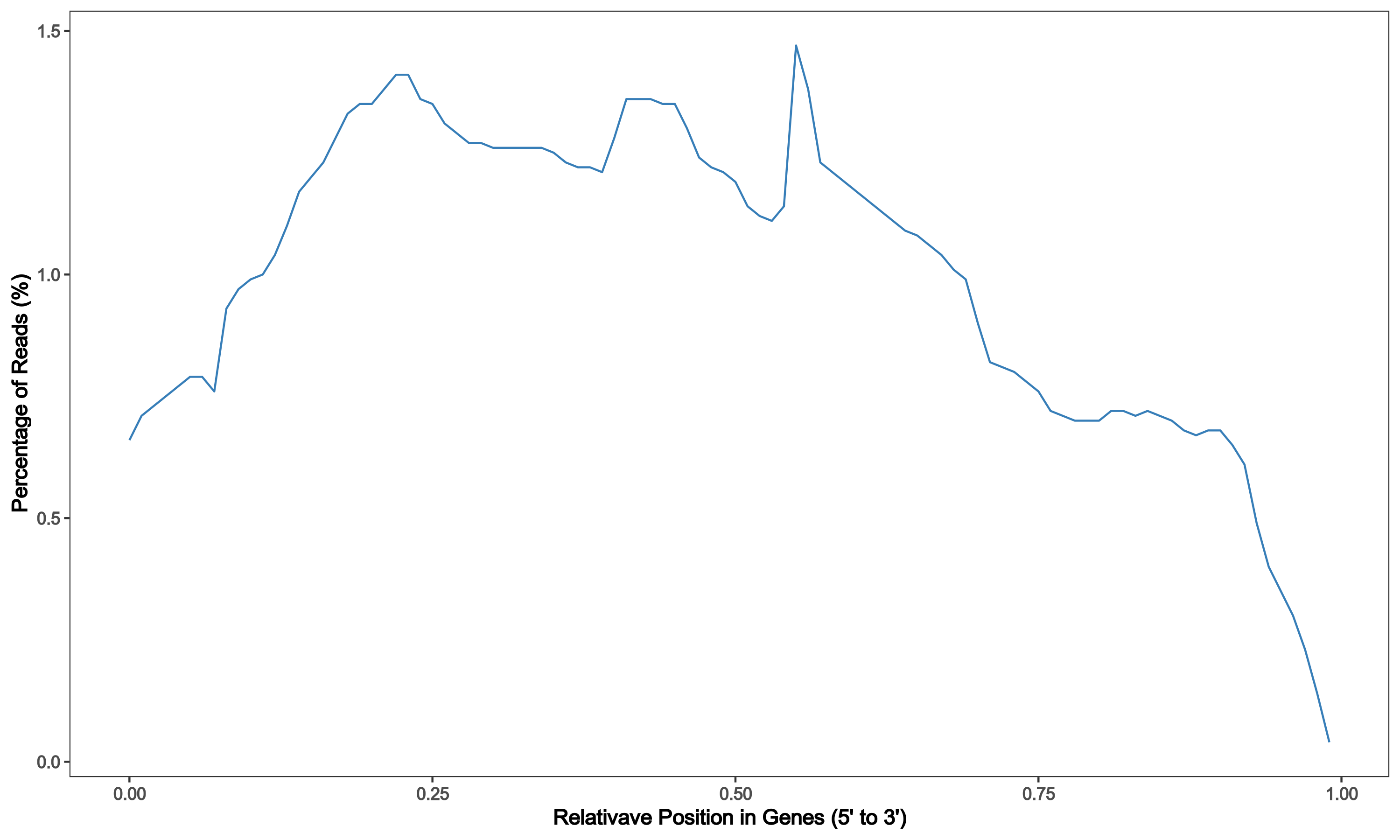


Supplementary Figures 7：T3_randcheck Supplementary Figures 8：T4_randcheck

Supplementary Figures 9：N1.saturation Supplementary Figures 10：N2.saturation


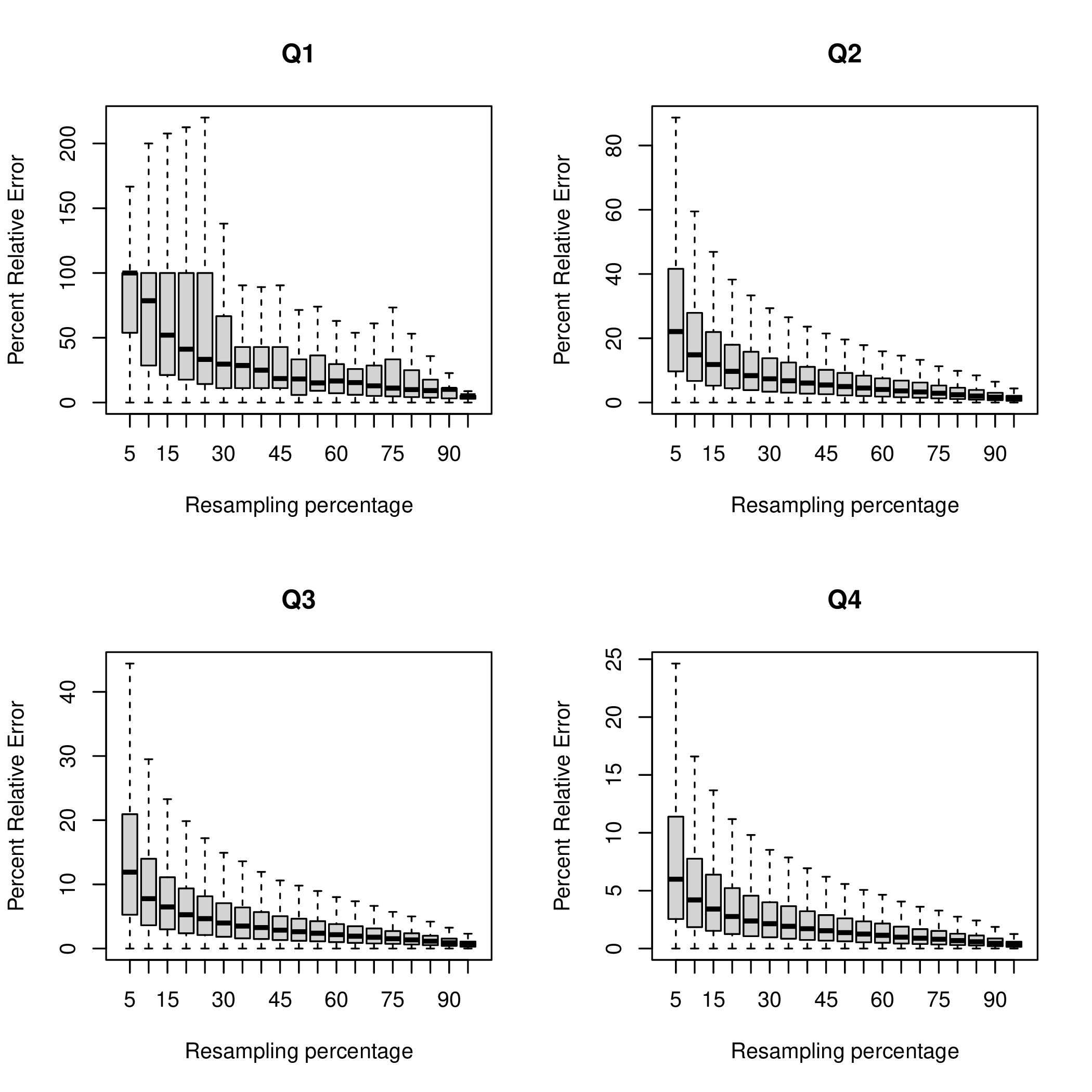

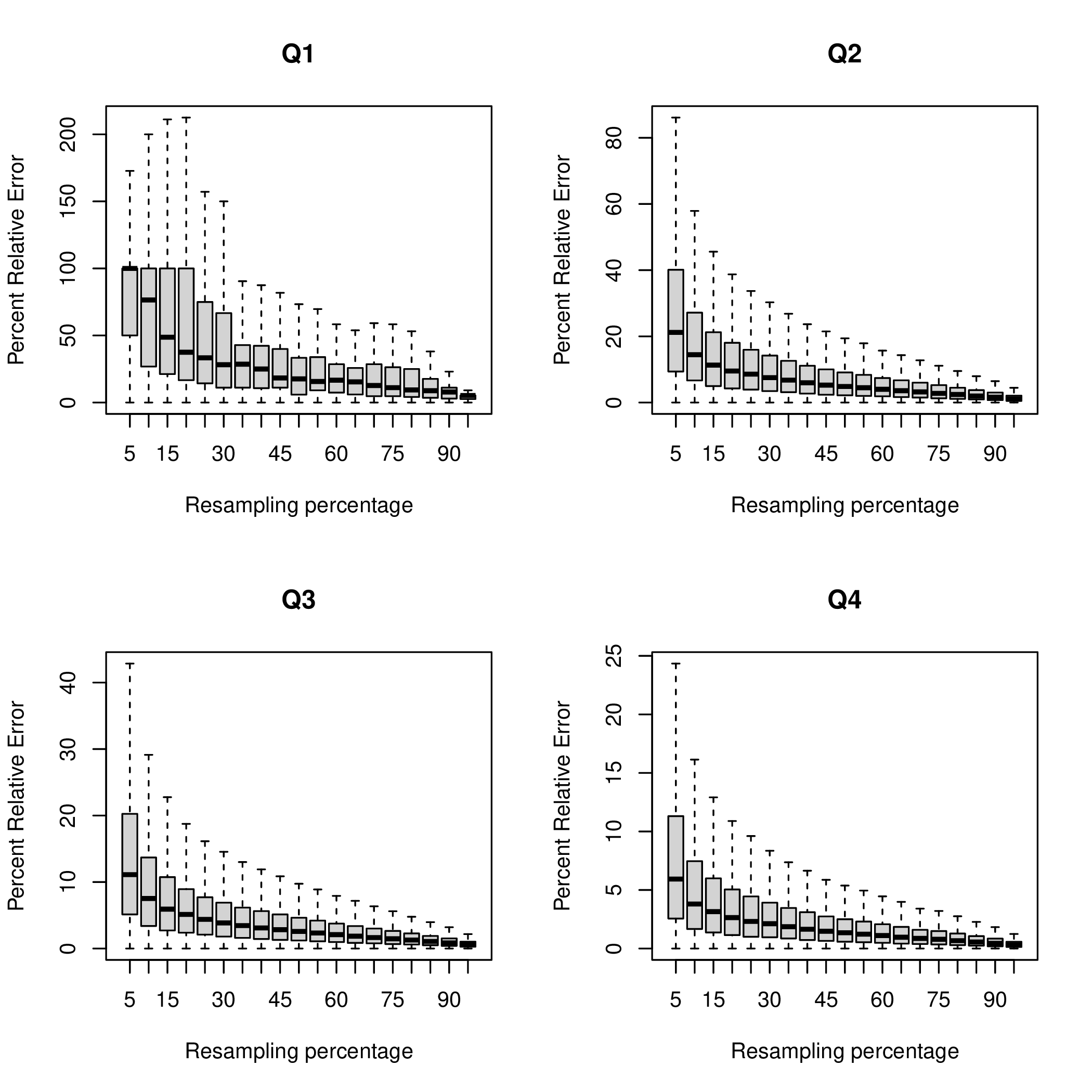


Supplementary Figures 11：N3.saturation Supplementary Figures 12：N4.saturation


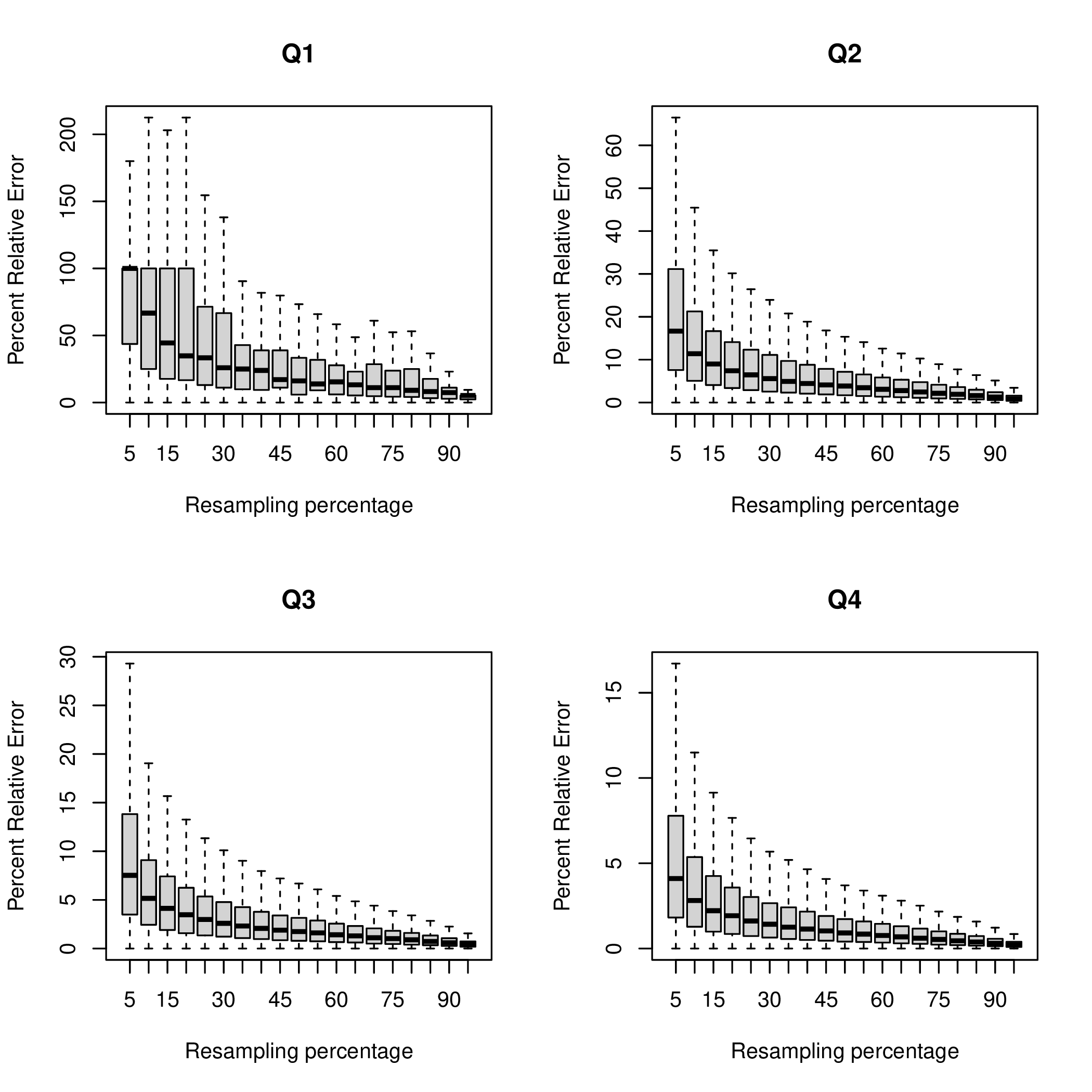

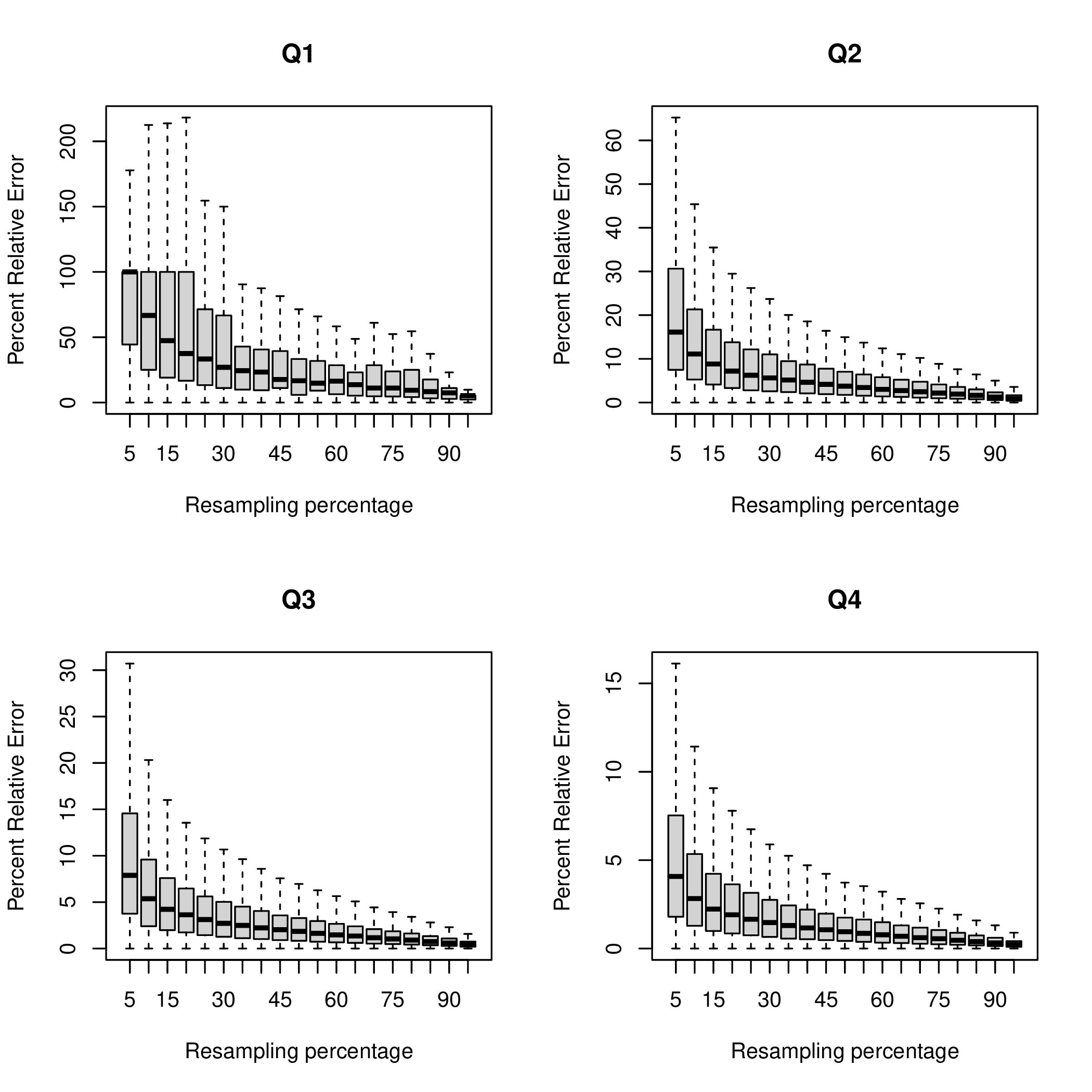


Supplementary Figures 13：T1.saturation Supplementary Figures 14：T2.saturation


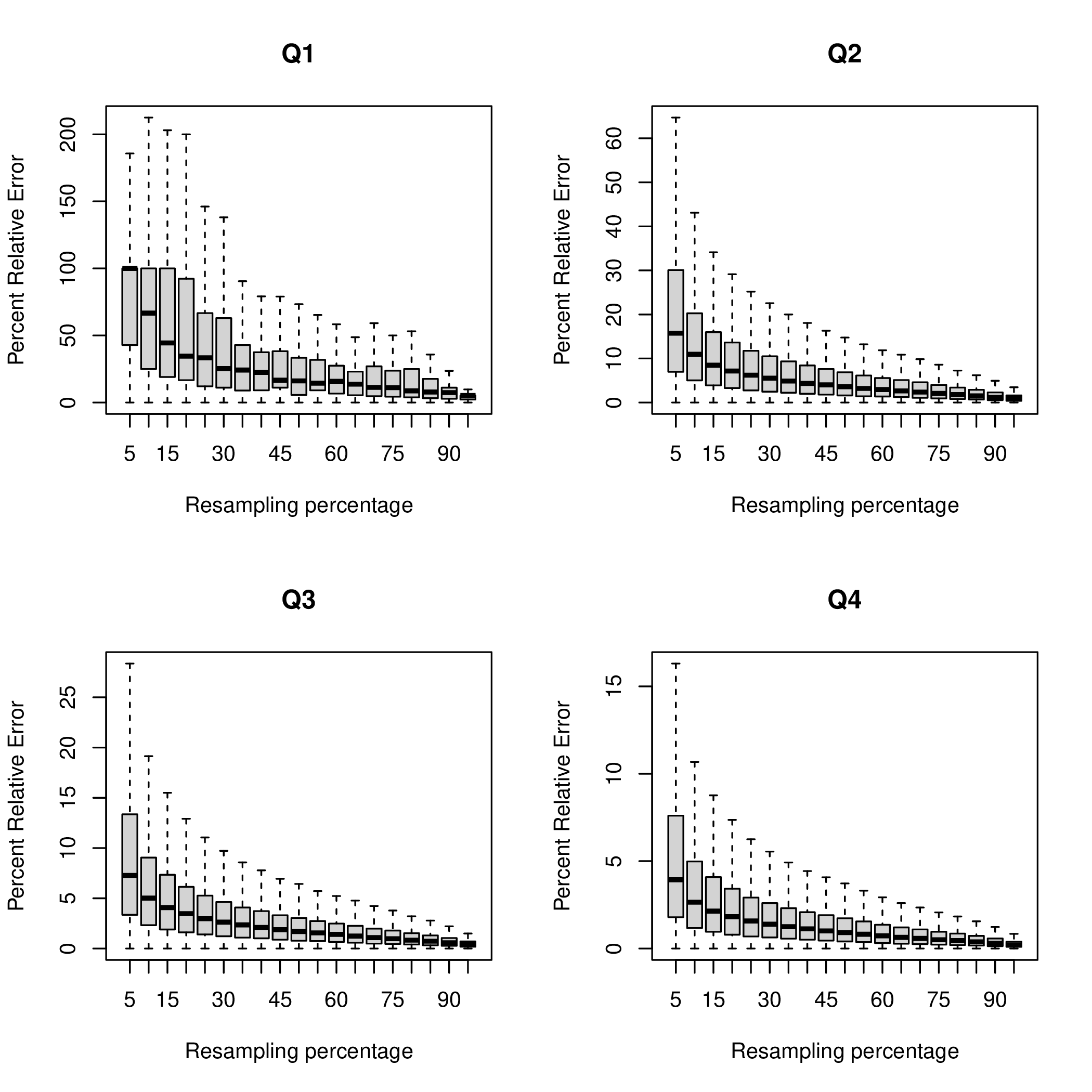

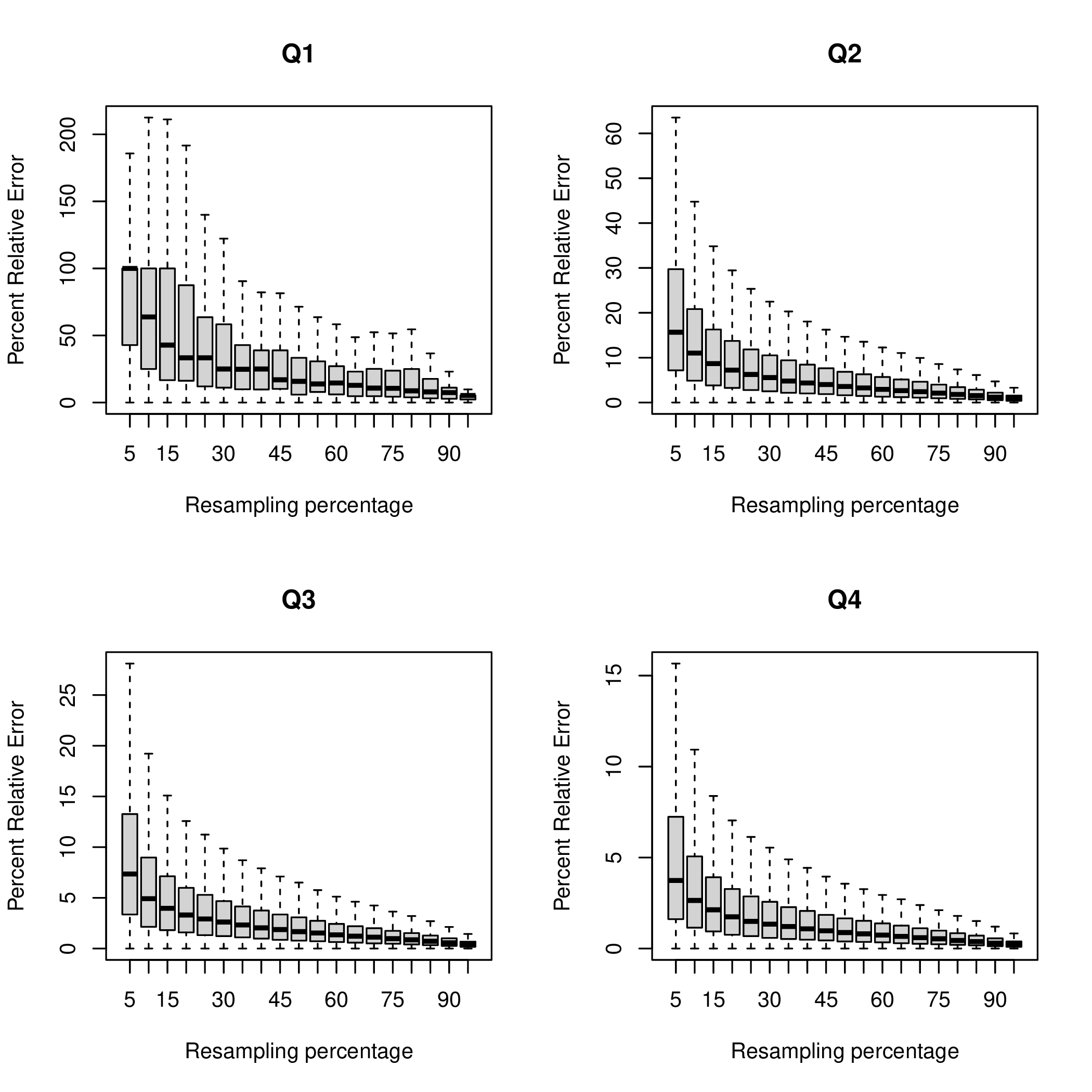


Supplementary Figures 15：T3.saturation Supplementary Figures 16：T4.saturation
